# Can Sunspot Activity Affect the Population Dynamics of Cotton Bollworm, *Helicoverpa armigera* (Hübner) (Lepidoptera: Noctuidae)?

**DOI:** 10.3390/insects16080846

**Published:** 2025-08-15

**Authors:** Jian Huang, Xiaojun Wang

**Affiliations:** 1 Institute of Desert Meteorology, China Meteorological Administration, Urumqi 830002, China; wangxj@idm.cn; 2 Wulanwusu National Special Test Field for Comprehensive Meteorological Observation, Urumqi 830002, China; 3 Wulanwusu Ecology and Agrometeorology Observation and Research Station of Xinjiang, Urumqi 830002, China

**Keywords:** cotton bollworm, sunspot, abrupt change, relative catch

## Abstract

Whether there is a correlation between sunspots and an impact on insects has long been a subject of debate. As a worldwide pest, the cotton bollworm can feed on a variety of plants and cause great harm to agriculture. Clarifying the pattern of sunspots’ influence on it is of great significance for the integrated management of pests. Therefore, we analyzed the sunspot data from 1989 to 2018, as well as the cotton bollworm data from three counties: Maigaiti and Bachu in southern Xinjiang (with 29 and 25 years of data, respectively) and Shawan in northern Xinjiang (with 23 years of data), China. The population size of cotton bollworms increased with the rise in annual mean temperature (*T*_mean_) in the three regions. The impacts of sunspots on annual *T*_mean_ in Maigaiti and Bachu were not significant, while in Shawan, there was a lagged effect. Sunspot activity was significantly negatively correlated with the number of moths in Maigaiti and Shawan, while the influence of sunspots on the Bachu population exhibited a certain degree of lag. The fluctuation cycles of moth numbers in these three regions were not consistent with those of sunspots. In Maigaiti and Shawan, the increase in annual, monthly, and daily sunspot numbers was negatively correlated with the rise in annual, monthly, and daily moths captured, respectively, while in Bachu, they were positively correlated. The cycle of sunspots was not synchronized with the cycles of annual *T*_mean_ in the three regions. When the number of sunspots exceeded 100, the relative catch (RC) of moths rose in Bachu, while the RC of moths decreased in Maigaiti and Shawan. The RC of moths did not at all increase as the number of sunspots rose. The abrupt changes in the annual *T*_mean_ and moth numbers both occurred later than the abrupt change in sunspots, indicating that the influence of sunspots on them had a certain degree of lag and was not synchronous.

## Simple Summary

Whether there is a correlation between sunspots and an impact on insects has long been a subject of debate. As a worldwide pest, the cotton bollworm can feed on a variety of plants and cause great harm to agriculture. Clarifying the pattern of sunspots’ influence on it is of great significance for the integrated management of pests. Therefore, we analyzed the sunspot data from 1989 to 2018, as well as the cotton bollworm data from three counties: Maigaiti and Bachu in southern Xinjiang (with 29 and 25 years of data, respectively) and Shawan in northern Xinjiang (with 23 years of data), China. The results showed that the impact of sunspots on local temperature was insignificant. Sunspots had an impact on cotton bollworms, and there might be a lag effect in some regions. The cycle of sunspot changes was not entirely consistent with that of cotton bollworm population changes. As the number of sunspots increases, the capture rate of cotton bollworms would decrease. The abrupt changes in sunspots occurred before abrupt changes in temperature and cotton bollworm populations, and its influence on the latter two exhibited a certain degree of lag.

## 1. Introduction

The Sun is the primary source of energy for the Earth and other planets. Sunspots exhibit an 11-year periodic variation, and the climate also changes frequently. Whether sunspots and weather are related remains controversial [1]. Solar variability may still be contributing to ongoing climate change [2]. Hassan et al. [3] pointed out that solar activity, manifested in the form of sunspots, had a significant impact on the Earth’s climate. Nasution et al. [4] suggested that there was a negative correlation between sunspot numbers and land temperatures as well as global temperatures, and that solar activity might play a role in shaping the Earth’s temperature fluctuations. However, there is no convincing evidence of a statistically significant or practically useful correlation between sunspot cycles and the weather or climate [5]. Furthermore, the changes in sunspots cannot cause climate warming [6]. Khezri [7] regarded that sunspot activity, the length of day and the Global Mean Sea Level had negligible impacts on global temperature variations. It is essential to understand whether there is a correlation between sunspot activity and local climate, and this is of great significance for integrated pest management.

This debate also extended to discussions on the correlation between sunspots and insects. Some studies suggested that there were correlations between sunspots and insects. Blunck and Wilbert [8] assumed that insect gradations might be related to solar activity. Myers [9] regarded that periods of synchrony of population outbreaks and cool temperatures appeared to be associated with troughs in the sunspot cycle. Spring, summer and winter temperatures covary with sunspot activity, but the mechanisms through which solar activity could affect population dynamics of forest insects are not clear [10]. There is a strong negative relationship between sunspot numbers and population indices of autumnal and winter moths [11]. The periodicity of massive outbreaks of the meadow moth (*Loxostege sticticalis*) in China is correlated with the odd-numbered cycles of sunspot activity [12]. Even a strong, linear relationship exists between shading and the temperature of the first-instar colonies of *Thaumetopoea pinivora* [13]. Nowinszky et al. [14] illustrated that more Bordered Straw (*Helicoverpa armigera* Hübner) moths could be trapped when sunspot number was over 30. However, other studies hold different views. Zverozomb-Zubovsky [15] concluded that the uneven intervals between the outbreaks and their asynchronous appearance in different parts of the species range were inconsistent with the solar concept. Nilssen et al. [16] regarded that solar activity could not explain the reasons for fluctuations in population outbreaks. Láska and Rogl [17] insisted that the period of the fluctuations of carrot psyllid (*Trioza apicalis*) being connected with climatic conditions via sunspot activity was not evident statistically. Among these studies, only Nowinszky [14] focused on the cotton bollworm (*Helicoverpa armigera*); the other studies had short research periods or included data from a single region, thus lacking multi-year and multi-locality studies on the relationship between the cotton bollworm and sunspot activity.

The cotton bollworm, *Helicoverpa armigera* (Hübner) (Lepidoptera: Noctuidae), is polyphagous, with high fecundity, facultative diapause, and high mobility [18], and is one of the most serious insect pests in the world [19]. It usually produces 4–7 generations in a year in China,; for example, it produces 4 generations a year in southern Xinjiang and 3 generations a year in northern Xinjiang, China, with the first generation mainly utilizing wheat and other generations utilizing other plants such as cotton and corn [18]. China produced the highest cotton yield worldwide in 2012, and half of that was produced in the Xinjiang Uygur Autonomous Region [20]. It is necessary to understand the population dynamics of cotton bollworm for integrated pest management.

There were differences in topographical environments across various regions, and the impact of sunspot activity on temperature changes varied accordingly. For example, 25–56% of the temperature increase recorded by local meteorological stations in Norway may be related to solar activity, while for three North Atlantic meteorological stations, the contribution of solar activity to the temperature rise ranges from 63% to 72% [21]. Cotton bollworm is an ectothermic organism, and its flight is influenced by environmental temperature. The varying temperature responses to sunspot activity across different regions significantly affect temperature changes, thereby influencing the growth and development of the cotton bollworm, and even the number trapped.

Thus, the main objectives of this study are as follows: (1) Is there a correlation between sunspot activity and local temperature? (2) Does sunspot activity affect the population dynamics of the cotton bollworm? (3) Are the fluctuation cycles of sunspot activity consistent with those of the cotton bollworm population? (4) What is the relationship between changes in sunspot numbers and the capture rate of the cotton bollworm? (5) Are the abrupt changes in sunspots, temperature, and the cotton bollworm population synchronous?

## 2. Materials and Methods

### 2.1. Study Sites

**Figure 1:**
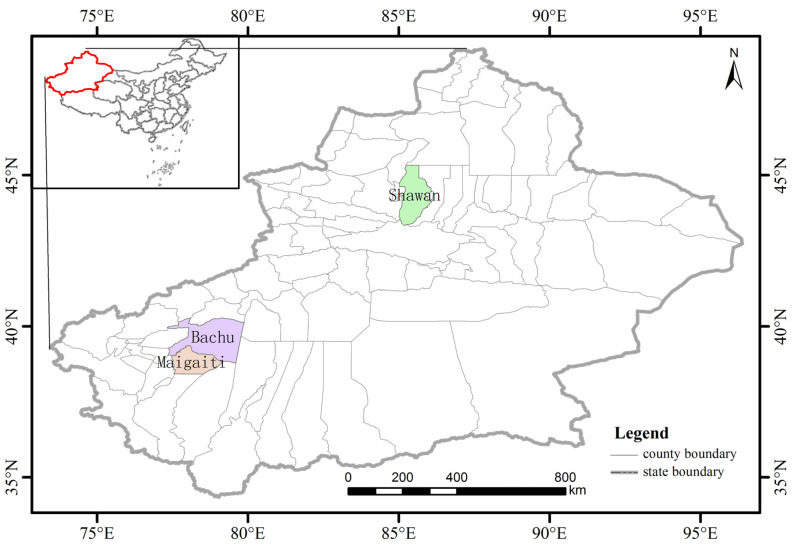
The three study sites (Maigaiti, Bachu, and Shawan) are located in Xinjiang Uygur Autonomous Region, China. The total area of the three counties is 45,864.3 km^2^. The figure was generated using AicGis 10.5 (http://www.arcgis.com, accessed on 8 July 2025).

The study sites were Maigaiti county, Bachu county, and Shawan county (Figure 1). The total area of the three counties is 45,864.3 km^2^ [22]. The straight-line distances from Shawan to Bachu and to Maigaiti are approximately 1100 km and 1200 km, respectively.

Maigaiti county (77°28′–79°05′ E, 38°25′–39°22′ N and 1155–1195 m above sea level) (Figure 1), with a total area of 11,023 km^2^, lies in the western Tarim Basin, Xinjiang Uygur Autonomous Region (XUAR), China [22], and it includes a town and several villages but has no hills or mountains. The area has a temperate continental arid climate, and the annual mean temperature (*T*_mean_), annual mean precipitation, sunshine hours, and mean frost-free period are 11.7 °C, 48.2 mm, 2,806 h, and 214 d, respectively [22].

Bachu county (38°47′–40°17′ N, 77°22′–79°56′ E, and 1100–1180 m above sea level) (Figure 1), with a total area of 21,741.3 km^2^, lies in the western Tarim Basin, XUAR, China [22], and comprises a town and several villages. The area has a temperate continental arid climate, and the annual *T*_mean_, annual minimum air temperature, annual maximum air temperature, annual precipitation, annual sunshine hours, and annual mean frost-free period are 11.9 °C, −6.2 °C (January), 26 °C (July), 58.9 mm, 2994 h and 255 d, respectively [22].

Shawan county (84°56′–86° 08′ E, 43°19′–45°55′ N and 1290–3867 m above sea level) (Figure 1), with a total area of 13,100 km^2^, lies in the southern Junggar Basin, XUAR, China [22], and includes a town and several villages. The Tianshan Mountains lie in southern Shawan County. The area has a temperate continental arid climate and a vertical climatic zone, and the annual *T*_mean_, annual mean precipitation, sunshine hours, and annual mean frost-free period are 6.6 °C, 190 mm, 2835 h, and 180 d, respectively [22].

### 2.2. Weather and Cotton Bollworm Moth Survey Data and Sunspot Data

Weather parameters were recorded at the Maigaiti, Bachu, and Shawan weather administrations, which lie on the edge of the counties. The adult moths were trapped with a 20 W black light lamp (made by Jiaduo Technology, Industry and Trade Company Limited, Hebi, China), which was placed in an open field at 1.5 m above the ground, with no trees or higher buildings surrounding the lamp. The distance between the weather station and the lamp was approximately 300 m. The lamp was turned on at dusk and off after dawn the next day: in Shawan, from early April to late September, 1996–2018; in Bachu, from early April to late October, 1991–2015; and in Maigaiti, from early April to late October, 1989–2017. Cotton bollworm moths were distinguished using standard methods [23,24]. Sunspot data were taken from the World Data Center SILSO, Royal Observatory of Belgium, Brussels (https://www.sidc.be/SILSO/datafiles, accessed on 9 May 2025).

### 2.3. Statistical Methods

The trends over time in the temperature and number of moths captured during the study period were analyzed using linear regression. The relationships between moths captured, temperatures, and sunspots were determined using Pearson correlation analyses and regression functions by SPSS 13.0. Statistical significance was declared at *p* < 0.05.

The nonparametric Mann–Kendall (MK) test was first developed by Mann [25] and further developed by Kendall [26] and Gerstengarbe and Werner [27]. The MK test presents the merits of a broad detection range, small artificial impact, and high degree of quantitativeness [28]. In this study, the MK test procedure with a 5% significance level was applied to analyze abrupt changes according to Gerstengarbe and Werner [27].

When the number of moths trapped was 0, the moth count for that day was recorded as 0 and excluded from the analysis. In this study, the daily moth count was logarithmically transformed using Sturges’ method [29], denoted as follows: log_2_ (moth number) +1. A total of 81,743 moths were collected over 6724 nights in these three regions.

The number of cotton bollworm moths varied across different years and three regions. Thus, relative catch (RC) values were calculated from number of moths captured. The RC value refers to the average number of individuals per sampling time unit (generally it is one night) [30]. The daily sunspot number ranged from 0 to 397 during 1989–2018; thus they were divided into 18 phases (0≤ S ≤ 10, 10 < S ≤ 20, 20 < S ≤ 30, 30 < S ≤ 40, 40 < S ≤ 50, 50 < S ≤ 60, 60 < S ≤ 70, 70 < S ≤ 80, 80 < S ≤ 90, 90 < S ≤ 100, 100 < S ≤ 125, 125 < S ≤ 150, 150 < S ≤ 175, 175 < S ≤ 200, 200 < S ≤ 225, 225 < S ≤ 250, 250 < S ≤ 275, 275 < S; where S meant sunspot number) to calculate mean RC values for each phase.

## 3. Results

### 3.1. Relationship Between Annual T_mean_ and Cotton Bollworm Moths

**Figure 2:**
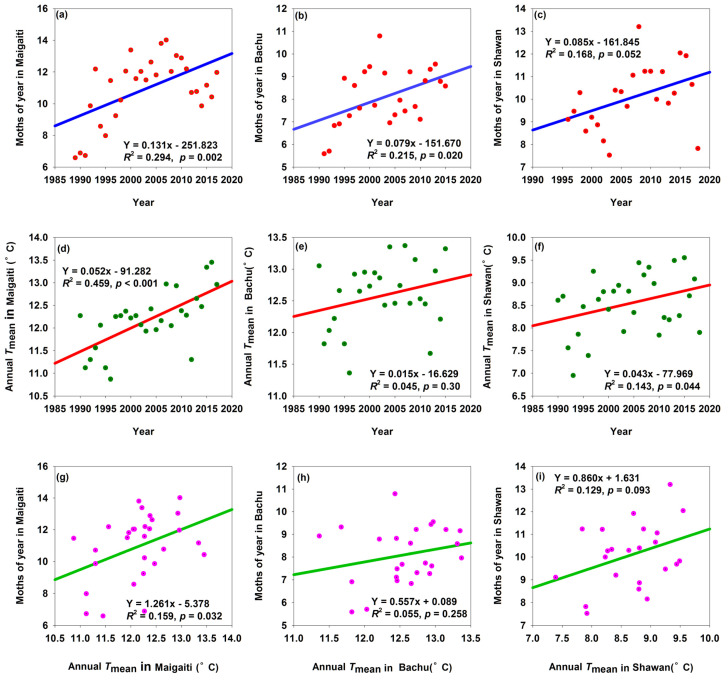
Trends of changes in moth numbers and annual *T*_mean_ (**a**–**f**), and relationships between moth numbers and annual *T*_mean_ in Maigaiti, Bachu, and Shawan (**g**–**i**).

The number of cotton bollworm moths (Table S1) showed increasing trends (Figure 2a–c), with significances in Maigaiti and Bachu (Figure 2a,b). In Maigaiti, the number of moths increased by 0.131 per year, followed by in Shawan, which increased by 0.085 per year. Annual *T*_mean_ also showed increasing trends (Figure 2d–f). The annual *T*_mean_ in Maigaiti and Shawan significantly increased by 0.052 °C and 0.043 °C per year, respectively (Figure 2d,f). In Maigaiti, Bachu, and Shawan, the number of moths increased by 1.261, 0.557, and 0.860 for every 1 °C rise in annual *T*_mean_, respectively (Figure 2g–i), though only the relationship between annual *T*_mean_ and moths in Maigaiti was significant (Figure 2g). These results showed that an increase in annual *T*_mean_ contributed to an increase in moth number.

### 3.2. Relationship Between Sunspot and Annual T_mean_

**Figure 3:**
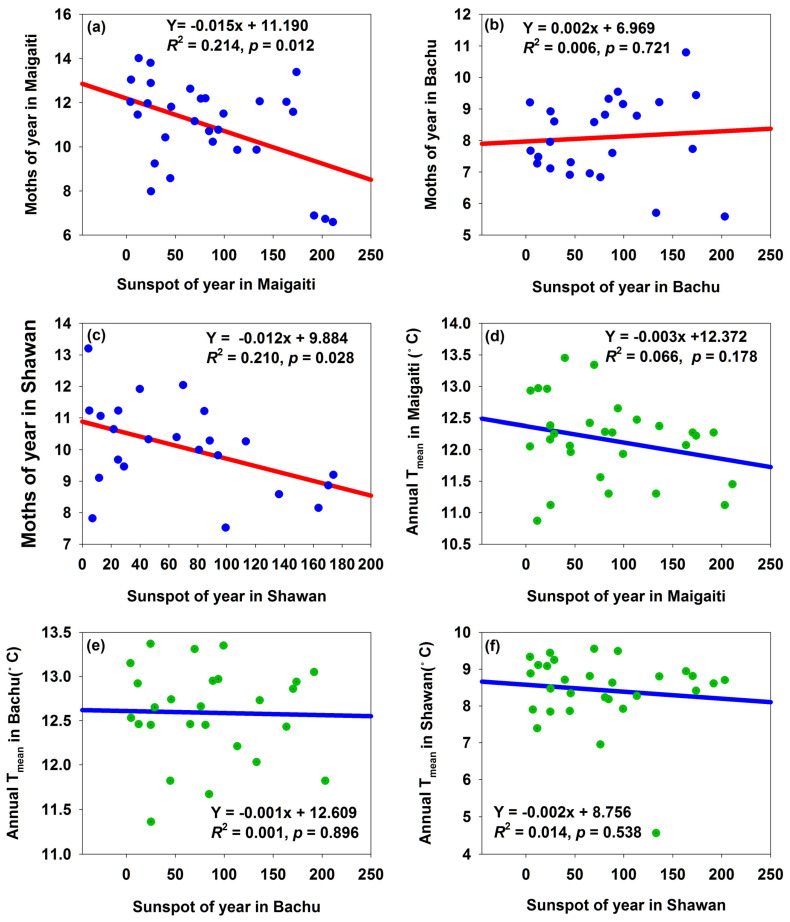
The relationships between sunspots and moth numbers in Maigaiti (**a**), Bachu (**b**), and Shawan (**c**), respectively. The relationships between sunspots and annual *T*_mean_ in Maigaiti (**d**), Bachu (**e**), and Shawan (**f**), respectively.

The number of moths in Maigaiti and Shawan (Table S1) significantly decreased by 0.015 and 0.012, respectively, with each 1-unit increase in sunspot numbers, (Figure 3a,c), while the number of moths in Bachu (Table S1) showed an insignificant increasing trend with sunspot numbers (Figure 3b). Meanwhile, increased sunspot numbers were associated with a decrease in annual *T*_mean_, though the relationships were not significant (Figure 3d–f). A correlation analysis was conducted between the sunspot numbers and annual *T*_mean_ of the three regions (with an advanced time of 1 to 6 years and a lagged time of 1 to 6 years. Here, “advanced” refers to analyzing the current year’s annual *T*_mean_ in relation to sunspot data from 1 to 6 years prior; “lagged” refers to analyzing the current year’s annual *T*_mean_ in relation to sunspot data from 1 to 6 years later. See Table 1). The results showed that only the annual *T*_mean_ in Shawan had a significant negative correlation with sunspot numbers when advanced by 2 years; in all other cases, the correlations were not significant, regardless of whether the annual *T*_mean_ was advanced or lagged by 1–6 years (Table 1). This indicated that sunspot activity had no significant impact on the annual *T*_mean_ of the three regions.

### 3.3. The Relationship Between the Periodic Variations in Sunspot Numbers and Moth Numbers

**Figure 4:**
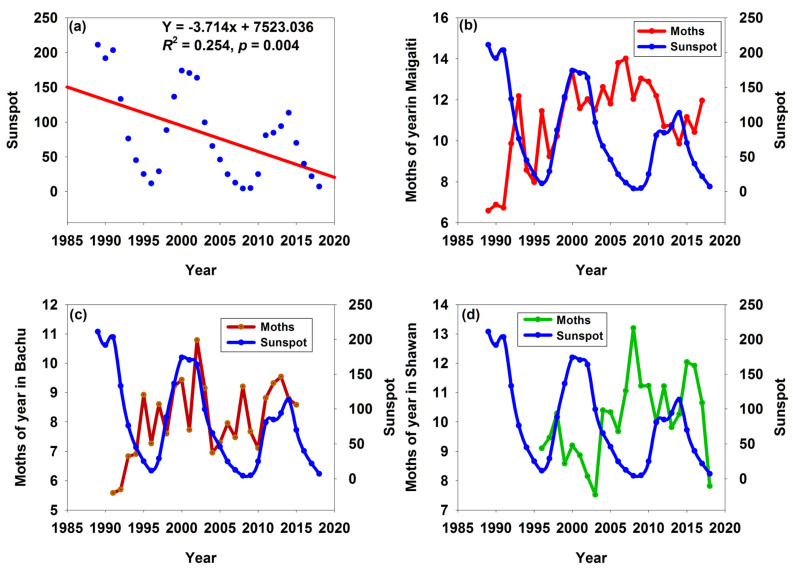
Trend of sunspots (**a**) and annual changes in moth numbers and sunspots in Maigaiti, Bachu, and Shawan (**b**–**d**).

Between 1989 and 2018, the maximum values of three successive sunspot cycles (Table S1) decreased sequentially, indicating that the intensity of solar activity was weakening, and sunspot numbers decreased significantly by 3.714 for each year (Figure 4a). However, in these three regions, only some years showed consistent trends between the moth numbers and sunspots (Table 2). Moths in Shawan had the most years with consistent trends (13 years) and the highest proportion (56.52%), followed by those in Bachu (12 years and 48.00%, respectively); moths in Maigaiti had the fewest years (10 years) and the lowest proportion of (34.48%). There were even instances where moth numbers reached their peak when sunspot numbers were at their lowest (Figure 4b,d), and conversely, moth numbers hit their lowest point when sunspot numbers were at their highest (Figure 4b,c). These results indicated that the fluctuation cycles of moth numbers in these three regions were not consistent with those of sunspots.

**Figure 5:**
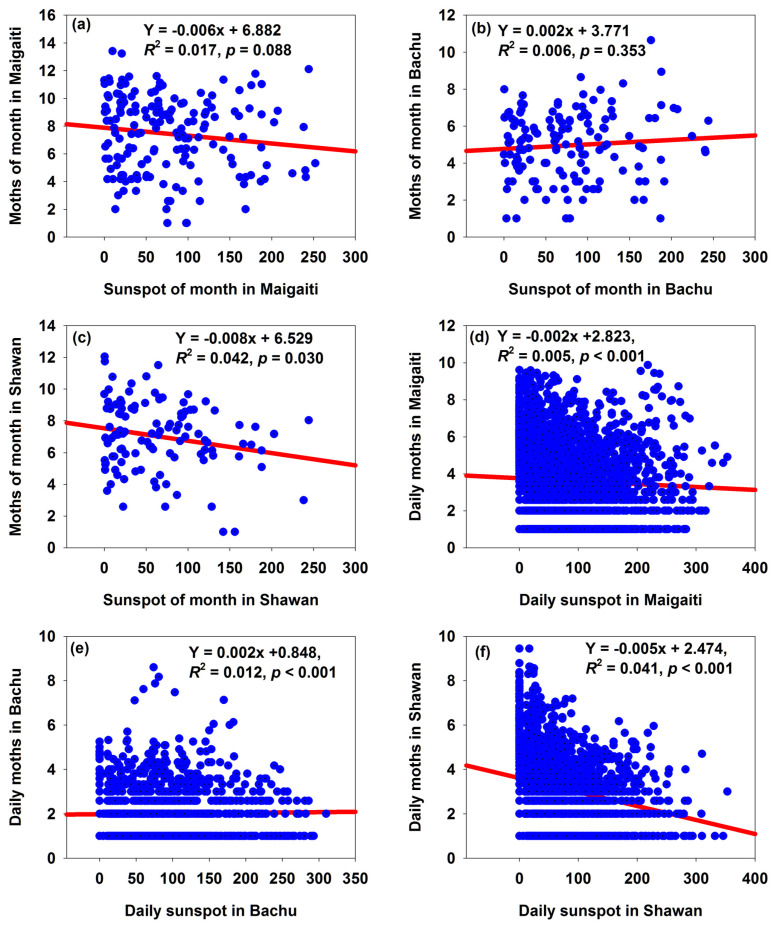
Relationships between monthly moth numbers and monthly sunspot numbers in Maigaiti, Bachu, and Shawan (**a**–**c**); relationships between daily month numbers and daily sunspot numbers in the same regions (**d**–**f**).

From the perspective of monthly variations, sunspots and moth numbers (Table S2) showed either a positive correlation (Figure 5b) or negative correlations (Figure 5a,c), though only the relationship between sunspots and moth numbers in Shawan was significant (Figure 5c). It hinted that an increase in the monthly number of sunspots could increase or decrease moth numbers, indicating uncertainty.

From the perspective of daily variations, sunspots and moth numbers (Table S3) showed either a positive correlation (Figure 5e) or negative correlations (Figure 5d,f); all the relationships between sunspots and moth numbers in these three regions were significant (Figure 5d–f). This indicated that an increase in daily sunspot numbers could either increase or decrease moth numbers, also showing uncertainty.

In Bachu, whether in terms of annual, monthly, or daily variations in sunspots, an increase in sunspots was accompanied by a rising trend in moth numbers (Figure 3b and Figure 5b,e). For Maigaiati and Shawan, however, whether in terms of annual, monthly, or daily variations in sunspots, an increase in sunspot numbers was accompanied by a decreasing trend in moth numbers (Figure 3a,c and Figure 5a,c,d,f). This might imply that the impact of one of the annual, monthly, or daily variations in sunspots on the moth population was consistent with those of the other two.

A correlation analysis was performed between the population sizes of the three regions (with an advance time of 1 to 6 years and a lagged time of 1 to 6 years) and sunspot numbers (Table 3). The results showed that the population size in Maigaiti had a significant negative correlation with sunspot numbers when advanced by 1 year, without advancement, and when lagged by 1 year; the population size in Bachu only exhibited a significant negative correlation with sunspot numbers when advanced by 3 years; and the population size in Shawan showed a significant negative correlation with sunspot numbers when advanced by 1 year and without advancement (Table 3). This suggested that sunspot activity might have a significant impact on the cotton bollworm population sizes in the three regions, and for some regions there was a lagged effect.

### 3.4. The Asynchrony Between Sunspot and Changes in Annual T_mean_ and Moth Numbers

**Figure 6:**
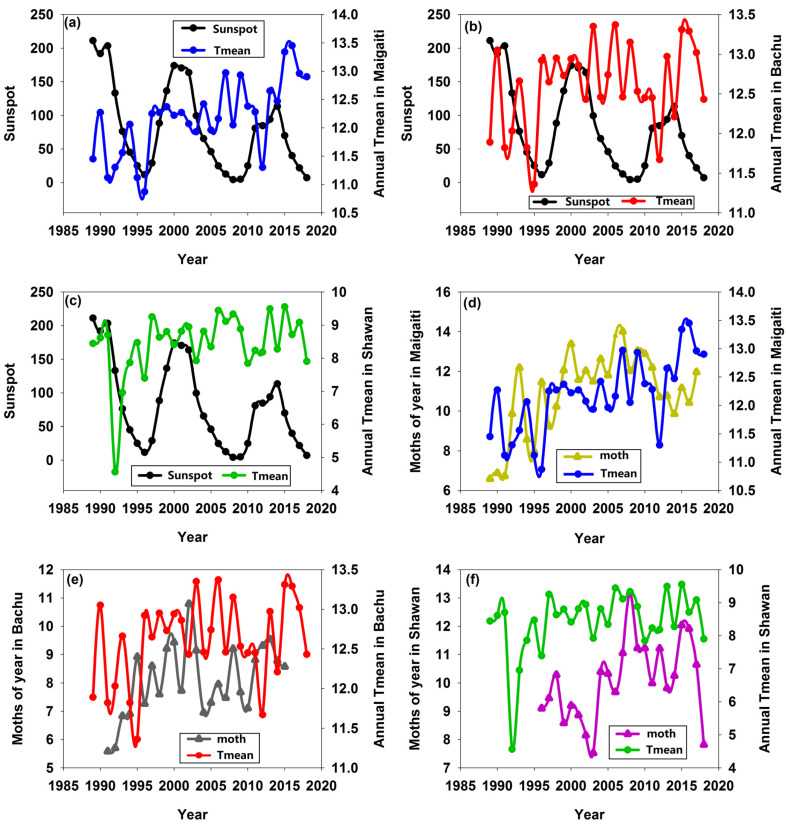
Change cycles of sunspots and annual *T*_mean_ (**a**–**c**), and change cycles of moth numbers and annual *T*_mean_ (**d**–**f**) in Maigaiti, Bachu, and Shawan, respectively.

When the number of sunspots was at its minimum in 2008, the annual *T*_mean_ in the three regions were relatively high (Figure 6a–c). The lowest annual *T*_mean_ in Maigaiti, Bachu, and Shawan appeared in 1996, 1995, and 1992, respectively, and the minimum number of sunspots occurred in 2008, while a relatively small number of sunspots appeared in 1996. This indicated that the years of the lowest annual *T*_mean_ in these three regions appeared much earlier than that of the minimum number of sunspots. From 1989 to 2018, there were three distinct declining phases of sunspots, namely 1989–1996, 2000–2009, and 2014–2018, as well as two distinct rising phases: 1996–2000 and 2009–2014. However, annual *T*_mean_ in these three regions did not show such distinct rising and falling phases. It suggested that the cycle of sunspot was not synchronized with the cycles of annual *T*_mean_ in the three regions.

Regarding the fluctuation cycles of annual *T*_mean_ and moth populations, the Shawan population showed a high degree of similarity to annual *T*_mean_ changes (Figure 6f), followed by the Maigaiti population (Figure 6a). Except for the two periods of 1990–1995 and 2010–2015 when there was a mismatch, the Bachu population showed a high degree of similarity to annual *T*_mean_ changes in the remaining years (Figure 6b). This might imply that there was a certain correlation between the annual *T*_mean_ change cycle and the moth population change cycle, that is, annual *T*_mean_ affected the size of the moth population.

### 3.5. Impact of Sunspots on Moths Captured

**Figure 7:**
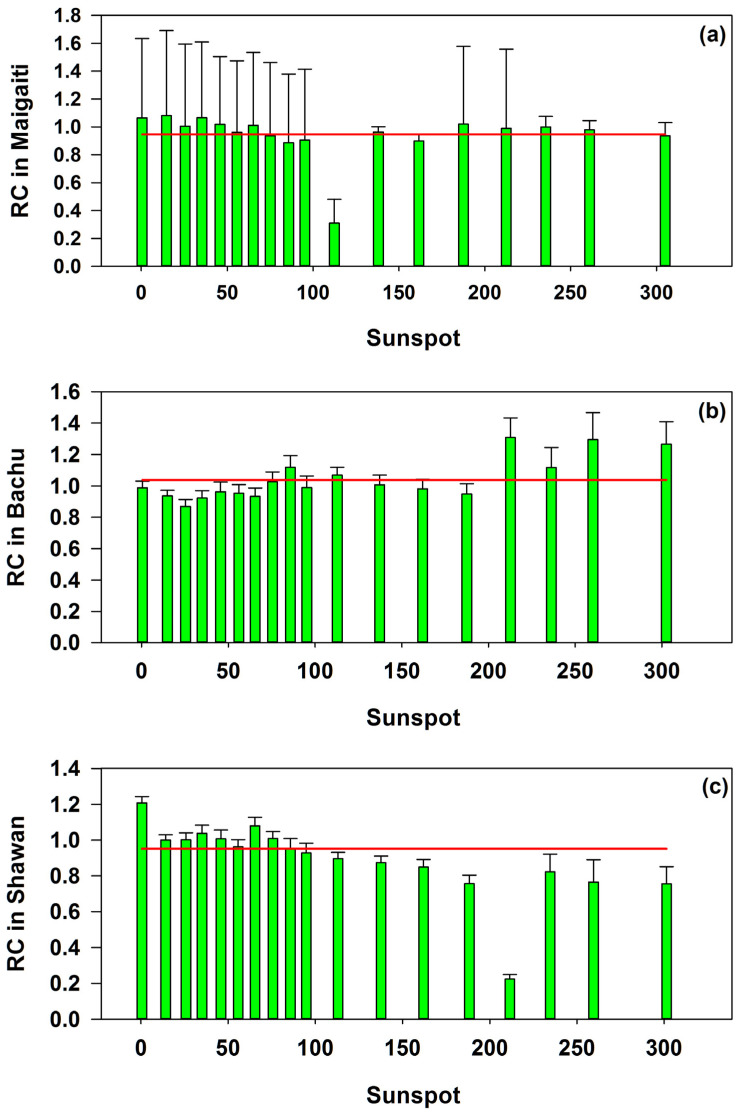
RC values of moths in Maigaiti (**a**), Bachu (**b**), and Shawan (**c**). The solid red line was the mean value of RC values, and these values were 0.9469, 1.0384, and 0.9519 in Maigaiti, Bachu, and Shawan, respectively.

Mean RC values were 0.9469, 1.0384, and 0.9519 in Maigaiti, Bachu, and Shawan, respectively (Figure 7). The number of phases that the RC values (Table S4) were lower than the average values in Maigaiti, Bachu, and Shawan were 6, 12, and 10, respectively. In Bachu, there were only six phases where the RC values were higher than the average, yet five of these phases occurred in periods when sunspot numbers exceeded 100 (Figure 7b). Correspondingly, in Maigaiti, there were 12 phases where the RC values were higher than the average, of which only 5 phases occurred in periods when sunspot numbers exceeded 100, and 7 phases occurred in periods when sunspot numbers were less than 100 (Figure 7a). Similarly, although there were eight phases in Shawan where the RC values were higher than the average, all of these phases occurred in periods when sunspot numbers were less than 100, while the RC values in periods when sunspot numbers exceeded 100 were all lower than the average (Figure 7c). These indicated that the RC of the Shawan population dropped significantly when the sunspot numbers were over 100, while the RC of Maigaiti population showed a slight decrease and the RC of Bachu population showed a rapid increase. That is, solar sunspot activity has different effects on the populations of these three regions.

In Maigaiti, Bachu, and Shawan, when the sunspot numbers were less than 100, the proportions of moths captured during this period to the total number of captured moths were 75.19%, 53.43%, and 86.86%, respectively, and the proportions of capture days to the total number of days in Maigaiti, Bachu, and Shawan were 70.17%, 66.03%, and 75.47%, respectively (Table 4). In particular, when sunspot numbers were less than 30, the number of moths captured in Maigaiti and Shawan accounted for approximately half of the total, at 46.43% and 54.46%, respectively (Table 4). This meant that when sunspot numbers were higher than 100, the proportion of captured moths was very small. This was particularly true for Maigaiti and Shawan, accounting for 24.81% and 13.14%, respectively; only Bachu had a relatively high proportion, at 46.57% (Table 4). It hinted that most moths were captured when sunspot numbers are less than 100, while only a small proportion were captured during periods of higher sunspot numbers. These above results suggested that the view that the increase in the number of captured moths with the rise in sunspot numbers was only supported by the results in Bachu (Figure 7b, Table 4).

In terms of the daily average number of moths captured (ANC), the highest values in Maigaiti and Shawan occurred when sunspot numbers were less than 30, at 24.52 and 12.99, respectively, while the highest value in Bachu appeared when sunspot numbers were greater than 200, at 6.08 (Table 4). This indicated that the impact of sunspot activity on moth trapping numbers varied across different regions.

### 3.6. MK Test of the Asynchrony

**Figure 8:**
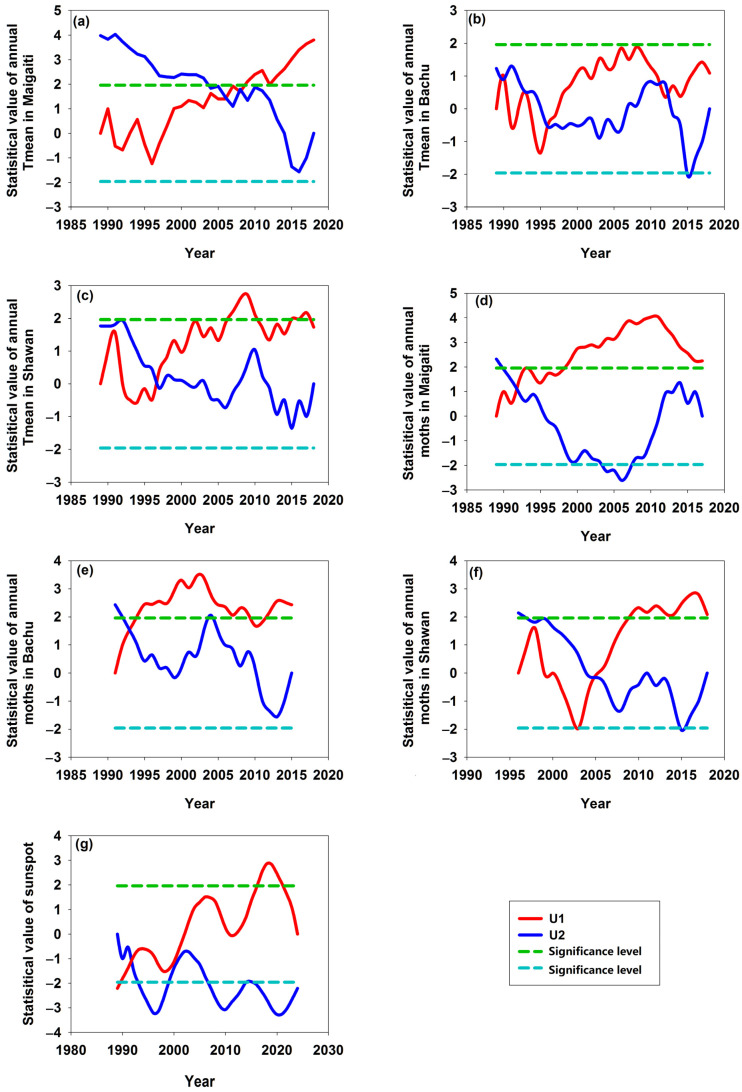
Abrupt changes in annual *T*_mean_ (**a**–**c**), annual moth numbers (**d**–**f**), and sunspots (**g**). A solid red line (*U*_1_) and a solid blue line (*U*_2_) represent the forward and opposite directions of the calculated result of the series, respectively. A dashed green line (*P*_1_) and a dashed pale blue line (*P*_2_) represent the significance level at 5% (with *P* = 1.96 and *P* = −1.96), respectively, viz.,|*P*|< 1.96.

The MK test indicated that the significantly abrupt changes in the annual *T*_mean_ of Maigaiti, Bachu, and Shawan occurred in 2005, 1996, and 1996, respectively (Figure 8a–c), and that the significantly abrupt changes in moth numbers in Maigaiti, Bachu, and Shawan occurred in 1991, 1993, and 2004, respectively (Figure 8d–f). However, the significantly abrupt change in sunspot number occurred in 1991 (Figure 8g). The earliest abrupt change in the annual *T*_mean_ was 1995 in Bachu, and the earliest abrupt change in the moth number was 1991 in Maigaiti. However, none of them occurred earlier than the abrupt change in sunspot number. This indicated that their abrupt changes were not synchronized. It might be speculated that sunspots had a lag effect on annual *T*_mean_ and moth populations.

## 4. Discussions

Richmond [31] suggested that sunspots affected the weather, which in turn affected the abundances of insects. In our study, there were insignificantly negative correlations between the number of sunspots and the annual *T*_mean_ (Figure 3d–f). However, as the number of sunspots increased, the number of moths decreased (Figure 3a,c), while only the number of moths in Bachu increased (Figure 3b). This was because the relationship between changes in sunspot numbers and temperature was not simply a case of “more sunspots mean higher temperatures”; instead, it was influenced by multiple factors such as the overall patterns of solar activity and the Earth’s atmospheric system, showing a complex correlation. In addition, after removing the influence of solar activity, the trend of solar output does not match the long-term trend of global temperature; the reason for the pause in temperature rise may also be that the heat generated by the increase in greenhouse gas concentrations has been absorbed by the deep ocean layers [32]. Myers [9] suggested that troughs in the cycles of sunspot activity could be associated with cooler temperatures. However, judging from the fluctuation cycles of sunspot numbers and annual *T*_mean_, their cycles sometimes matched, sometimes did not match, and even showed opposite trends. Especially between 2000 and 2009, sunspot numbers decreased significantly, while the temperature remained high (Figure 6a–c), and the moth numbers also stayed at a high level (Figure 4b–d) even if this period was in the troughs in the cycles of sunspot activity. This indicated that the cycle of sunspot activity was not synchronized with either the annual *T*_mean_ cycle or the moth population cycle, whereas the annual *T*_mean_ cycle and the moth population cycle were synchronized with each other (Figure 6d–f). This observation was further supported by Figure 2. In addition, during 1996–2000, sunspot numbers were increasing, but the temperature rise was advanced by 1–3 years (Figure 6a–c). Therefore, the increase in sunspot numbers could not be the sole or primary condition for a rise in temperature. Meanwhile, our research indicated that sunspots had no significant impact on the annual *T*_mean_. The annual *T*_mean_ fluctuation cycle was highly similar to that of captured moth numbers, but the sunspot fluctuation cycle did not exhibit such a high degree of similarity to the fluctuation cycle of captured moth numbers.

What was the extent of the impact of sunspots on moth trapping? Nowinszky et al. [14] regarded that when sunspot number was below 30, no verifiable effect on the efficiency of light trapping of the Scarce Bordered Straw had been found, while when the number of sunspots exceeded 30, a higher number of sunspots led to increase in trapping catches. However, in our study, when the number of sunspots was less than 30, the number of moths caught in Maigaiti and Shawan accounted for nearly half. When the number of sunspots was between 30 and 100, the number of moths caught in these two counties decreased slowly. When the number of sunspots exceeded 100, the proportion of moths caught in Maigaiti and Shawan dropped sharply (Figure 7a,c, Table 4). However, this was the opposite in terms of the number of moths caught in Bachu (Figure 7b, Table 4). Thus, only the data in Bachu supported the view of Nowinszky et al. [14], and other data in Maigaiti and Shawan did not support this point. Therefore, we did not fully endorse the view that the capture quantity increased when sunspot numbers were greater than 30.

In terms of the average number of moths captured per day (ANC), the three regions showed different trends (Table 4). The population in Maigaiti exhibited a pattern of first decreasing, then rebounding, and then decreasing again; the population in Bachu presented a mode of first increasing, then decreasing, and then increasing again; while the population in Shawan showed a trend of decreasing in sequence with no rebound in between. In addition, when the ANC in the three regions reached their peaks, the sunspot numbers were not the same (Table 4). This illustrated that the impact of sunspot activity on moth trapping numbers varied across regions.

Although there was no direct research on the light factor in the relationship between sunspots and cotton bollworms, relevant studies had shown that the light factor had a significant impact on cotton bollworms. When female cotton bollworm moths are treated with abnormal light cycles, intermittent light, or different light qualities, their courtship peaks decrease, their courtship rhythms become disordered, the content of sex pheromones declines, and the activity of brain factors is inhibited [33]. Since sunspot activity could affect the light conditions on Earth, it might indirectly have similar effects on cotton bollworms.

Sunspot activity can affect the Earth’s climate, and the climate responses to sunspot activity varied across different regions—especially different geographical environments—which in turn might exert different impacts on cotton bollworms. The annual *T*_mean_ and altitude in Maigaiti, Bachu, and Shawan were 11.7 °C, 11.9 °C, 6.6 °C, 1115–1195 m, 1100–1180 m, and 1290–3867 m, respectively. Maigaiti was located in the plain, Shawan was in the mountainous area, and Bachu was also in the plain but close to the mountainous area; therefore, the changes in their cotton bollworm populations differ (Figure 4b–d). The changes in the cotton bollworm populations in Maigaiti and Bachu were similar (Figure 4b,c) because both regions were located in southern Xinjiang, about 100 km apart, with similar climates, and both had four generations of cotton bollworms per year. In contrast, Shawan was in northern Xinjiang, approximately 1500–1600 km away from the previous two regions, with a different climate, and only had three generations of cotton bollworms per year. The number and proportion of years in which the population change trend was consistent with the sunspot activity trend were the highest in the Shawan population, followed by the Bachu population (Table 2). It could be inferred that the closer the distance to mountainous areas, the more consistent the variation trend of cotton bollworms was with that of sunspot activity. Therefore, geographical environment was a factor that must be considered.

The abrupt change in sunspots occurred in 1991, while the abrupt changes in the annual *T*_mean_ of Maigaiti, Bachu, and Shawan took place in 2005, 1996, and 1996, respectively (Figure 8a–c,g). This indicated that the abrupt change in sunspots occurred earlier than those of the annual *T*_mean_ in the three regions. A possible reason was that the impact of sunspots on annual *T*_mean_ had an insignificant or a lag effect. Solheim et al. [21] regarded that a significant negative trend was found between the length of a cycle and the temperature in the next cycle. Another possible reason was that Shawan was located in mountainous areas, Bachu was close to mountainous areas, and Maigaiti located in the plain; different geographical environments led to different responses of their temperatures to sunspot activity. Meanwhile, the abrupt changes in moth numbers in Maigaiti, Bachu, and Shawan took place in 1991, 1993, and 2004. None of them were earlier than the abrupt year of sunspots (Figure 8d–f,g). The possible reason was that the outbreaks lagged behind the solar activity, because the solar events that lead to the beginning of the increase in density must have occurred a few years before the peak in population abundance [10]. It might be speculated that sunspot activity might have a significant impact on the cotton bollworm population sizes in the three regions, and for some regions there was a lagged effect.

The impact of sunspots on cotton bollworms was likely the result of multi-pathway and multi-factor synergistic effects. Solar activities altered Earth’s physical environment (such as magnetic fields [34], radiation [35], and electric fields [36]) as well as climatic elements (like precipitation and humidity [37]), which in turn directly or indirectly affected the growth, development, reproduction, and ecological interactions of cotton bollworms. The combined effects of these factors might be more complex than the impact of a single factor such as light or temperature. Further interdisciplinary research (e.g., integrating astrophysics, ecology, and insect physiology) is still needed to uncover the specific mechanisms.

## 5. Conclusions

The impact of sunspots on annual *T*_mean_ exhibited a certain lag, while their impact on the cotton bollworm showed a negative correlation. An increase in sunspot numbers did not lead to a higher capture rate for all populations. Currently, research on the effects of sunspots on the cotton bollworm is limited to the capture rate of adults and population dynamics, with few studies reported in other aspects. The following aspects may help further reveal the relationship between sunspots and their impacts on the cotton bollworm:

Study the growth and development indicators of the cotton bollworm at different developmental stages (egg, larva, pupa, adult), such as hatching rate, larval duration, pupation rate, eclosion rate, and adult longevity, to analyze the mechanism by which sunspot activity affects the growth and development process of the cotton bollworm.

Under simulated environments with different sunspot activities, observe reproduction-related indicators of adult cotton bollworms, such as mating behavior, fecundity, and egg quality, to explore the patterns of how sunspot activity affects the reproductive capacity of the cotton bollworm and clarify the relationship between sunspot activity and the reproductive potential of the cotton bollworm.

Study the correlation between the sunspot activity cycle and the fluctuations in population quantity and changes in geographical distribution of the cotton bollworm, and predict the trends of how changes in sunspot activity will affect the future population dynamics of the cotton bollworm.

Starting from meteorological factors, host plants, natural enemies, and other aspects, investigate the mechanism by which sunspot activity indirectly affects the occurrence and development of the cotton bollworm by altering the ecological environment.

Integrate sunspot activity data, meteorological data, biological characteristic data of the cotton bollworm, and ecological environment data, and use methods such as multivariate statistical analysis and machine learning to establish prediction models that can accurately forecast the occurrence period, population size, and damage degree of the cotton bollworm, thereby providing technical support for the early warning and scientific control of the cotton bollworm.

## Figures and Tables

**Table 1:** Cross-correlation analysis of dependence of annual *T*_mean_ in Maigaiti, Bachu, and Shawan on sunspot numbers.

	Maigaiti		Bachu		Shawan	
Lag, Years	Correlation Coefficient (*R*)	*p*	Correlation Coefficient (*R*)	*p*	Correlation Coefficient (*R*)	*p*
−6	−0.184	0.329	0.164	0.531	0.205	0.276
−5	−0.186	0.326	0.005	0.980	0.003	0.312
−4	−0.204	0.281	0.065	0.753	−0.039	0.840
−3	−0.238	0.205	−0.120	0.559	−0.267	0.153
−2	−0.238	0.206	−0.217	0.286	−0.427 *	0.019 *
−1	−0.285	0.127	−0.123	0.550	−0.313	0.092
0	−0.257	0.178	0.027	0.896	0.119	0.538
1	−0.264	0.280	0.027	0.896	−0.090	0.635
2	−0.256	0.172	0.115	0.576	0.040	0.834
3	−0.276	0.146	0.108	0.600	0.054	0.776
4	−0.217	0.250	0.026	0.899	0.065	0.733
5	−0.169	0.371	−0.086	0.678	0.005	0.979
6	−0.138	0.467	−0.163	0.426	−0.703	0.702

**Note:** “−” means advanced, “+” means lagged. Only the annual *T*_mean_ in Shawan showed a significant negative correlation with sunspot numbers when advanced by 2 years; in all other cases, the correlations are not significant regardless of whether the temperature data are advanced or lagged by 1 to 6 years. The significant parts were marked with a *.

**Table 2:** Years when the moth numbers and sunspots showed consistent trends.

Site	Consistent Trend Years	No. of Consistent Trend Years	Proportion of Consistent Trends
Maigaiti	1993–1995,1997–2001,2002–2003, 2004–2005, 2007–2009,2015–2016	10	10/29 = 34.48%
Bachu	1995–1997,1998–2001,2002–2004, 2006–2007,2010–2013,2014–2015	12	12/25 = 48.00%
Shawan	1996–1998,1999–2003,2004–2006, 2011–2012,2013–2014,2015–2018	13	13/23 = 56.52%

**Table 3:** Cross-correlation analysis of dependence of moths captured in Maigaiti, Bachu, and Shawan on sunspot numbers.

	Maigaiti		Bachu		Shawan	
Lag, Years	Correlation Coefficient (*R*)	*p*	Correlation Coefficient (*R*)	*p*	Correlation Coefficient (*R*)	*p*
−6	0.046	0.813	−0.098	0.641	0.105	0.635
−5	0.110	0.570	−0.277	0.180	0.001	0.995
−4	0.105	0.587	−0.339	0.097	−0.147	0.502
−3	−0.064	0.742	−0.433 *	0.031 *	−0.245	0.260
−2	−0.214	0.266	−0.323	0.115	−0.343	0.109
−1	−0.386 *	0.039 *	−0.103	0.623	−0.437 *	0.037 *
0	−0.462 *	0.012 *	0.075	0.721	−0.458 *	0.028 *
1	−0.393 *	0.035 *	0.188	0.367	−0.395	0.062
2	−0.290	0.127	0.251	0.226	−0.274	0.205
3	−0.190	0.323	0.216	0.301	−0.155	0.479
4	−0.092	0.637	0.010	0.964	−0.079	0.721
5	0.019	0.920	−0.123	0.559	−0.027	0.903
6	0.014	0.941	−0.364	0.074	0.106	0.631

**Note**: “−” means advanced, “+” means lagged. The population size in Maigaiti showed a significant negative correlation with sunspot numbers when advanced by 1 year, without advancement, and when lagged by 1 year. The population in Bachu only exhibited a significant negative correlation with sunspot numbers when advanced by 3 years. The population in Shawan had a significant negative correlation with sunspot numbers when advanced by 1 year and without advancement. The significant parts were marked with a *.

**Table 4:** Sunspots and moths captured in Maigaiti, Bachu and Shawan.

Sunspot Number	Item	Maigaiti	Bachu	Shawan
0 ≤ S ≤ 30	PMC	46.43% (26,462)	22.62% (1113)	54.46% (10,795)
	ANC	24.52	2.48	12.99
	PDC	34.94%	29.55%	39.20%
30 < S ≤ 100	PMC	28.76% (16,397)	30.81% (1516)	32.40% (6423)
	ANC	15.07	2.74	8.35
	PDC	35.23%	36.48%	36.27%
100 < S ≤ 150	PMC	11.67% (6649)	27.45% (1351)	8.52% (1688)
	ANC	13.54	4.72	5.63
	PDC	15.90%	18.87%	14.15%
150 < S ≤ 200	PMC	7.93% (4523)	6.01% (296)	3.26% (647)
	ANC	18.09	2.41	4.34
	PDC	8.10%	8.11%	7.03%
200 < S	PMC	5.21% (2968)	13.11% (645)	1.36% (270)
	ANC	16.49	6.08	3.80
	PDC	5.83%	6.99%	3.35%
	Total No. of moths	56,999	4921	19,823

**Note:** S means sunspot number. PMC means the proportion of the number of captured moths to the total number of captured moths, and the numbers in parentheses are the number of moths captured during this period. The total number of moths caught is listed at the bottom of the table. ANC means the average number of moths captured per day. PDC means the proportion of the number of days in this period to the total number of capture days.

## Data Availability

The data presented in this study are available in article and Supplementary Materials.

## Supplementary Materials

The following supporting information can be downloaded at: https://www.mdpi.com/article/10.3390/insects16080846/s1, Table S1: Sunspot, annual *T*_mean_, log_2_(moths number)+1 in three regions; Table S2. log_2_(moths No.)+1 for moth in Maigaiti, Bachu and Shawan; Table S3. Daily log_2_(moths No.)+1 in Magaiti, Bachu and Shawan; Table S4. Relative catching of moths in Maigaiti, Bachu and Shawan.
