# Supplementary material for: Can Sunspot Activity Affect the Population Dynamics of Cotton Bollworm, Helicoverpa armigera (Hübner) (Lepidoptera: Noctuidae)?
Source: Insects. 2025 Aug 15;16(8):846. doi: 10.3390/insects16080846 (PMC12386618; doi:10.3390/insects16080846)
Supplement: Supplementary file 1 [file insects-16-00846-s001.zip › Table S1. Daily log2(moths No.)+1 in Magaiti, Bachu and Shawan.pdf]

Maigaiti	Year	Moth	Date	Sunspot	log2(moths No.)+1	Bachu
	1990	5	5	106	1	
	1990	5	8	183	2	
	1990	5	12	194	1	
	1990	5	13	191	3	
	1990	5	16	208	1	
	1990	5	21	256	2	
	1990	6	7	156	2	
	1990	6	13	177	2	
	1990	6	16	160	3	
	1990	6	23	93	4.169925001	
	1990	6	24	128	1	
	1990	6	25	137	1	
	1990	7	4	308	1	
	1990	7	11	136	2	
	1990	7	22	183	1	
	1990	7	23	211	2	
	1990	7	24	257	2.584962501	
	1990	8	16	292	1	
	1990	8	20	371	2	
	1990	8	21	350	2.584962501	
	1990	8	23	354	3	
	1990	8	25	331	4.321928095	
	1991	5	11	200	1	
	1991	5	24	161	1	
	1991	5	26	191	3	
	1991	5	29	206	1	
	1991	6	23	164	1	
	1991	6	29	234	3.807354922	
	1991	6	30	242	3	
	1991	7	18	123	1	
	1991	7	21	236	2	
	1991	7	22	254	2.584962501	
	1991	7	26	275	3.584962501	
	1991	7	27	253	2	
	1991	8	16	289	1	
	1991	8	24	354	2	
	1991	8	25	294	2.584962501	
	1991	8	26	242	3	
	1991	9	21	155	1	
	1991	9	25	150	1	
	1991	9	26	158	2	
	1991	9	27	162	3.321928095	
	1991	9	30	186	1	
	1992	5	5	123	1	
	1992	5	6	119	2.584962501	
	1992	5	7	92	1	
	1992	5	8	105	1	

1992	5	9	101	2
1992	5	10	93	1
1992	5	15	83	1
1992	5	16	58	1
1992	5	17	52	2.584962501
1992	5	20	75	1
1992	5	21	141	1
1992	5	22	136	3.321928095
1992	5	27	102	1
1992	6	22	104	1
1992	7	4	95	1
1992	7	12	176	1
1992	7	27	81	1
1992	8	2	128	1
1992	8	9	132	1
1992	8	15	108	2
1992	8	19	98	1
1992	8	21	68	1
1992	8	22	63	1
1992	8	25	28	1
1992	8	26	43	1
1992	8	27	48	1
1992	8	28	47	3.584962501
1992	8	29	57	3.807354922
1992	8	30	50	5.169925001
1992	9	1	69	6.129283017
1992	9	2	72	8.6794801
1992	9	3	65	5.807354922
1992	9	4	68	6.807354922
1992	9	5	69	5.392317423
1992	9	6	77	4.459431619
1992	9	7	65	3.584962501
1992	9	8	56	3.584962501
1992	9	9	84	5.459431619
1992	9	12	93	3
1992	9	16	112	2
1992	9	22	97	1
1992	9	27	127	2
1993	5	10	154	1
1993	5	25	68	1
1993	5	26	85	1
1993	6	14	14	1
1993	6	15	14	2.584962501
1993	6	16	18	1
1993	6	17	24	1
1993	6	18	22	3.584962501
1993	6	19	21	4.169925001
1993	6	20	34	5.392317423

1993	6	21	36	5.64385619
1993	6	22	38	5.459431619
1993	6	23	71	5.64385619
1993	6	24	70	4.700439718
1993	6	25	82	3.584962501
1993	6	26	82	4.459431619
1993	6	27	94	4.459431619
1993	6	28	96	4.700439718
1993	6	29	110	5.95419631
1993	6	30	98	5.321928095
1993	7	1	81	5.64385619
1993	7	2	85	5.169925001
1993	7	3	92	4.906890596
1993	7	4	111	4.459431619
1993	7	5	101	5.169925001
1993	7	6	81	5.321928095
1993	7	7	62	4.906890596
1993	7	8	56	5
1993	7	9	53	5.64385619
1993	7	10	43	5.321928095
1993	7	11	49	2.584962501
1993	7	12	49	2.584962501
1993	7	13	71	2.584962501
1993	7	18	111	2.584962501
1993	7	19	114	1
1993	7	20	99	2
1993	7	23	94	2
1993	7	24	92	3.321928095
1993	7	25	85	2.584962501
1993	7	26	76	3
1993	7	27	83	3.584962501
1993	7	28	82	2
1993	7	29	65	4
1993	7	30	62	5.64385619
1993	7	31	64	6.285402219
1993	8	1	56	6.044394119
1993	8	2	59	6.754887502
1993	8	3	53	7.266786541
1993	8	4	52	7.459431619
1993	8	5	40	7.409390936
1993	8	6	73	7.14974712
1993	8	7	64	7.491853096
1993	8	8	70	7.794415866
1993	8	9	87	6.977279923
1993	8	10	96	3.807354922
1993	8	11	108	4.321928095
1993	8	12	86	4.169925001
1993	8	13	89	3.807354922

1993	8	14	58	3.584962501
1993	8	15	43	4
1993	8	16	33	3.584962501
1993	8	17	43	6.209453366
1993	8	18	41	3.321928095
1993	8	19	41	8.022367813
1993	8	20	34	7.189824559
1993	8	21	46	6.754887502
1993	8	22	62	7.62935662
1993	8	23	62	6.781359714
1993	8	24	83	6.781359714
1993	8	25	79	7.700439718
1993	8	26	65	6.357552005
1993	8	27	73	5.584962501
1993	8	28	71	6.129283017
1993	8	29	73	5.754887502
1993	8	30	55	6.554588852
1993	8	31	44	6.321928095
1993	9	1	36	5.169925001
1993	9	2	33	4.807354922
1993	9	3	31	5.087462841
1993	9	4	32	5.857980995
1993	9	5	31	5.321928095
1993	9	6	19	4.700439718
1993	9	7	17	5.087462841
1993	9	8	17	5.523561956
1993	9	9	22	4.169925001
1993	9	10	13	4
1993	9	11	17	4.321928095
1993	9	12	13	4.169925001
1993	9	13	14	3.807354922
1993	9	14	14	3.584962501
1993	9	15	14	4
1993	9	16	32	3.584962501
1993	9	17	29	6.209453366
1993	9	18	26	3.321928095
1994	4	1	29	1
1994	4	14	20	1
1994	4	15	32	1
1994	4	16	20	1
1994	4	17	29	2
1994	4	19	36	3.321928095
1994	4	20	48	4
1994	4	21	53	4
1994	4	22	65	1
1994	4	26	51	1
1994	4	27	53	2
1994	4	29	15	1

1994	5	1	30	1
1994	5	14	52	1
1994	5	15	50	1
1994	5	16	55	1
1994	5	17	65	2
1994	5	19	47	3.807354922
1994	5	20	42	4
1994	5	21	42	4.321928095
1994	5	22	35	3.321928095
1994	5	26	0	1
1994	5	27	0	2
1994	5	29	0	3.321928095
1994	6	2	0	2.584962501
1994	6	11	89	1
1994	6	16	74	1
1994	6	25	40	2
1994	6	26	21	1
1994	6	28	21	1
1994	7	6	39	2.584962501
1994	7	8	87	2.584962501
1994	7	10	87	2.584962501
1994	7	15	62	1
1994	7	25	17	1
1994	8	3	20	1
1994	8	5	27	2
1994	8	12	55	1
1994	8	13	60	2.584962501
1994	8	14	67	2
1994	8	15	64	2
1994	8	16	66	3.584962501
1994	8	17	63	1
1994	8	22	15	2.584962501
1994	8	23	35	1
1994	8	24	18	1
1994	8	25	18	2.584962501
1994	8	26	18	3
1994	8	27	17	2.584962501
1994	8	28	20	2.584962501
1994	8	29	24	3
1994	8	30	34	1
1994	8	31	53	1
1994	9	1	60	3.321928095
1994	9	2	77	3.321928095
1994	9	5	102	2
1994	9	6	90	3.584962501
1994	9	10	49	3
1994	9	11	30	4.321928095
1994	9	12	16	4

1994	9	13	14	3
1994	9	14	14	2.584962501
1994	9	15	13	3.807354922
1994	9	16	13	3.584962501
1994	9	17	19	2.584962501
1994	9	18	35	2
1994	9	20	0	3.807354922
1994	9	21	0	2.584962501
1994	9	22	17	2.584962501
1994	9	23	19	2.584962501
1994	9	24	22	1
1994	9	25	25	2
1994	9	30	27	2.584962501
1995	5	2	0	1
1995	5	16	54	1
1995	5	21	17	2
1995	5	23	0	1
1995	5	25	0	1
1995	5	27	0	2
1995	5	28	12	1
1995	6	3	17	1
1995	6	4	20	1
1995	6	16	0	1
1995	6	24	19	1
1995	6	29	20	3
1995	6	30	33	2.584962501
1995	7	6	38	1
1995	7	7	41	1
1995	7	10	30	1
1995	7	14	18	2
1995	7	21	20	1
1995	7	26	10	1
1995	7	27	11	1
1995	7	28	0	1
1995	7	31	14	2
1995	8	3	22	1
1995	8	7	23	1
1995	8	8	17	3.321928095
1995	8	9	11	4
1995	8	11	14	1
1995	8	12	0	1
1995	8	13	11	1
1995	8	14	0	2
1995	8	16	27	2
1995	8	17	17	1
1995	8	19	0	3
1995	8	20	10	3
1995	8	21	0	1

1995	8	22	10	1
1995	8	23	19	3
1995	8	24	40	2
1995	8	25	38	2.584962501
1995	8	26	52	4
1995	8	27	41	2.584962501
1995	8	29	18	3.584962501
1995	8	30	17	3
1995	8	31	14	3.321928095
1995	9	1	12	3
1995	9	2	11	2
1995	9	3	11	2
1995	9	4	21	1
1995	9	5	21	2
1995	9	7	11	1
1995	9	8	0	1
1995	9	9	0	2
1995	9	12	12	1
1995	9	17	0	2
1995	9	18	0	2
1995	9	20	28	2
1995	9	21	29	2
1995	9	22	40	2.584962501
1996	5	15	15	1
1996	5	19	0	2
1996	5	20	11	2.584962501
1996	5	22	0	1
1996	5	23	0	1
1996	5	25	0	3
1996	5	26	0	2
1996	5	27	0	1
1996	6	14	0	2
1996	6	20	11	1
1996	6	28	25	1
1996	7	1	12	2
1996	7	4	0	3.584962501
1996	7	5	12	2
1996	7	6	0	3.584962501
1996	7	7	20	2
1996	7	8	37	2.584962501
1996	7	9	39	2
1996	7	10	37	4.807354922
1996	7	11	29	4.169925001
1996	7	12	19	3.584962501
1996	7	13	13	4.169925001
1996	7	14	0	2.584962501
1996	7	15	0	3
1996	7	16	0	4.169925001

1996	7	17	0	3.584962501
1996	7	18	0	4.321928095
1996	7	19	0	2.584962501
1996	7	20	0	1
1996	7	21	0	2.584962501
1996	7	23	0	3.321928095
1996	7	24	0	2
1996	7	25	0	3.321928095
1996	7	26	12	2
1996	7	27	13	1
1996	7	28	17	1
1996	7	31	30	1
1996	8	1	26	2.584962501
1996	8	2	31	1
1996	8	3	31	4
1996	8	4	29	2.584962501
1996	8	5	22	3
1996	8	6	14	2
1996	8	7	23	5.247927513
1996	8	8	15	6.700439718
1996	8	9	19	7.14974712
1996	8	10	20	7.169925001
1996	8	11	29	5.247927513
1996	8	12	23	4.700439718
1996	8	13	16	6.727920455
1996	8	14	29	7.442943496
1996	8	15	26	8.087462841
1996	8	16	14	7.894817763
1996	8	17	15	6.357552005
1996	8	18	12	7.727920455
1996	8	19	15	4.807354922
1996	8	20	14	7.459431619
1996	8	21	14	5
1996	8	22	12	4
1996	8	23	14	4.906890596
1996	8	24	22	3.584962501
1996	8	25	14	3.807354922
1996	8	26	18	3
1996	8	27	18	3
1996	8	28	15	3
1996	8	29	15	2
1996	8	30	19	3.584962501
1996	8	31	26	3.807354922
1996	9	1	24	5.523561956
1996	9	2	17	5.754887502
1996	9	3	13	6.087462841
1996	9	4	0	6
1996	9	5	0	5.754887502

1996	9	6	0	5
1996	9	7	22	4.700439718
1996	9	8	0	4.807354922
1996	9	9	0	4.459431619
1996	9	10	0	3.807354922
1996	9	11	0	3.584962501
1996	9	12	15	1
1996	9	13	0	3.584962501
1996	9	14	0	4.700439718
1996	9	15	0	4.584962501
1996	9	16	0	4.807354922
1996	9	17	0	3.321928095
1996	9	18	0	3.321928095
1996	9	19	0	2.584962501
1996	9	20	0	1
1996	9	21	0	2
1996	9	22	0	1
1996	9	26	0	1
1996	9	27	0	1
1996	9	28	0	1
1996	9	30	0	1
1997	4	26	25	1
1997	4	27	16	1
1997	4	28	18	1
1997	4	29	12	1
1997	4	30	0	1
1997	5	7	11	2
1997	5	8	14	1
1997	5	26	48	2
1997	5	28	23	2
1997	5	29	19	1
1997	6	1	33	3
1997	6	2	36	2
1997	6	5	18	3.321928095
1997	6	6	20	2
1997	6	14	31	1
1997	6	16	33	1
1997	6	17	21	1
1997	6	19	16	1
1997	6	21	11	1
1997	6	22	0	1
1997	6	24	15	1
1997	6	27	20	2.584962501
1997	6	29	13	2
1997	7	4	11	1
1997	7	24	36	2.584962501
1997	7	25	48	2
1997	7	26	42	2.584962501

1997	7	27	31	3
1997	7	28	14	5.754887502
1997	7	29	14	4.459431619
1997	7	30	0	3.584962501
1997	7	31	0	6.087462841
1997	8	1	0	5.087462841
1997	8	2	12	5.754887502
1997	8	3	19	4.459431619
1997	8	4	13	4.169925001
1997	8	5	25	2.584962501
1997	8	6	53	3
1997	8	7	60	2.584962501
1997	8	8	66	3
1997	8	11	53	4.807354922
1997	8	12	70	5.247927513
1997	8	13	67	4.169925001
1997	8	14	57	2.584962501
1997	8	15	54	1
1997	8	16	31	2
1997	8	17	26	1
1997	8	27	35	1
1997	8	31	78	3.321928095
1997	9	1	76	2
1997	9	2	69	2.584962501
1997	9	3	56	3
1997	9	4	49	2.584962501
1997	9	5	58	1
1997	9	6	65	2.584962501
1997	9	7	79	1
1997	9	11	100	1
1997	9	12	103	1
1997	9	15	76	1
1997	9	16	71	3.321928095
1997	9	17	57	1
1997	9	18	38	1
1997	9	20	16	1
1997	9	21	30	1
1997	9	22	52	1
1997	9	23	49	3
1997	9	25	57	2
1997	9	26	30	2.584962501
1997	9	27	35	1
1998	4	23	34	1
1998	4	26	17	2
1998	4	27	16	2
1998	4	28	37	1
1998	4	29	48	1
1998	5	2	96	1

1998	5	6	100	2
1998	5	15	105	1
1998	5	16	104	2
1998	5	17	93	1
1998	5	22	28	3.584962501
1998	5	28	43	2.584962501
1998	5	30	53	1
1998	5	31	64	2
1998	6	6	92	1
1998	6	9	104	1
1998	6	11	97	1
1998	6	21	82	3.321928095
1998	6	22	65	3.321928095
1998	6	23	68	4
1998	6	24	58	4.459431619
1998	6	25	96	4.700439718
1998	6	26	106	5.169925001
1998	6	27	128	3.807354922
1998	6	28	138	2.584962501
1998	6	29	140	4.321928095
1998	6	30	129	1
1998	7	1	137	1
1998	7	2	138	3.321928095
1998	7	3	138	1
1998	7	4	145	3.321928095
1998	7	5	137	1
1998	7	6	107	1
1998	7	26	91	2
1998	7	28	123	1
1998	7	29	107	4
1998	7	30	83	3
1998	8	1	84	2
1998	8	2	100	2
1998	8	3	89	4
1998	8	4	87	4.321928095
1998	8	5	92	3.807354922
1998	8	6	129	3
1998	8	7	137	3
1998	8	8	127	3.584962501
1998	8	9	164	3.321928095
1998	8	11	155	4.169925001
1998	8	12	159	2.584962501
1998	8	13	121	4.169925001
1998	8	14	123	5.087462841
1998	8	15	96	5
1998	8	16	123	3.807354922
1998	8	17	114	2.584962501
1998	8	18	109	4.321928095

1998	8	19	133	5.321928095
1998	8	20	120	5.857980995
1998	8	21	117	5.459431619
1998	8	22	106	5.754887502
1998	8	23	110	4.321928095
1998	8	24	104	4.700439718
1998	8	25	96	2.584962501
1998	8	26	114	2.584962501
1998	8	27	131	3.584962501
1998	8	28	126	2
1998	8	29	134	1
1998	9	4	97	2
1998	9	5	114	3.807354922
1998	9	6	159	4.169925001
1998	9	7	165	3.807354922
1998	9	8	178	2
1998	9	9	169	4.459431619
1998	9	10	159	4.169925001
1998	9	11	136	4.584962501
1998	9	12	131	5.459431619
1998	9	13	135	5.754887502
1998	9	14	111	5.087462841
1998	9	15	85	1
1998	9	16	80	3.321928095
1998	9	17	105	4.321928095
1998	9	18	99	3.584962501
1998	9	19	132	3.321928095
1998	9	20	162	3.584962501
1998	9	21	178	4.459431619
1998	9	22	196	3.584962501
1998	9	23	192	4.584962501
1998	9	24	166	3.584962501
1998	9	25	149	3
1998	9	26	116	2
1998	9	29	85	1
1998	9	30	58	1
1999	4	14	109	1
1999	4	25	66	1
1999	4	29	90	2
1999	4	30	97	1
1999	5	3	112	1
1999	5	4	118	2
1999	5	5	124	1
1999	5	6	146	3.321928095
1999	5	7	200	3.584962501
1999	5	8	214	3.321928095
1999	5	9	210	2.584962501
1999	5	10	191	3

1999	5	11	188	1
1999	5	13	142	1
1999	5	14	132	2
1999	5	15	148	2
1999	5	16	148	5.392317423
1999	5	17	139	1
1999	5	18	131	3.321928095
1999	5	19	132	2
1999	5	20	135	3.321928095
1999	5	22	138	2
1999	5	23	121	2.584962501
1999	5	24	120	2
1999	5	26	160	1
1999	5	29	162	1
1999	6	12	230	2.584962501
1999	6	14	221	2
1999	6	16	181	5.459431619
1999	6	17	146	5.321928095
1999	6	18	120	2.584962501
1999	6	19	119	5.64385619
1999	6	20	93	5
1999	6	22	160	5.95419631
1999	6	23	217	4.169925001
1999	6	24	293	3
1999	6	25	292	6.087462841
1999	6	26	274	5
1999	6	27	259	2.584962501
1999	6	28	254	6.087462841
1999	6	29	241	5.584962501
1999	6	30	223	5.807354922
1999	7	1	215	6.754887502
1999	7	2	223	4
1999	7	3	217	5.459431619
1999	7	4	205	5.807354922
1999	7	5	199	5.807354922
1999	7	6	186	6.044394119
1999	7	7	179	3.321928095
1999	7	8	173	4.700439718
1999	7	9	176	5.321928095
1999	7	10	171	3.807354922
1999	7	11	176	5.754887502
1999	7	12	199	4.459431619
1999	7	13	157	2
1999	7	14	128	3
1999	7	15	122	3
1999	7	17	121	5.392317423
1999	7	29	246	2
1999	7	30	252	4.906890596

1999	7	31	223	3
1999	8	1	252	4.169925001
1999	8	2	250	4.459431619
1999	8	3	229	4
1999	8	4	193	5.523561956
1999	8	5	193	5.392317423
1999	8	6	167	4.584962501
1999	8	7	152	2
1999	8	8	149	7.930737338
1999	8	9	115	9.392317423
1999	8	10	82	8.189824559
1999	8	11	79	8.238404739
1999	8	12	91	5.754887502
1999	8	13	102	5.754887502
1999	8	14	87	3
1999	8	15	74	3
1999	8	16	67	5.95419631
1999	8	17	55	7.247927513
1999	8	18	58	6.209453366
1999	8	19	64	5.95419631
1999	8	20	73	6.357552005
1999	8	21	88	4.459431619
1999	8	22	103	6.700439718
1999	8	23	115	6.169925001
1999	8	24	131	6.614709844
1999	8	25	196	3.321928095
1999	8	26	206	3
1999	8	27	194	2
1999	8	28	223	2
1999	8	29	231	3.584962501
1999	8	30	228	4
1999	8	31	165	3
1999	9	1	140	4.700439718
1999	9	2	122	2
1999	9	3	115	1
1999	9	4	103	4.906890596
1999	9	5	106	4.807354922
1999	9	6	86	1
1999	9	7	103	3.321928095
1999	9	8	113	4.584962501
1999	9	9	109	4.906890596
1999	9	10	110	4.584962501
1999	9	11	113	5.523561956
1999	9	12	126	5.247927513
1999	9	13	152	2
1999	9	19	128	2
1999	9	20	97	2
1999	9	21	65	2

1999	9	22	89	2
1999	9	24	61	3
1999	9	25	43	3.321928095
1999	9	26	46	3.807354922
1999	9	27	52	2.584962501
1999	9	28	68	2
1999	10	3	98	1
1999	10	6	197	1
2000	4	16	150	1
2000	4	21	195	1
2000	4	23	259	2
2000	4	25	230	2.584962501
2000	4	26	208	1
2000	4	30	153	1
2000	5	2	109	2.584962501
2000	5	3	104	2.584962501
2000	5	4	97	2
2000	5	8	87	3
2000	5	9	135	3.321928095
2000	5	10	164	1
2000	5	11	181	2.584962501
2000	5	12	181	1
2000	5	13	220	1
2000	5	14	263	1
2000	5	15	280	1
2000	5	22	195	4.584962501
2000	5	23	180	2.584962501
2000	5	24	183	2
2000	5	25	157	2.584962501
2000	5	26	160	3.321928095
2000	5	27	145	3.321928095
2000	5	28	169	1
2000	5	29	160	2.584962501
2000	5	30	127	1
2000	5	31	91	4.807354922
2000	6	1	128	3
2000	6	2	119	2
2000	6	3	113	1
2000	6	4	152	2.584962501
2000	6	5	143	2.584962501
2000	6	6	149	4.459431619
2000	6	7	158	2
2000	6	8	181	4.169925001
2000	6	9	184	5
2000	6	10	179	4.459431619
2000	6	11	227	4
2000	6	12	221	2
2000	6	13	235	5

2000	6	14	257	3.584962501
2000	6	15	238	4.807354922
2000	6	16	214	5.523561956
2000	6	17	209	5.392317423
2000	6	18	221	6.523561956
2000	6	19	218	8.375039431
2000	6	21	221	3
2000	6	22	191	6.807354922
2000	6	23	187	6.523561956
2000	6	24	179	5.807354922
2000	6	25	167	4.169925001
2000	6	26	194	6.832890014
2000	6	27	208	9.434628228
2000	6	28	173	7.189824559
2000	6	29	164	5.700439718
2000	6	30	172	6.087462841
2000	7	1	208	5.321928095
2000	7	2	202	4.169925001
2000	7	3	178	4
2000	7	4	164	6.459431619
2000	7	5	182	5.169925001
2000	7	6	221	5.64385619
2000	7	7	254	4.169925001
2000	7	8	254	5.247927513
2000	7	9	257	3.321928095
2000	7	10	309	4
2000	7	12	267	4.459431619
2000	7	13	279	2.584962501
2000	7	15	213	1
2000	7	16	283	2
2000	7	17	322	2
2000	7	18	327	3
2000	7	19	353	5
2000	7	20	346	6.584962501
2000	7	21	332	7.965784285
2000	7	22	310	8.169925001
2000	7	23	286	7.87036472
2000	7	24	246	7.129283017
2000	7	25	254	6.977279923
2000	7	26	191	8.721099189
2000	7	27	181	8.531381461
2000	7	28	172	4
2000	7	29	162	9.54689446
2000	7	30	161	9.87036472
2000	7	31	134	8.539158811
2000	8	1	147	8.686500527
2000	8	2	152	7.539158811
2000	8	3	148	5.807354922

2000	8	4	152	7.918863237
2000	8	5	199	7.882643049
2000	8	6	198	7.794415866
2000	8	7	227	7.22881869
2000	8	8	194	9.103287808
2000	8	9	177	7.882643049
2000	8	10	213	6.832890014
2000	8	11	228	6.491853096
2000	8	12	235	7.339850003
2000	8	13	243	8.46760555
2000	8	14	282	5.807354922
2000	8	15	253	5.169925001
2000	8	16	246	1
2000	8	17	210	5.321928095
2000	8	18	194	2
2000	8	19	184	4.321928095
2000	8	20	147	2.584962501
2000	8	21	106	5.95419631
2000	8	22	93	5.459431619
2000	8	23	93	5.392317423
2000	8	24	106	4
2000	8	25	112	4
2000	8	26	109	5.906890596
2000	8	27	156	5
2000	8	28	183	4.584962501
2000	8	29	191	2
2000	8	30	199	3.321928095
2000	8	31	217	1
2000	9	1	202	2.584962501
2000	9	2	168	4.807354922
2000	9	3	182	5.247927513
2000	9	4	191	4.700439718
2000	9	5	162	6.392317423
2000	9	6	162	4.459431619
2000	9	7	156	5.857980995
2000	9	8	121	4.584962501
2000	9	9	90	1
2000	9	10	60	3.321928095
2000	9	11	37	2
2000	9	12	50	4.906890596
2000	9	13	78	1
2000	9	14	85	2.584962501
2000	9	15	109	1
2000	9	16	121	4.584962501
2000	9	17	154	4.700439718
2000	9	18	159	4.321928095
2000	9	19	172	4.169925001
2000	9	20	176	4.807354922

2000	9	21	195	3.807354922
2000	9	22	202	5.459431619
2000	9	23	227	3
2000	9	24	232	1
2000	9	26	229	1
2001	5	1	157	1
2001	5	7	116	1
2001	5	8	81	1
2001	5	9	93	2
2001	5	10	88	2
2001	5	11	118	1
2001	5	12	124	2.584962501
2001	5	13	125	3.807354922
2001	5	15	141	2.584962501
2001	5	16	146	1
2001	5	17	140	1
2001	5	21	140	3.321928095
2001	5	24	174	2
2001	5	25	165	1
2001	5	27	182	2.584962501
2001	5	28	151	1
2001	5	30	110	2.584962501
2001	5	31	101	1
2001	6	7	195	1
2001	6	10	241	2
2001	6	11	262	3
2001	6	12	259	3.807354922
2001	6	13	242	4
2001	6	14	273	4.906890596
2001	6	15	282	3.584962501
2001	6	16	289	1
2001	6	17	270	3.807354922
2001	6	18	232	5.392317423
2001	6	19	214	5.392317423
2001	6	20	206	4.807354922
2001	6	21	218	5.169925001
2001	6	22	229	3
2001	6	23	235	3
2001	6	24	220	2.584962501
2001	6	25	198	2
2001	6	26	173	3.321928095
2001	6	27	162	3
2001	6	28	135	2
2001	6	29	112	2
2001	6	30	98	2.584962501
2001	7	1	111	2
2001	7	2	125	5
2001	7	3	120	4.459431619

2001	7	4	107	3.321928095
2001	7	5	93	2
2001	7	6	68	1
2001	7	7	71	2
2001	7	8	81	1
2001	7	9	107	2.584962501
2001	7	10	105	1
2001	7	11	104	2
2001	7	12	135	2
2001	7	13	167	1
2001	7	14	149	2.584962501
2001	7	15	153	3
2001	7	16	170	2
2001	7	17	185	3.807354922
2001	7	18	191	5.087462841
2001	7	19	183	3.584962501
2001	7	20	177	5.169925001
2001	7	21	144	6.754887502
2001	7	22	150	6.977279923
2001	7	23	152	5.321928095
2001	7	24	135	6.554588852
2001	7	25	119	4.459431619
2001	7	26	92	4.807354922
2001	7	27	90	6.247927513
2001	7	28	95	5.584962501
2001	7	29	69	4.584962501
2001	7	30	86	5.087462841
2001	7	31	78	4.584962501
2001	8	1	94	5.64385619
2001	8	2	123	3.584962501
2001	8	3	141	3
2001	8	4	175	3
2001	8	5	198	4
2001	8	6	182	6.614709844
2001	8	7	179	5.459431619
2001	8	8	178	6.129283017
2001	8	9	158	5.64385619
2001	8	10	150	6.357552005
2001	8	11	170	5.807354922
2001	8	12	170	7.62935662
2001	8	13	138	8
2001	8	14	141	7.87036472
2001	8	15	161	4.700439718
2001	8	16	193	5.459431619
2001	8	17	178	5.392317423
2001	8	18	161	5.087462841
2001	8	19	152	5.807354922
2001	8	20	153	4.321928095

2001	8	21	167	4.700439718
2001	8	22	170	3.807354922
2001	8	23	181	3.584962501
2001	8	24	176	3.584962501
2001	8	25	140	3.807354922
2001	8	26	153	2.584962501
2001	8	27	170	3
2001	8	28	184	4.169925001
2001	8	29	146	2.584962501
2001	8	30	150	2.584962501
2001	8	31	175	3
2001	9	1	163	2
2001	9	2	168	2
2001	9	3	190	3
2001	9	4	171	3
2001	9	5	190	2.584962501
2001	9	6	223	4.169925001
2001	9	7	262	3
2001	9	8	288	4
2001	9	9	262	4
2001	9	10	237	4.169925001
2001	9	11	199	4.169925001
2001	9	12	235	3.584962501
2001	9	13	237	4.584962501
2001	9	14	234	1
2001	9	15	205	2
2001	9	16	191	3
2001	9	17	177	3.584962501
2001	9	18	215	2.584962501
2001	9	19	226	2.584962501
2001	9	20	289	1
2001	9	21	273	2.584962501
2001	9	22	259	3
2001	9	23	294	2.584962501
2001	9	24	316	2
2001	9	25	305	2
2001	9	26	277	1
2001	9	27	278	1
2001	9	28	269	2.584962501
2001	9	30	261	2.584962501
2001	10	1	260	3
2001	10	2	223	2
2001	10	3	209	1
2001	10	5	176	1
2001	10	6	161	2.584962501
2001	10	7	159	1
2001	10	8	119	3
2001	10	9	122	1

2001	10	10	151	1
2002	4	15	214	1
2002	4	21	147	2
2002	4	22	144	2
2002	4	23	177	1
2002	4	24	232	1
2002	4	25	228	1
2002	5	1	158	1
2002	5	2	177	2
2002	5	3	231	2
2002	5	4	258	1
2002	5	5	267	1
2002	5	6	231	2.584962501
2002	5	8	220	3
2002	5	9	196	2.584962501
2002	5	10	207	3.321928095
2002	5	11	214	2
2002	5	12	202	2
2002	5	13	161	4.169925001
2002	5	14	118	1
2002	5	15	115	1
2002	5	17	134	2
2002	5	21	188	2
2002	5	22	213	1
2002	5	29	177	1
2002	5	30	160	1
2002	6	7	197	2
2002	6	8	185	2.584962501
2002	6	9	165	2
2002	6	10	128	1
2002	6	11	99	4.321928095
2002	6	12	109	3.584962501
2002	6	13	80	4.169925001
2002	6	14	107	3
2002	6	15	102	4.584962501
2002	6	16	117	4.321928095
2002	6	17	88	4.807354922
2002	6	18	127	4.700439718
2002	6	19	115	5.321928095
2002	6	20	108	5.392317423
2002	6	21	83	5
2002	6	22	83	4.584962501
2002	6	23	95	7.087462841
2002	6	24	108	5.906890596
2002	6	25	111	5.169925001
2002	6	26	108	5.247927513
2002	6	27	96	6.584962501
2002	6	28	88	8.022367813

2002	6	29	96	7.672425342
2002	6	30	105	5.247927513
2002	7	1	94	4.584962501
2002	7	2	99	6.459431619
2002	7	3	129	4.906890596
2002	7	4	133	4.906890596
2002	7	5	142	2
2002	7	6	121	4.459431619
2002	7	7	107	3.584962501
2002	7	8	102	2.584962501
2002	7	9	103	2.584962501
2002	7	10	94	1
2002	7	11	99	1
2002	7	12	84	3.584962501
2002	7	13	116	3.584962501
2002	7	15	155	2
2002	7	16	160	3
2002	7	17	147	1
2002	7	18	149	1
2002	7	21	124	2
2002	7	22	147	2.584962501
2002	7	23	196	1
2002	7	24	209	2
2002	7	25	215	1
2002	7	26	265	3
2002	7	27	294	2
2002	7	28	310	5
2002	7	29	293	5.321928095
2002	7	30	281	5.247927513
2002	7	31	239	4
2002	8	1	207	4.459431619
2002	8	2	199	6.95419631
2002	8	3	183	8.199672345
2002	8	4	143	5.321928095
2002	8	5	127	5.64385619
2002	8	6	131	4.321928095
2002	8	7	133	6.459431619
2002	8	8	115	6.321928095
2002	8	9	110	6.392317423
2002	8	10	110	7.569855608
2002	8	11	149	6.882643049
2002	8	12	177	6.209453366
2002	8	13	202	7.06608919
2002	8	14	267	4.584962501
2002	8	15	279	5.459431619
2002	8	16	263	5.321928095
2002	8	17	281	4.807354922
2002	8	18	270	5.754887502

2002	8	19	248	5.247927513
2002	8	20	211	4.584962501
2002	8	21	192	5.087462841
2002	8	22	172	4.321928095
2002	8	23	186	3.807354922
2002	8	24	149	5.392317423
2002	8	25	148	5.392317423
2002	8	26	119	5.247927513
2002	8	27	121	4
2002	8	28	122	4.459431619
2002	8	29	124	6.087462841
2002	8	30	146	5.857980995
2002	8	31	160	5.321928095
2002	9	1	206	5.64385619
2002	9	2	233	4.584962501
2002	9	3	252	4.807354922
2002	9	4	247	3.807354922
2002	9	5	226	4.321928095
2002	9	6	202	5
2002	9	7	206	3.807354922
2002	9	8	213	3.807354922
2002	9	9	199	4.459431619
2002	9	10	202	5.700439718
2002	9	11	187	5.584962501
2002	9	12	187	4.584962501
2002	9	13	187	4.459431619
2002	9	14	149	2
2002	9	15	166	2.584962501
2002	9	16	170	3.321928095
2002	9	17	199	3.584962501
2002	9	18	207	1
2002	9	19	192	3.321928095
2002	9	20	195	1
2002	9	21	182	2.584962501
2002	9	22	185	3
2002	9	23	192	1
2002	9	24	177	3.321928095
2002	9	25	190	1
2002	9	26	154	1
2002	9	27	154	2
2002	9	28	137	1
2002	9	29	130	2.584962501
2002	9	30	110	2.584962501
2002	10	1	90	2
2002	10	2	109	3.321928095
2002	10	3	104	3
2002	10	4	93	2.584962501
2002	10	5	118	2

2002	10	6	126	2
2002	10	7	123	1
2002	10	8	157	2
2002	10	9	164	1
2002	10	10	200	2
2002	10	11	188	1
2002	10	12	189	1
2003	4	30	160	1
2003	5	2	137	1
2003	5	3	151	1
2003	5	4	153	1
2003	5	7	103	2
2003	5	12	60	2.584962501
2003	5	15	79	1
2003	5	16	81	1
2003	5	17	62	1
2003	5	19	76	1
2003	5	20	97	2
2003	5	21	79	2.584962501
2003	5	22	103	2
2003	5	23	91	2.584962501
2003	5	24	59	3.807354922
2003	5	25	62	2
2003	5	26	83	1
2003	5	27	91	1
2003	5	28	99	2
2003	5	29	89	2
2003	5	30	70	2
2003	6	1	64	2
2003	6	2	58	2
2003	6	3	61	1
2003	6	4	72	3
2003	6	5	91	1
2003	6	14	124	1
2003	6	15	97	2
2003	6	16	87	2
2003	6	17	86	3.321928095
2003	6	18	104	3.807354922
2003	6	19	117	5.857980995
2003	6	20	114	3
2003	6	21	95	2
2003	6	22	94	3
2003	6	23	101	1
2003	6	24	104	3
2003	6	25	117	3.807354922
2003	6	26	126	5.392317423
2003	6	27	143	1
2003	6	28	143	3.321928095

2003	6	29	144	3.321928095
2003	6	30	141	2.584962501
2003	7	1	154	3.584962501
2003	7	2	149	6.426264755
2003	7	3	123	5.392317423
2003	7	4	103	4.807354922
2003	7	5	86	7.129283017
2003	7	6	97	5.087462841
2003	7	7	131	5
2003	7	8	137	3.807354922
2003	7	9	139	3.807354922
2003	7	10	114	4.169925001
2003	7	11	94	1
2003	7	12	105	2
2003	7	17	173	2
2003	7	18	186	2.584962501
2003	7	19	197	3
2003	7	20	248	2.584962501
2003	7	21	225	2
2003	7	22	190	2.584962501
2003	7	23	154	2
2003	7	24	120	2.584962501
2003	7	25	72	3.584962501
2003	7	26	43	3.807354922
2003	7	27	51	3.321928095
2003	7	28	77	4.906890596
2003	7	29	66	4.700439718
2003	7	30	59	4.584962501
2003	7	31	65	5.906890596
2003	8	1	78	4.906890596
2003	8	2	89	5
2003	8	3	121	5.087462841
2003	8	4	116	5.087462841
2003	8	5	138	5.700439718
2003	8	6	132	5.459431619
2003	8	7	124	4.807354922
2003	8	8	109	4.584962501
2003	8	9	111	5.321928095
2003	8	10	114	5.807354922
2003	8	11	114	6
2003	8	12	113	6.285402219
2003	8	13	111	4.321928095
2003	8	14	100	5.321928095
2003	8	15	106	5.169925001
2003	8	16	116	5.321928095
2003	8	17	117	4.807354922
2003	8	18	106	6.209453366
2003	8	19	92	4.807354922

2003	8	20	98	5.247927513
2003	8	21	92	4.700439718
2003	8	22	109	4.321928095
2003	8	23	121	5.087462841
2003	8	24	130	5.169925001
2003	8	25	130	5.64385619
2003	8	26	141	5.392317423
2003	8	27	143	5.64385619
2003	8	28	151	5.754887502
2003	8	29	135	6.247927513
2003	8	30	117	6.247927513
2003	8	31	103	5.857980995
2003	9	1	74	5
2003	9	2	74	4.584962501
2003	9	3	76	4.906890596
2003	9	4	81	5.169925001
2003	9	5	63	4.321928095
2003	9	6	60	4.807354922
2003	9	7	48	5.087462841
2003	9	8	40	4.169925001
2003	9	9	27	5.087462841
2003	9	10	40	5.906890596
2003	9	11	55	2.584962501
2003	9	12	47	2.584962501
2003	9	13	48	3
2003	9	14	53	3
2003	9	18	94	3.321928095
2003	9	19	84	2.584962501
2003	9	21	81	1
2003	9	22	92	3.321928095
2003	9	23	105	3.584962501
2003	9	24	103	3
2003	9	25	108	4.459431619
2003	9	26	124	4.459431619
2003	9	27	127	3.584962501
2003	9	28	115	4
2003	9	29	119	4.169925001
2003	9	30	106	4.807354922
2003	10	1	114	2.584962501
2003	10	2	102	3
2003	10	3	93	2
2003	10	4	73	3
2003	10	5	75	3
2003	10	6	61	2
2003	10	7	61	2
2003	10	8	64	3
2003	10	9	70	3.321928095
2003	10	10	67	2.584962501

2003	10	11	66	2
2003	10	12	37	1
2003	10	13	19	2.584962501
2003	10	14	19	2
2003	10	15	19	1
2004	4	8	41	1
2004	4	16	47	1
2004	4	17	75	1
2004	4	18	87	2
2004	4	19	95	2.584962501
2004	4	20	89	2.584962501
2004	4	21	89	1
2004	4	25	47	1
2004	4	26	51	3.321928095
2004	4	27	39	3
2004	4	28	35	4.321928095
2004	4	30	51	3
2004	5	1	77	2
2004	5	2	49	1
2004	5	3	56	1
2004	5	4	58	1
2004	5	5	51	2
2004	5	6	35	3.807354922
2004	5	7	28	4.807354922
2004	5	8	30	4.169925001
2004	5	9	42	4.906890596
2004	5	10	51	3.584962501
2004	5	11	44	3.807354922
2004	5	12	70	5.321928095
2004	5	13	65	5.321928095
2004	5	14	95	5.523561956
2004	5	16	128	3.321928095
2004	5	17	121	3.584962501
2004	5	18	105	4
2004	5	19	102	3.321928095
2004	5	20	104	5.169925001
2004	5	21	91	2.584962501
2004	5	22	83	4.584962501
2004	5	23	104	1
2004	5	24	109	1
2004	5	25	97	3.807354922
2004	5	26	76	4.321928095
2004	5	27	56	4.459431619
2004	5	28	53	1
2004	5	29	54	2.584962501
2004	5	30	61	2
2004	5	31	60	3.807354922
2004	6	1	54	3.321928095

2004	6	5	55	3.584962501
2004	6	7	57	2
2004	6	8	51	3
2004	6	9	52	2
2004	6	11	37	3
2004	6	13	43	2
2004	6	14	54	3.584962501
2004	6	15	68	3
2004	6	16	98	3
2004	6	17	95	6.169925001
2004	6	18	103	7.768184325
2004	6	19	106	7.794415866
2004	6	20	132	8.330916878
2004	6	21	134	9.06608919
2004	6	22	117	8.451211112
2004	6	23	94	7.50779464
2004	6	24	80	6.614709844
2004	6	25	57	4.807354922
2004	6	26	52	5.906890596
2004	6	27	48	3.807354922
2004	6	28	46	3.321928095
2004	6	29	40	4.807354922
2004	6	30	34	4.584962501
2004	7	1	28	4.807354922
2004	7	2	33	4.807354922
2004	7	3	33	4.584962501
2004	7	4	31	5.584962501
2004	7	5	26	4.906890596
2004	7	6	28	4.459431619
2004	7	7	15	4.459431619
2004	7	10	62	2.584962501
2004	7	11	77	2.584962501
2004	7	12	89	2.584962501
2004	7	13	144	5.754887502
2004	7	14	148	5.857980995
2004	7	15	135	7.247927513
2004	7	16	107	5.321928095
2004	7	17	130	4.700439718
2004	7	18	153	4.807354922
2004	7	19	164	3.807354922
2004	7	20	149	4.459431619
2004	7	21	144	3.584962501
2004	7	22	138	3
2004	7	23	121	4.321928095
2004	7	24	113	5.247927513
2004	7	25	94	5.906890596
2004	7	26	105	6.321928095
2004	7	27	90	5.906890596

2004	7	28	64	5.321928095
2004	7	29	39	5.321928095
2004	7	30	39	4.584962501
2004	7	31	38	4.584962501
2004	8	1	39	5.95419631
2004	8	2	48	5.247927513
2004	8	3	51	5.169925001
2004	8	4	56	5.321928095
2004	8	5	36	5.807354922
2004	8	6	56	3
2004	8	7	75	8.417852515
2004	8	8	67	7.285402219
2004	8	9	85	4.169925001
2004	8	10	99	1
2004	8	11	107	7.303780748
2004	8	12	116	2.584962501
2004	8	13	130	6.321928095
2004	8	14	116	5.807354922
2004	8	15	104	7.599912842
2004	8	16	92	7.754887502
2004	8	17	75	7.569855608
2004	8	18	70	5.807354922
2004	8	19	61	6.584962501
2004	8	21	97	4.700439718
2004	8	22	113	3.584962501
2004	8	23	96	4.700439718
2004	8	24	65	3
2004	8	25	53	2.584962501
2004	8	26	41	3.584962501
2004	8	27	36	4.700439718
2004	8	28	34	3.321928095
2004	8	29	27	3.321928095
2004	8	30	17	3.807354922
2004	9	1	14	5.64385619
2004	9	2	14	1
2004	9	3	32	1
2004	9	4	35	4.321928095
2004	9	5	65	5
2004	9	6	56	3.807354922
2004	9	7	67	3.584962501
2004	9	8	83	3
2004	9	9	90	4.584962501
2004	9	10	77	2
2004	9	11	74	5.754887502
2004	9	12	76	5.087462841
2004	9	13	69	5.247927513
2004	9	14	56	3.584962501
2004	9	15	69	6.129283017

2004	9	16	67	3.807354922
2004	9	17	63	7.087462841
2004	9	18	58	3.321928095
2004	9	19	60	2.584962501
2004	9	20	47	3.584962501
2004	9	24	18	1
2004	9	27	26	1
2004	10	1	28	1
2004	10	5	32	1
2005	4	21	26	1
2005	4	28	48	3.584962501
2005	4	30	54	3
2005	5	1	61	1
2005	5	2	61	4.321928095
2005	5	3	68	4.584962501
2005	5	4	62	5
2005	5	5	58	2
2005	5	6	65	4.459431619
2005	5	7	54	2
2005	5	8	67	4.807354922
2005	5	9	91	5.087462841
2005	5	10	111	5
2005	5	13	90	3
2005	5	16	62	1
2005	5	19	33	2.584962501
2005	5	24	36	1
2005	5	27	64	1
2005	5	29	62	1
2005	5	31	84	2.584962501
2005	6	3	48	2
2005	6	4	71	2
2005	6	7	105	2
2005	6	8	98	3
2005	6	9	97	1
2005	6	10	101	4
2005	6	11	79	3.807354922
2005	6	13	45	1
2005	6	17	69	1
2005	6	18	46	3.584962501
2005	6	19	42	2
2005	6	20	45	2
2005	6	22	39	1
2005	6	23	16	2.584962501
2005	6	24	16	3.807354922
2005	6	25	17	5.169925001
2005	6	26	0	5.95419631
2005	6	27	12	7.022367813
2005	6	28	13	7.285402219

2005	6	29	40	7.658211483
2005	7	1	122	5.321928095
2005	7	2	148	6.584962501
2005	7	3	157	5.906890596
2005	7	4	167	4.584962501
2005	7	5	160	3.321928095
2005	7	6	131	4
2005	7	7	117	3.584962501
2005	7	8	100	3
2005	7	9	89	4.459431619
2005	7	10	65	4.321928095
2005	7	11	64	4
2005	7	12	59	3
2005	7	13	64	4.700439718
2005	7	14	47	3
2005	7	15	33	3.807354922
2005	7	16	17	1
2005	7	17	12	2.584962501
2005	7	19	14	1
2005	7	20	0	1
2005	7	21	0	1
2005	7	22	12	2
2005	7	23	25	1
2005	7	24	17	3.321928095
2005	7	25	19	2
2005	7	27	17	5.857980995
2005	7	28	28	3.807354922
2005	7	29	53	3.584962501
2005	7	30	67	1
2005	8	2	115	5
2005	8	3	80	4.807354922
2005	8	4	66	3.584962501
2005	8	5	66	5.321928095
2005	8	6	55	6.357552005
2005	8	7	65	5.64385619
2005	8	8	61	5.087462841
2005	8	9	57	5.857980995
2005	8	10	27	6.64385619
2005	8	11	37	6.64385619
2005	8	12	45	6.087462841
2005	8	13	37	5.584962501
2005	8	14	43	3
2005	8	15	45	6.087462841
2005	8	16	40	6.169925001
2005	8	17	33	5.857980995
2005	8	18	32	5.087462841
2005	8	19	73	4.906890596
2005	8	20	80	4.584962501

2005	8	21	65	7.392317423
2005	8	22	63	5.523561956
2005	8	23	60	5.523561956
2005	8	24	70	3.584962501
2005	8	25	68	4.459431619
2005	8	26	61	5.247927513
2005	8	27	70	3.807354922
2005	8	28	76	4.807354922
2005	8	29	71	5.321928095
2005	8	30	61	5.807354922
2005	8	31	48	4.807354922
2005	9	1	29	6.459431619
2005	9	2	24	6.044394119
2005	9	3	15	5.95419631
2005	9	4	15	5.321928095
2005	9	5	14	4.584962501
2005	9	6	14	4
2005	9	7	24	3.584962501
2005	9	8	34	3
2005	9	9	48	3.321928095
2005	9	10	59	3.321928095
2005	9	11	58	5.247927513
2005	9	12	63	4.807354922
2005	9	13	85	5.584962501
2005	9	14	70	4.906890596
2005	9	15	66	5.392317423
2005	9	16	56	6.087462841
2005	9	17	59	6.357552005
2005	9	18	56	2
2005	9	19	44	3.321928095
2005	9	20	24	3
2005	9	21	22	2.584962501
2005	9	22	24	1
2005	9	24	29	1
2005	10	4	21	1
2005	10	8	18	1
2006	4	28	62	1
2006	4	29	53	3
2006	4	30	62	3.321928095
2006	5	1	55	3
2006	5	2	58	4.169925001
2006	5	3	57	3.584962501
2006	5	4	60	2.584962501
2006	5	5	53	3.807354922
2006	5	6	45	4.459431619
2006	5	7	53	4.700439718
2006	5	8	52	3.584962501
2006	5	9	47	3.321928095

2006	5	10	32	4.700439718
2006	5	11	27	1
2006	5	12	12	2
2006	5	13	12	1
2006	5	14	0	3.807354922
2006	5	15	0	4
2006	5	16	0	2
2006	5	17	0	4
2006	5	18	13	4.459431619
2006	5	19	13	4.807354922
2006	5	20	32	4.584962501
2006	5	21	33	5.700439718
2006	5	22	47	4.807354922
2006	5	23	50	4.321928095
2006	5	24	43	3.584962501
2006	5	25	42	3.807354922
2006	5	26	47	3.584962501
2006	5	27	53	5.087462841
2006	5	28	62	3.807354922
2006	5	29	58	4.584962501
2006	5	30	53	3.321928095
2006	5	31	45	3.807354922
2006	6	1	14	5
2006	6	2	0	1
2006	6	3	0	2.584962501
2006	6	4	19	3
2006	6	6	32	1
2006	6	7	58	1
2006	6	8	58	1
2006	6	9	47	2.584962501
2006	6	11	37	2.584962501
2006	6	12	30	1
2006	6	13	28	3.807354922
2006	6	14	14	4.321928095
2006	6	15	14	4.459431619
2006	6	16	21	2.584962501
2006	6	17	23	4
2006	6	18	18	3.321928095
2006	6	19	21	4.169925001
2006	6	20	21	2
2006	6	21	18	4.584962501
2006	6	22	0	7.266786541
2006	6	23	0	4.700439718
2006	6	24	0	7.658211483
2006	6	25	14	8.357552005
2006	6	26	18	8.011227255
2006	6	27	23	8.636624621
2006	6	28	42	8.965784285

2006	6	29	46	5.754887502
2006	6	30	44	6.491853096
2006	7	1	38	7.22881869
2006	7	2	31	4.321928095
2006	7	3	35	3.584962501
2006	7	4	36	4.459431619
2006	7	5	35	3
2006	7	6	36	6.087462841
2006	7	7	36	4.700439718
2006	7	8	35	4.459431619
2006	7	9	31	4
2006	7	10	15	3.807354922
2006	7	11	15	3.321928095
2006	7	14	15	2
2006	7	15	16	2.584962501
2006	7	16	20	2
2006	7	18	22	4
2006	7	20	15	1
2006	7	21	0	1
2006	7	22	15	2
2006	7	24	20	2
2006	7	25	18	3.807354922
2006	7	26	18	5.700439718
2006	7	27	18	6.807354922
2006	7	28	16	6.781359714
2006	7	29	16	7.189824559
2006	7	30	31	7.189824559
2006	7	31	27	6.64385619
2006	8	1	11	8.794415866
2006	8	2	11	6.64385619
2006	8	3	15	8.14974712
2006	8	4	0	9.451211112
2006	8	5	0	7.50779464
2006	8	6	0	7.189824559
2006	8	7	0	7.044394119
2006	8	8	11	5.857980995
2006	8	9	26	5.95419631
2006	8	10	27	5.906890596
2006	8	11	26	7.169925001
2006	8	12	24	8.636624621
2006	8	13	26	8.820178962
2006	8	14	31	9
2006	8	15	31	8.95419631
2006	8	16	31	9.169925001
2006	8	17	27	9.276124405
2006	8	18	31	8.924812504
2006	8	19	24	8.971543554
2006	8	20	16	9.21916852

2006	8	21	26	9.30833903
2006	8	22	15	9.027905997
2006	8	23	19	8.209453366
2006	8	24	19	9.312882955
2006	8	25	24	6.930737338
2006	8	26	23	6.95419631
2006	8	27	34	4.700439718
2006	8	28	35	5.906890596
2006	8	29	27	7.06608919
2006	8	30	19	4.807354922
2006	8	31	35	4.584962501
2006	9	1	35	5.700439718
2006	9	2	16	4.584962501
2006	9	3	0	3.807354922
2006	9	4	0	4.169925001
2006	9	5	26	3.584962501
2006	9	6	39	3.321928095
2006	9	7	48	4.584962501
2006	9	8	48	5.169925001
2006	9	9	49	3.807354922
2006	9	10	49	5.169925001
2006	9	11	44	4
2006	9	12	31	4.169925001
2006	9	13	30	4.321928095
2006	9	14	15	5.857980995
2006	9	15	15	5.321928095
2006	9	16	13	4.584962501
2006	9	17	25	4.169925001
2006	9	18	12	4.807354922
2006	9	19	13	6.857980995
2006	9	20	13	6.247927513
2006	9	21	13	5.857980995
2006	9	22	18	5.459431619
2006	9	23	13	3.321928095
2006	9	24	15	4.321928095
2006	9	25	13	3
2006	9	26	16	3.584962501
2006	9	27	12	2.584962501
2006	9	28	13	3.321928095
2006	9	29	36	3.321928095
2006	9	30	41	2.584962501
2006	10	1	35	1
2006	10	2	33	2
2006	10	3	23	2.584962501
2006	10	4	23	2.584962501
2006	10	5	24	3.807354922
2006	10	6	23	1
2006	10	9	21	1

2007	4	13	0	1
2007	4	19	0	1
2007	4	20	0	1
2007	4	21	0	2
2007	4	22	0	2.584962501
2007	4	23	0	2.584962501
2007	4	24	0	1
2007	4	28	23	1
2007	4	29	32	3.584962501
2007	4	30	34	3.584962501
2007	5	1	33	3.807354922
2007	5	2	33	4.321928095
2007	5	3	20	3.807354922
2007	5	4	18	3
2007	5	5	17	4
2007	5	6	15	4.906890596
2007	5	7	13	5.169925001
2007	5	8	15	3.807354922
2007	5	9	18	3.584962501
2007	5	10	20	4.906890596
2007	5	11	23	2
2007	5	12	22	3
2007	5	13	18	2
2007	5	14	30	4
2007	5	15	30	4.459431619
2007	5	16	32	2.584962501
2007	5	17	40	4.459431619
2007	5	18	42	4.700439718
2007	5	19	37	5.169925001
2007	5	20	23	4.906890596
2007	5	21	18	5.857980995
2007	5	28	0	4
2007	5	29	12	4.700439718
2007	5	30	12	3.584962501
2007	5	31	12	4
2007	6	1	34	5.087462841
2007	6	2	44	2
2007	6	3	55	3.321928095
2007	6	4	60	3.584962501
2007	6	5	62	1
2007	6	6	58	2
2007	6	7	66	2
2007	6	8	60	2
2007	6	9	21	3
2007	6	10	16	1
2007	6	11	16	3.321928095
2007	6	12	14	2.584962501
2007	6	13	14	4.169925001

2007	6	14	0	4.700439718
2007	6	15	0	4.807354922
2007	6	16	0	3.584962501
2007	6	17	0	4.459431619
2007	6	18	0	4.169925001
2007	6	19	0	4.700439718
2007	6	20	0	3.321928095
2007	6	21	0	4.906890596
2007	6	22	0	7.321928095
2007	6	23	0	5
2007	6	24	0	7.95419631
2007	6	25	12	8.499845887
2007	6	26	14	8.129283017
2007	6	27	14	8.787902559
2007	6	28	16	9.082149041
2007	6	29	32	6.700439718
2007	6	30	32	7.022367813
2007	7	1	30	7.475733431
2007	7	2	17	5.247927513
2007	7	3	14	4.459431619
2007	7	4	14	5
2007	7	5	14	4
2007	7	6	14	4.906890596
2007	7	7	12	4.321928095
2007	7	8	16	4.169925001
2007	7	9	22	4
2007	7	10	27	3.807354922
2007	7	11	27	3
2007	7	12	23	3.584962501
2007	7	13	41	4.169925001
2007	7	14	42	2
2007	7	15	39	3.321928095
2007	7	16	31	3
2007	7	17	17	3.584962501
2007	7	18	14	3.807354922
2007	7	19	12	1
2007	7	20	0	2
2007	7	21	0	2
2007	7	22	0	3
2007	7	23	0	1
2007	7	24	0	2.584962501
2007	7	25	0	4.169925001
2007	7	26	0	5.906890596
2007	7	27	0	6.930737338
2007	7	28	14	7.022367813
2007	7	29	14	7.475733431
2007	7	30	14	7.303780748
2007	7	31	0	7.087462841

2007	8	1	0	9.027905997
2007	8	2	0	7.022367813
2007	8	3	11	8.357552005
2007	8	4	11	9.603626345
2007	8	5	11	7.658211483
2007	8	6	13	7.442943496
2007	8	7	13	7.189824559
2007	8	8	15	6.614709844
2007	8	9	15	6.672425342
2007	8	10	15	6.64385619
2007	8	11	13	7.523561956
2007	8	12	13	7.339850003
2007	8	13	13	7.357552005
2007	8	14	0	5.584962501
2007	8	15	0	6.491853096
2007	8	16	0	7.475733431
2007	8	17	0	7.95419631
2007	8	18	0	9.044394119
2007	8	19	0	9.082149041
2007	8	20	0	9.413627929
2007	8	21	15	9.459431619
2007	8	22	13	9.174925683
2007	8	23	13	8.400879436
2007	8	24	13	8.942514505
2007	8	25	13	8.77478706
2007	8	26	13	8.942514505
2007	8	27	13	9.174925683
2007	8	28	13	9.044394119
2007	8	29	13	9.238404739
2007	8	30	13	9.388017285
2007	8	31	28	5.321928095
2007	9	1	24	6.169925001
2007	9	2	13	5.392317423
2007	9	3	13	4.169925001
2007	9	4	13	4.584962501
2007	9	5	13	4.169925001
2007	9	6	0	3.807354922
2007	9	7	0	4.906890596
2007	9	8	0	5.392317423
2007	9	9	0	4.169925001
2007	9	10	0	5.523561956
2007	9	11	0	4.169925001
2007	9	12	0	4.459431619
2007	9	13	0	4.906890596
2007	9	14	0	6.087462841
2007	9	15	0	5.700439718
2007	9	16	0	5.584962501
2007	9	17	0	4.700439718

2007	9	18	0	5.584962501
2007	9	19	0	7.108524457
2007	9	20	0	6.857980995
2007	9	21	0	6.614709844
2007	9	22	0	6.392317423
2007	9	23	0	4.321928095
2007	9	24	0	4.906890596
2007	9	25	0	3.584962501
2007	9	26	0	3.807354922
2007	9	27	0	3.321928095
2007	9	28	15	3.584962501
2007	9	29	15	3.584962501
2007	9	30	15	3
2007	10	1	0	2
2007	10	2	0	2.584962501
2007	10	3	0	3
2007	10	4	0	3
2007	10	5	0	4
2007	10	6	19	2
2007	10	7	16	1
2007	10	8	12	1
2008	4	26	0	1
2008	4	29	0	3.321928095
2008	4	30	0	2.584962501
2008	5	2	0	1
2008	5	3	0	3
2008	5	4	10	2
2008	5	5	12	4
2008	5	6	0	1
2008	5	7	0	2
2008	5	8	0	2
2008	5	9	0	1
2008	5	10	0	1
2008	5	11	0	1
2008	5	13	12	2
2008	5	14	0	1
2008	5	18	22	3
2008	5	19	16	1
2008	5	21	0	2.584962501
2008	5	22	0	2
2008	5	23	0	1
2008	5	24	0	3
2008	5	25	0	1
2008	5	26	0	2
2008	5	27	0	1
2008	5	28	0	2
2008	5	29	0	2
2008	5	30	0	2.584962501

2008	5	31	0	2
2008	6	1	0	2
2008	6	2	0	3.584962501
2008	6	4	0	2.584962501
2008	6	5	14	2.584962501
2008	6	6	0	3
2008	6	7	0	2
2008	6	8	0	2
2008	6	9	0	3
2008	6	10	14	3.321928095
2008	6	11	12	3.807354922
2008	6	12	11	3.584962501
2008	6	13	12	4.906890596
2008	6	14	0	5
2008	6	15	11	6
2008	6	16	11	4.169925001
2008	6	17	11	5.64385619
2008	6	18	12	5
2008	6	19	11	3.584962501
2008	6	20	12	2.584962501
2008	6	21	12	7.977279923
2008	6	22	12	5.857980995
2008	6	23	0	3.584962501
2008	6	24	0	2.584962501
2008	6	25	0	3.807354922
2008	6	26	0	7.700439718
2008	6	27	0	6.247927513
2008	6	28	0	3.321928095
2008	6	29	0	5.807354922
2008	6	30	0	5.523561956
2008	7	2	0	2.584962501
2008	7	3	0	4.321928095
2008	7	4	0	5.459431619
2008	7	5	0	4.169925001
2008	7	6	0	6.700439718
2008	7	7	0	5.169925001
2008	7	8	0	3
2008	7	9	0	5.087462841
2008	7	10	0	1
2008	7	11	0	1
2008	7	12	0	1
2008	7	14	0	3.321928095
2008	7	15	0	1
2008	7	16	0	1
2008	7	17	0	2
2008	7	18	6	1
2008	7	19	7	3.321928095
2008	7	20	6	2.584962501

2008	7	21	0	3
2008	7	22	0	2.584962501
2008	7	23	0	2
2008	7	24	0	3.321928095
2008	7	25	0	3.321928095
2008	7	26	0	2.584962501
2008	7	27	0	4.169925001
2008	7	28	0	4.169925001
2008	7	29	0	5
2008	7	30	0	5.523561956
2008	7	31	0	5.523561956
2008	8	1	0	5.459431619
2008	8	2	0	5
2008	8	3	0	5.584962501
2008	8	4	0	7.906890596
2008	8	5	0	6.321928095
2008	8	6	0	7.189824559
2008	8	7	0	6.781359714
2008	8	8	0	6.832890014
2008	8	9	0	6.554588852
2008	8	10	0	5.64385619
2008	8	11	0	7.857980995
2008	8	12	0	6.727920455
2008	8	13	0	5.64385619
2008	8	14	0	7.409390936
2008	8	15	0	4.906890596
2008	8	16	0	6.930737338
2008	8	17	0	5.754887502
2008	8	18	0	1
2008	8	19	0	3
2008	8	20	0	3
2008	8	21	5	4
2008	8	22	5	3.584962501
2008	8	23	0	2.584962501
2008	8	24	0	2
2008	8	25	0	5.321928095
2008	8	26	0	5.906890596
2008	8	27	0	5.95419631
2008	8	28	0	4.459431619
2008	8	29	0	5.169925001
2008	8	30	0	6.426264755
2008	8	31	0	4.700439718
2008	9	1	0	5.247927513
2008	9	2	0	3
2008	9	3	0	7.686500527
2008	9	4	0	6.754887502
2008	9	6	0	4.807354922
2008	9	7	0	7.22881869

2008	9	8	0	3
2008	9	9	0	2.584962501
2008	9	10	0	4.807354922
2008	9	11	7	3
2008	9	14	0	3.807354922
2008	9	15	0	2.584962501
2008	9	16	0	3.321928095
2008	9	17	0	2
2008	9	18	0	1
2008	9	20	0	3.807354922
2008	9	21	0	3.321928095
2008	9	22	9	3
2008	9	23	10	3.321928095
2008	9	24	0	1
2008	9	26	0	1
2008	9	27	0	1
2008	9	28	0	2
2008	9	29	9	1
2008	9	30	0	1
2009	4	13	0	2.584962501
2009	4	14	0	1
2009	4	15	0	3.584962501
2009	4	16	0	1
2009	4	18	0	2
2009	4	19	0	1
2009	4	20	0	1
2009	4	21	11	3.584962501
2009	4	22	0	2.584962501
2009	4	25	0	3
2009	4	26	0	3.807354922
2009	4	27	0	3.321928095
2009	4	28	0	1
2009	4	29	13	2
2009	4	30	13	2.584962501
2009	5	1	0	3.584962501
2009	5	2	0	4
2009	5	3	0	2
2009	5	4	7	2
2009	5	5	0	5
2009	5	8	0	1
2009	5	9	0	2.584962501
2009	5	10	0	4.584962501
2009	5	11	0	2.584962501
2009	5	12	0	3.584962501
2009	5	13	7	3.321928095
2009	5	14	10	5.523561956
2009	5	15	9	4.459431619
2009	5	16	8	3.321928095

2009	5	17	9	2
2009	5	18	8	3
2009	5	19	7	4
2009	5	20	0	4
2009	5	21	0	4
2009	5	22	8	2
2009	5	23	8	4.321928095
2009	5	24	0	4.700439718
2009	5	27	0	1
2009	5	28	0	1
2009	5	29	0	2
2009	5	31	10	3.321928095
2009	6	1	24	1
2009	6	2	26	3.321928095
2009	6	4	19	2.584962501
2009	6	5	17	3.584962501
2009	6	6	0	3.584962501
2009	6	8	0	2.584962501
2009	6	9	0	4.169925001
2009	6	10	0	5.459431619
2009	6	11	0	6.247927513
2009	6	12	0	5.906890596
2009	6	13	0	6.491853096
2009	6	14	0	3.807354922
2009	6	15	0	5.906890596
2009	6	16	0	6.906890596
2009	6	17	15	7.321928095
2009	6	18	0	3.584962501
2009	6	19	0	4.321928095
2009	6	20	0	4.584962501
2009	6	21	15	4
2009	6	22	17	7.044394119
2009	6	23	15	8.285402219
2009	6	24	17	8.011227255
2009	6	25	0	7
2009	6	26	0	7.741466986
2009	6	27	0	6.584962501
2009	6	28	0	7.475733431
2009	6	29	0	7.794415866
2009	6	30	0	5.392317423
2009	7	1	0	3.584962501
2009	7	2	0	4.700439718
2009	7	3	0	4.584962501
2009	7	4	24	6.044394119
2009	7	5	27	2
2009	7	6	24	2.584962501
2009	7	7	19	4.321928095
2009	7	8	21	4.459431619

2009	7	9	17	5.459431619
2009	7	10	15	5
2009	7	11	0	5.523561956
2009	7	12	0	5.754887502
2009	7	13	0	4.700439718
2009	7	14	0	5
2009	7	15	0	3.807354922
2009	7	16	0	4.321928095
2009	7	17	0	3.807354922
2009	7	18	0	3.584962501
2009	7	19	0	6.392317423
2009	7	20	0	6.700439718
2009	7	21	0	7.87036472
2009	7	22	0	7.06608919
2009	7	23	12	7.741466986
2009	7	24	0	6.209453366
2009	7	25	0	5.321928095
2009	7	26	0	4.321928095
2009	7	27	0	5.392317423
2009	7	28	0	8.033423002
2009	7	29	0	9.584962501
2009	7	30	12	5.95419631
2009	7	31	0	7.894817763
2009	8	1	0	7.882643049
2009	8	2	0	8.14974712
2009	8	3	0	6.392317423
2009	8	4	0	6.930737338
2009	8	5	0	5.321928095
2009	8	6	0	4.321928095
2009	8	7	0	6.129283017
2009	8	8	0	7.303780748
2009	8	9	0	7.14974712
2009	8	10	0	5.857980995
2009	8	11	0	6.392317423
2009	8	12	0	7.108524457
2009	8	13	0	6.807354922
2009	8	14	0	6.357552005
2009	8	15	0	6
2009	8	16	0	7.807354922
2009	8	17	0	6.285402219
2009	8	18	0	7.584962501
2009	8	19	0	5.906890596
2009	8	20	0	6.754887502
2009	8	21	0	5.247927513
2009	8	22	0	5.64385619
2009	8	23	0	4.584962501
2009	8	24	0	3.584962501
2009	8	25	0	3.321928095

2009	8	26	0	4.321928095
2009	8	27	0	4
2009	8	28	0	2.584962501
2009	8	30	0	1
2009	8	31	0	2.584962501
2009	9	1	13	4.459431619
2009	9	2	0	2
2009	9	3	0	2
2009	9	4	0	2.584962501
2009	9	5	0	1
2009	9	7	0	1
2009	9	8	0	4.169925001
2009	9	9	0	1
2009	9	10	0	4.906890596
2009	9	11	0	3.584962501
2009	9	12	0	4.584962501
2009	9	13	0	2.584962501
2009	9	14	0	1
2009	9	15	0	3
2009	9	16	0	3.321928095
2009	9	17	0	5.754887502
2009	9	19	0	3
2009	9	20	0	2.584962501
2009	9	21	13	4
2009	9	22	22	5.392317423
2009	9	23	33	5.321928095
2009	9	24	33	3.321928095
2009	9	25	28	1
2009	9	26	15	1
2009	9	28	17	1
2010	4	11	10	1
2010	4	14	0	1
2010	4	15	0	1
2010	4	16	0	2.584962501
2010	4	18	0	1
2010	4	19	0	1
2010	4	22	9	1
2010	5	1	13	2.584962501
2010	5	2	21	4
2010	5	3	40	4.459431619
2010	5	4	51	4
2010	5	5	47	3
2010	5	6	19	3
2010	5	7	16	3.321928095
2010	5	8	16	4
2010	5	9	0	2
2010	5	10	0	4.906890596
2010	5	11	0	4.906890596

2010	5	12	0	5.247927513
2010	5	13	0	4.700439718
2010	5	14	0	4.169925001
2010	5	15	0	4.459431619
2010	5	16	0	3.584962501
2010	5	17	0	3.321928095
2010	5	18	0	2.584962501
2010	5	19	0	2
2010	5	20	0	1
2010	5	21	14	1
2010	5	22	21	4.459431619
2010	5	23	22	3
2010	5	24	19	2
2010	5	26	16	3
2010	5	27	13	2.584962501
2010	5	28	13	1
2010	5	30	29	1
2010	5	31	18	1
2010	6	1	15	2
2010	6	2	18	1
2010	6	3	21	1
2010	6	4	28	2
2010	6	5	26	1
2010	6	6	12	2
2010	6	7	18	2.584962501
2010	6	8	14	3.321928095
2010	6	9	29	2
2010	6	10	39	3
2010	6	11	46	3.584962501
2010	6	13	25	2
2010	6	14	14	3.584962501
2010	6	15	0	1
2010	6	16	0	4.584962501
2010	6	17	12	5.087462841
2010	6	18	12	2
2010	6	19	26	4.700439718
2010	6	20	18	3
2010	6	22	15	4
2010	6	23	14	2
2010	6	24	12	5.169925001
2010	6	25	17	3.584962501
2010	6	26	18	1
2010	6	27	11	3.584962501
2010	6	28	11	6.209453366
2010	6	29	22	5.087462841
2010	6	30	11	7.614709844
2010	7	1	13	7.554588852
2010	7	2	13	8.011227255

2010	7	3	13	7.108524457
2010	7	4	14	7.321928095
2010	7	5	27	7.375039431
2010	7	6	25	6.392317423
2010	7	7	13	3.807354922
2010	7	8	13	7.321928095
2010	7	9	17	7.523561956
2010	7	10	22	7.894817763
2010	7	11	36	8.044394119
2010	7	12	34	5.857980995
2010	7	13	30	4.700439718
2010	7	14	17	3.807354922
2010	7	15	25	3.584962501
2010	7	16	16	4.169925001
2010	7	17	14	3.807354922
2010	7	18	14	3.321928095
2010	7	19	14	4.169925001
2010	7	20	30	4.321928095
2010	7	21	31	3.321928095
2010	7	22	36	2
2010	7	23	44	3.584962501
2010	7	24	47	3.321928095
2010	7	25	39	1
2010	7	26	33	3
2010	7	27	19	2.584962501
2010	7	29	38	4.807354922
2010	7	30	31	2.584962501
2010	7	31	30	2
2010	8	1	24	1
2010	8	2	24	4
2010	8	3	14	4.459431619
2010	8	4	32	4
2010	8	5	51	5.807354922
2010	8	6	51	5.95419631
2010	8	7	48	7.22881869
2010	8	8	38	7.108524457
2010	8	9	55	7.375039431
2010	8	10	58	8.14974712
2010	8	11	67	6.285402219
2010	8	12	50	6.209453366
2010	8	13	41	7.965784285
2010	8	14	33	8.741466986
2010	8	15	38	7.754887502
2010	8	16	51	8.189824559
2010	8	17	26	7.781359714
2010	8	18	23	7.044394119
2010	8	19	15	4.459431619
2010	8	20	12	4.906890596

2010	8	22	0	5.247927513
2010	8	23	0	6.169925001
2010	8	24	12	5.169925001
2010	8	25	23	4.169925001
2010	8	26	23	6.209453366
2010	8	27	12	3.321928095
2010	8	28	12	3
2010	8	29	27	4.169925001
2010	8	30	29	4.584962501
2010	8	31	30	6.426264755
2010	9	1	30	6.857980995
2010	9	2	58	7.209453366
2010	9	3	49	6.727920455
2010	9	4	58	6.491853096
2010	9	5	43	5.169925001
2010	9	6	29	6.087462841
2010	9	7	14	5.392317423
2010	9	8	0	5.95419631
2010	9	9	0	5.087462841
2010	9	10	16	5.700439718
2010	9	11	12	6.491853096
2010	9	12	13	5.906890596
2010	9	13	26	7.918863237
2010	9	14	27	6.584962501
2010	9	15	27	3.807354922
2010	9	16	43	6.523561956
2010	9	17	53	4.169925001
2010	9	18	51	5
2010	9	19	49	1
2010	9	21	38	2.584962501
2010	9	22	36	2.584962501
2010	9	23	39	3.321928095
2010	9	25	39	2
2010	9	26	49	1
2010	9	27	56	1
2010	9	29	55	1
2010	9	30	48	4.169925001
2011	4	20	73	1
2011	4	24	64	1
2011	4	29	62	1
2011	5	1	70	1
2011	5	3	71	2
2011	5	4	76	1
2011	5	5	76	3.584962501
2011	5	6	45	6.247927513
2011	5	7	43	5.247927513
2011	5	8	63	4.700439718
2011	5	9	84	1

2011	5	10	90	3.807354922
2011	5	11	66	3.321928095
2011	5	12	46	2.584962501
2011	5	13	36	3
2011	5	14	52	3
2011	5	15	57	2
2011	5	16	57	2.584962501
2011	5	17	50	3
2011	5	18	41	3
2011	5	19	36	3.321928095
2011	5	20	35	2.584962501
2011	5	21	36	1
2011	5	23	31	2
2011	5	24	11	2.584962501
2011	5	25	24	3.321928095
2011	5	26	42	4.169925001
2011	5	27	63	4.169925001
2011	5	28	73	2.584962501
2011	5	29	98	1
2011	6	5	80	3
2011	6	6	70	3
2011	6	11	23	2
2011	6	12	17	1
2011	6	15	52	1
2011	6	16	59	4.584962501
2011	6	17	59	2.584962501
2011	6	18	61	4.807354922
2011	6	19	47	4.321928095
2011	6	20	39	4
2011	6	21	50	6.357552005
2011	6	22	50	7.22881869
2011	6	23	58	6.169925001
2011	6	24	65	5.087462841
2011	6	25	50	6.554588852
2011	6	26	27	8.257387843
2011	6	27	15	5.169925001
2011	6	28	38	7.882643049
2011	6	29	41	7.022367813
2011	6	30	49	6.614709844
2011	7	1	56	5.392317423
2011	7	2	44	3.584962501
2011	7	3	41	6.700439718
2011	7	4	46	4.459431619
2011	7	5	32	2.584962501
2011	7	6	34	6.459431619
2011	7	7	38	4.321928095
2011	7	8	63	4.321928095
2011	7	9	49	3.321928095

2011	7	10	59	4
2011	7	14	68	1
2011	7	18	115	3.584962501
2011	7	19	109	1
2011	7	20	71	2.584962501
2011	7	21	60	1
2011	7	22	54	3.584962501
2011	7	23	43	3.321928095
2011	7	24	43	4.459431619
2011	7	25	29	5.169925001
2011	7	26	43	4.807354922
2011	7	27	63	5.087462841
2011	7	28	96	5
2011	7	29	90	4.906890596
2011	7	30	91	7.303780748
2011	7	31	99	4.321928095
2011	8	1	107	4.584962501
2011	8	2	95	5.321928095
2011	8	3	81	4.906890596
2011	8	4	92	5.459431619
2011	8	5	88	5.807354922
2011	8	6	79	5.087462841
2011	8	7	79	5.807354922
2011	8	8	70	7.491853096
2011	8	9	61	7.794415866
2011	8	10	36	7.475733431
2011	8	11	38	6.087462841
2011	8	12	29	5.169925001
2011	8	13	18	5.392317423
2011	8	14	0	5.459431619
2011	8	15	12	4.906890596
2011	8	16	31	4.584962501
2011	8	18	59	7.044394119
2011	8	19	60	4.906890596
2011	8	20	59	5
2011	8	21	73	4.459431619
2011	8	22	92	3.321928095
2011	8	23	98	5
2011	8	24	70	5.857980995
2011	8	25	65	5.95419631
2011	8	26	86	6.357552005
2011	8	27	77	6.209453366
2011	8	28	64	6.321928095
2011	8	29	56	4.807354922
2011	8	30	90	4.906890596
2011	8	31	125	5
2011	9	1	131	5.906890596
2011	9	2	136	5.807354922

2011	9	3	140	4
2011	9	6	89	1
2011	9	7	72	3.807354922
2011	9	8	54	2
2011	9	10	80	2.584962501
2011	9	11	94	2
2011	9	12	139	1
2011	9	13	145	4.459431619
2011	9	14	169	4.169925001
2011	9	15	191	5.321928095
2011	9	16	191	6
2011	9	17	160	6.247927513
2011	9	18	143	4.584962501
2011	9	19	142	4.321928095
2011	9	20	123	4.459431619
2011	9	24	116	1
2011	9	25	122	1
2011	9	27	103	1
2012	4	22	146	1
2012	4	23	138	1
2012	4	25	126	1
2012	4	26	117	1
2012	4	30	104	2
2012	5	2	95	1
2012	5	3	96	2
2012	5	4	87	1
2012	5	8	85	2
2012	5	11	110	2
2012	5	12	101	1
2012	5	13	105	4
2012	5	14	119	3
2012	5	15	122	2.584962501
2012	5	16	137	3
2012	5	17	110	1
2012	5	18	102	2
2012	5	19	109	3
2012	5	20	116	3.321928095
2012	5	21	110	2
2012	5	22	80	3.584962501
2012	5	23	77	1
2012	5	24	87	3.321928095
2012	5	25	94	2.584962501
2012	5	26	80	3.807354922
2012	5	27	82	1
2012	5	29	78	2
2012	5	31	89	2
2012	6	1	121	2
2012	6	3	165	1

2012	6	6	153	1
2012	6	7	127	3
2012	6	8	107	2
2012	6	13	113	2
2012	6	15	117	1
2012	6	16	106	2.584962501
2012	6	18	57	1
2012	6	19	51	4
2012	6	20	30	4.584962501
2012	6	21	16	4.906890596
2012	6	22	16	3.321928095
2012	6	23	16	2
2012	6	24	20	4.169925001
2012	6	25	17	5.087462841
2012	6	26	36	3.807354922
2012	6	27	70	3.807354922
2012	6	28	87	3.321928095
2012	6	29	103	3.584962501
2012	6	30	104	2.584962501
2012	7	1	126	3.321928095
2012	7	2	128	2
2012	7	3	125	4
2012	7	4	131	3.321928095
2012	7	5	129	4.584962501
2012	7	6	132	4.807354922
2012	7	7	146	4
2012	7	8	117	4.807354922
2012	7	9	122	4.807354922
2012	7	10	107	4.700439718
2012	7	11	110	4.906890596
2012	7	12	125	4.584962501
2012	7	13	129	2
2012	7	14	125	3.584962501
2012	7	15	117	2.584962501
2012	7	16	102	2
2012	7	17	86	1
2012	7	18	57	2
2012	7	19	38	1
2012	7	20	36	1
2012	7	21	29	3
2012	7	22	29	1
2012	7	23	63	2
2012	7	24	71	3.321928095
2012	7	25	68	1
2012	7	26	86	2
2012	7	30	120	1
2012	8	1	132	1
2012	8	2	143	3.321928095

2012	8	3	174	3
2012	8	4	152	1
2012	8	5	123	1
2012	8	6	98	4
2012	8	7	122	5.087462841
2012	8	8	123	2
2012	8	9	158	1
2012	8	10	131	3
2012	8	11	108	4.321928095
2012	8	12	98	4.321928095
2012	8	13	74	4.807354922
2012	8	14	33	3.807354922
2012	8	15	30	4
2012	8	16	32	6.285402219
2012	8	17	50	5.459431619
2012	8	18	54	7.64385619
2012	8	19	72	5.584962501
2012	8	20	80	6.614709844
2012	8	21	71	4.906890596
2012	8	22	69	2
2012	8	23	71	3.321928095
2012	8	24	71	5.754887502
2012	8	25	77	2
2012	8	26	78	5.169925001
2012	8	27	75	3.321928095
2012	8	28	80	2.584962501
2012	8	29	83	1
2012	8	31	150	3
2012	9	1	145	3.321928095
2012	9	2	142	4.169925001
2012	9	3	171	1
2012	9	4	159	3.584962501
2012	9	5	122	3
2012	9	6	116	2
2012	9	7	95	5.321928095
2012	9	8	82	4.169925001
2012	9	9	89	3.807354922
2012	9	10	70	1
2012	9	11	76	1
2012	9	12	73	1
2012	9	13	58	1
2012	9	15	61	1
2012	9	16	64	3
2012	9	21	84	2
2012	9	22	58	2.584962501
2012	9	23	75	2
2012	9	24	95	1
2012	9	27	113	1

2012	9	30	99	1
2013	4	16	93	3.584962501
2013	4	18	85	1
2013	4	21	93	1
2013	4	24	98	1
2013	4	26	105	2.584962501
2013	4	27	107	2
2013	4	28	102	4.459431619
2013	4	29	141	3.584962501
2013	4	30	150	4
2013	5	1	142	2.584962501
2013	5	2	104	3.321928095
2013	5	3	98	5.584962501
2013	5	4	122	2
2013	5	5	110	2.584962501
2013	5	6	108	3.321928095
2013	5	7	116	3.321928095
2013	5	8	122	2.584962501
2013	5	9	116	4.584962501
2013	5	10	125	5.087462841
2013	5	11	134	5.169925001
2013	5	12	142	4.169925001
2013	5	15	173	3
2013	5	16	206	2
2013	5	17	183	3.321928095
2013	5	18	133	2
2013	5	19	127	3
2013	5	20	112	2.584962501
2013	5	21	113	3.321928095
2013	5	22	131	2
2013	5	23	108	3
2013	5	24	108	1
2013	6	2	77	1
2013	6	3	64	3
2013	6	4	51	2
2013	6	5	79	3.321928095
2013	6	6	76	1
2013	6	7	48	1
2013	6	8	26	2
2013	6	9	35	1
2013	6	10	26	1
2013	6	11	16	3.807354922
2013	6	12	29	3
2013	6	13	51	4.321928095
2013	6	14	64	3
2013	6	16	104	4.169925001
2013	6	19	123	2.584962501
2013	6	20	129	3

2013	6	21	139	4
2013	6	22	137	2.584962501
2013	6	23	118	1
2013	6	24	108	3.807354922
2013	6	25	77	3
2013	6	26	60	5.584962501
2013	6	27	61	6.209453366
2013	6	28	75	6.169925001
2013	6	29	69	4
2013	6	30	80	5
2013	7	1	77	2
2013	7	4	121	3
2013	7	5	109	3.321928095
2013	7	6	124	4.459431619
2013	7	7	141	4.321928095
2013	7	8	122	3.321928095
2013	7	9	110	3
2013	7	10	80	1
2013	7	11	79	3
2013	7	12	73	1
2013	7	13	36	2.584962501
2013	7	14	60	3.321928095
2013	7	15	77	2.584962501
2013	7	16	77	1
2013	7	18	95	2.584962501
2013	7	19	85	1
2013	7	20	59	2.584962501
2013	7	23	80	1
2013	7	24	70	3.321928095
2013	7	25	85	1
2013	7	26	71	1
2013	7	27	68	2
2013	7	28	73	3
2013	7	29	103	3.584962501
2013	7	30	89	3.584962501
2013	8	1	96	4.169925001
2013	8	2	114	6.321928095
2013	8	3	107	6.491853096
2013	8	4	86	6.754887502
2013	8	5	82	4.700439718
2013	8	6	71	4.169925001
2013	8	7	86	3.584962501
2013	8	8	89	5.754887502
2013	8	9	64	5.087462841
2013	8	10	72	6.247927513
2013	8	13	108	5.584962501
2013	8	14	108	2.584962501
2013	8	15	124	3

2013	8	16	96	5.392317423
2013	8	17	100	3.584962501
2013	8	18	110	2
2013	8	19	134	5.087462841
2013	8	20	129	4.700439718
2013	8	21	146	3
2013	8	23	138	3
2013	8	24	78	5.807354922
2013	8	25	50	3
2013	8	28	50	1
2013	8	30	71	1
2013	9	4	78	2
2013	9	5	65	1
2013	9	6	49	2
2013	9	13	37	2.584962501
2013	9	14	25	1
2013	9	17	52	1
2014	4	24	72	2.584962501
2014	4	28	80	1
2014	4	29	77	2
2014	4	30	82	2
2014	5	3	123	1
2014	5	4	127	2
2014	5	5	142	3
2014	5	6	148	2
2014	5	7	120	3
2014	5	8	132	3
2014	5	9	139	2
2014	5	10	123	3
2014	5	11	150	2.584962501
2014	5	12	154	2
2014	5	13	133	3.321928095
2014	5	14	166	2.584962501
2014	5	15	156	2.584962501
2014	5	16	133	2.584962501
2014	5	17	151	1
2014	5	18	138	2.584962501
2014	5	19	102	2
2014	5	20	87	1
2014	5	21	85	2.584962501
2014	5	22	66	2
2014	5	23	91	1
2014	5	24	109	4.169925001
2014	5	25	109	2
2014	5	26	91	3
2014	5	27	78	1
2014	5	28	70	1
2014	5	31	57	1

2014	6	4	64	1
2014	6	7	133	2.584962501
2014	6	8	142	2.584962501
2014	6	15	74	1
2014	6	16	84	2
2014	6	18	120	1
2014	6	22	90	2.584962501
2014	6	23	77	1
2014	6	25	55	2
2014	6	29	93	1
2014	7	1	134	3
2014	7	2	145	5.321928095
2014	7	7	197	1
2014	7	8	183	1
2014	7	13	86	1
2014	7	15	18	1
2014	7	16	10	3
2014	7	18	15	1
2014	7	24	62	2
2014	7	26	58	3.321928095
2014	7	27	78	2.584962501
2014	7	28	109	4
2014	7	29	137	4
2014	7	30	122	3.807354922
2014	7	31	131	2.584962501
2014	8	1	165	4
2014	8	2	165	2
2014	8	3	153	3.321928095
2014	8	4	146	1
2014	8	5	128	2
2014	8	6	113	1
2014	8	7	123	2
2014	8	13	79	3.321928095
2014	8	14	86	2.584962501
2014	8	15	107	3.584962501
2014	8	16	109	4.169925001
2014	8	17	116	5.392317423
2014	8	18	105	2.584962501
2014	8	19	93	1
2014	8	20	102	1
2014	8	21	120	2.584962501
2014	8	22	125	2
2014	8	23	150	3
2014	8	24	149	3.321928095
2014	8	25	116	5.459431619
2014	8	26	86	5.906890596
2014	8	27	95	6.321928095
2014	8	28	82	3.584962501

2014	8	29	66	4
2014	8	30	76	5.321928095
2014	8	31	82	6.129283017
2014	9	1	96	2.584962501
2014	9	2	110	5.247927513
2014	9	3	102	3.321928095
2014	9	4	120	2.584962501
2014	9	5	116	4.321928095
2014	9	6	142	2
2014	9	7	165	2.584962501
2014	9	14	104	1
2014	9	20	80	3.321928095
2015	4	26	65	1
2015	5	4	103	2
2015	5	5	104	3
2015	5	7	145	3.321928095
2015	5	8	150	2
2015	5	9	139	2.584962501
2015	5	10	163	2.584962501
2015	5	11	163	5
2015	5	12	165	5
2015	5	13	172	3.807354922
2015	5	14	164	2.584962501
2015	5	15	124	1
2015	5	16	103	1
2015	5	17	89	2.584962501
2015	5	22	73	1
2015	5	23	72	2
2015	5	26	53	1
2015	5	27	14	1
2015	5	28	12	1
2015	5	31	29	1
2015	6	1	27	2
2015	6	9	92	2
2015	6	11	95	4
2015	6	12	98	2.584962501
2015	6	13	97	1
2015	6	14	90	3.584962501
2015	6	15	54	5.754887502
2015	6	16	70	3
2015	6	17	80	3.321928095
2015	6	18	77	3.321928095
2015	6	19	68	4.700439718
2015	6	20	64	4.906890596
2015	6	21	61	5
2015	6	22	56	3.584962501
2015	6	23	45	3
2015	6	24	36	1

2015	6	25	27	3
2015	6	26	23	1
2015	6	27	21	4.906890596
2015	6	28	32	5.087462841
2015	6	29	39	1
2015	6	30	41	1
2015	7	8	123	1
2015	7	9	110	3.321928095
2015	7	10	103	2.584962501
2015	7	13	53	1
2015	7	14	43	2
2015	7	15	43	3.321928095
2015	7	16	50	3.584962501
2015	7	17	50	5.087462841
2015	7	18	58	5.247927513
2015	7	19	44	4.169925001
2015	7	20	36	3.807354922
2015	7	21	36	5.523561956
2015	7	22	36	6.087462841
2015	7	23	29	5.700439718
2015	7	24	31	6.209453366
2015	7	25	34	6.285402219
2015	7	26	40	5.754887502
2015	7	27	51	4.321928095
2015	7	28	62	2.584962501
2015	7	29	67	4.169925001
2015	7	30	76	2.584962501
2015	7	31	69	3.321928095
2015	8	1	64	4.459431619
2015	8	2	53	5.169925001
2015	8	3	61	5.64385619
2015	8	4	90	4.906890596
2015	8	5	94	4.700439718
2015	8	6	108	2.584962501
2015	8	7	112	3.321928095
2015	8	8	93	2.584962501
2015	8	9	76	2
2015	8	10	69	4
2015	8	11	78	4.459431619
2015	8	12	66	4.807354922
2015	8	13	55	2.584962501
2015	8	14	46	4
2015	8	15	39	4.321928095
2015	8	16	38	2
2015	8	17	33	3.321928095
2015	8	18	39	3.321928095
2015	8	19	50	2
2015	8	20	65	2.584962501

2015	8	21	73	2.584962501
2015	8	22	71	4.807354922
2015	8	23	81	4.459431619
2015	8	25	69	5.584962501
2015	8	26	47	5.807354922
2015	8	27	55	6.672425342
2015	8	28	44	6
2015	8	29	56	5.584962501
2015	8	30	56	7.087462841
2015	8	31	35	5.857980995
2015	9	1	43	5.247927513
2015	9	2	36	3.584962501
2015	9	3	29	2.584962501
2015	9	4	37	4.459431619
2015	9	5	27	3
2015	9	6	43	4.321928095
2015	9	7	44	1
2015	9	9	57	3.584962501
2015	9	10	46	2.584962501
2015	9	11	81	3.321928095
2015	9	12	89	4.169925001
2015	9	13	83	3.807354922
2015	9	14	56	3.321928095
2015	9	15	68	3
2015	9	16	86	2.584962501
2015	9	17	75	6.930737338
2015	9	18	71	2
2015	9	19	64	2.584962501
2015	9	22	86	1
2015	9	23	96	3
2015	9	24	101	1
2015	9	25	152	1
2015	9	26	162	1
2015	9	27	169	2
2016	4	19	28	1
2016	4	26	57	1
2016	4	30	89	2.584962501
2016	5	1	87	2.584962501
2016	5	2	83	2.584962501
2016	5	3	73	4.321928095
2016	5	4	71	3
2016	5	6	57	2
2016	5	19	43	1
2016	5	27	34	1
2016	6	2	14	1
2016	6	8	14	1
2016	6	10	33	2.584962501
2016	6	11	44	3.584962501

2016	6	13	38	3
2016	6	14	38	4
2016	6	15	40	3.321928095
2016	6	16	28	2
2016	6	17	39	1
2016	6	20	36	3
2016	6	21	24	4.459431619
2016	6	22	23	5.247927513
2016	6	23	12	6.357552005
2016	6	24	12	6.727920455
2016	6	25	0	6.357552005
2016	6	26	0	6.584962501
2016	6	27	0	5.64385619
2016	6	28	0	6.523561956
2016	6	29	0	5.584962501
2016	6	30	0	4.700439718
2016	7	1	0	4
2016	7	2	11	4.321928095
2016	7	3	19	4.584962501
2016	7	4	0	3
2016	7	5	13	2
2016	7	6	13	3
2016	7	7	25	2.584962501
2016	7	8	41	1
2016	7	9	56	1
2016	7	11	62	1
2016	7	12	59	2.584962501
2016	7	13	53	1
2016	7	14	58	3
2016	7	18	64	1
2016	7	19	59	1
2016	7	23	27	1
2016	7	24	13	2
2016	7	26	0	4
2016	7	27	0	3.321928095
2016	7	28	13	3.584962501
2016	7	29	15	2.584962501
2016	7	30	21	1
2016	7	31	16	3
2016	8	1	12	2.584962501
2016	8	2	12	3.807354922
2016	8	3	0	3.584962501
2016	8	4	16	3.807354922
2016	8	5	38	3
2016	8	6	39	3
2016	8	7	51	3.321928095
2016	8	9	72	1
2016	8	11	79	3

2016	8	12	71	4
2016	8	13	58	1
2016	8	14	59	3.807354922
2016	8	15	63	3
2016	8	16	80	2
2016	8	17	58	3
2016	8	18	49	3
2016	8	19	47	2
2016	8	20	16	2.584962501
2016	8	22	28	1
2016	8	23	48	2
2016	8	24	49	2.584962501
2016	8	25	48	3.807354922
2016	8	26	49	4.906890596
2016	8	27	58	5.392317423
2016	8	28	64	5.95419631
2016	8	29	68	3.807354922
2016	8	30	74	5.321928095
2016	8	31	77	4.700439718
2016	9	1	78	1
2016	9	2	67	2.584962501
2016	9	3	65	2
2016	9	4	49	2.584962501
2016	9	5	31	2
2016	9	6	43	1
2016	9	7	57	1
2016	9	8	53	1
2016	9	9	79	1
2016	9	10	78	2.584962501
2016	9	17	14	1
2017	4	27	26	1
2017	4	28	36	1
2017	4	29	34	2.584962501
2017	4	30	24	3
2017	5	1	12	3.807354922
2017	5	2	29	4.584962501
2017	5	3	18	4.169925001
2017	5	4	17	4
2017	5	5	31	3.321928095
2017	5	6	27	3.321928095
2017	5	7	23	3
2017	5	8	11	3.584962501
2017	5	9	0	3
2017	5	10	0	3.321928095
2017	5	11	11	4.807354922
2017	5	12	15	5.169925001
2017	5	13	0	5.523561956
2017	5	14	0	5.247927513

2017	5	15	0	5.169925001
2017	5	16	25	4.584962501
2017	5	17	12	3.584962501
2017	5	18	24	4
2017	5	19	25	3.807354922
2017	5	20	30	3.584962501
2017	5	21	37	3.321928095
2017	5	22	50	4.459431619
2017	5	23	55	3.807354922
2017	5	24	20	3.807354922
2017	5	25	31	5.321928095
2017	5	26	25	4.906890596
2017	5	27	24	4.807354922
2017	5	28	21	4
2017	5	29	14	2
2017	5	30	0	2.584962501
2017	6	7	14	1
2017	6	8	14	2
2017	6	9	0	2
2017	6	10	0	2.584962501
2017	6	11	0	3
2017	6	12	0	3.321928095
2017	6	13	11	2.584962501
2017	6	14	11	3.321928095
2017	6	15	31	2
2017	6	16	30	2
2017	6	17	30	3.807354922
2017	6	18	29	4.906890596
2017	6	19	27	5.321928095
2017	6	20	31	6
2017	6	21	35	5.700439718
2017	6	22	24	6.247927513
2017	6	23	23	6.285402219
2017	6	24	25	6.700439718
2017	6	25	18	6.554588852
2017	6	26	21	6.044394119
2017	6	27	19	6.044394119
2017	6	28	16	5.754887502
2017	6	29	12	4.700439718
2017	6	30	11	4
2017	7	1	23	2.584962501
2017	7	2	13	1
2017	7	3	0	2.584962501
2017	7	7	26	1
2017	7	9	43	1
2017	7	10	45	2
2017	7	11	38	3
2017	7	12	61	4.700439718

2017	7	13	59	4.584962501
2017	7	14	52	4.807354922
2017	7	15	33	5.700439718
2017	7	16	21	5.906890596
2017	7	17	22	5
2017	7	18	0	3.584962501
2017	7	19	0	5.906890596
2017	7	20	0	6.357552005
2017	7	21	0	6.672425342
2017	7	22	0	6.491853096
2017	7	23	0	7
2017	7	24	0	7.108524457
2017	7	25	12	6.614709844
2017	7	26	12	6.392317423
2017	7	27	0	6.044394119
2017	7	28	11	5.64385619
2017	7	29	12	5
2017	7	30	15	4.321928095
2017	7	31	0	5.807354922
2017	8	1	13	4.700439718
2017	8	2	13	3.321928095
2017	8	3	13	4
2017	8	4	14	3.321928095
2017	8	5	13	3.321928095
2017	8	6	13	4.700439718
2017	8	7	12	3.807354922
2017	8	8	13	1
2017	8	12	13	3
2017	8	13	12	3.321928095
2017	8	14	13	2.584962501
2017	8	15	22	4.321928095
2017	8	16	33	4.459431619
2017	8	17	45	5.321928095
2017	8	18	45	4.807354922
2017	8	19	50	4.700439718
2017	8	20	60	4.700439718
2017	8	21	63	5.247927513
2017	8	22	63	5.754887502
2017	8	23	69	6.321928095
2017	8	24	54	6.672425342
2017	8	25	53	6.672425342
2017	8	26	48	6.584962501
2017	8	27	38	6.584962501
2017	8	28	23	6.426264755
2017	8	29	40	5.906890596
2017	8	30	51	5.906890596
2017	8	31	73	5.523561956
2017	9	1	59	3.807354922

2017	9	2	56	4.700439718
2017	9	3	105	3.321928095
2017	9	4	112	4.700439718
2017	9	5	119	3.584962501
2017	9	6	100	4.169925001
2017	9	7	97	3.584962501
2017	9	8	88	2
2017	9	9	62	2.584962501
2017	9	10	40	3.321928095
2017	9	11	29	3.807354922
2017	9	12	11	2.584962501
2017	9	13	23	1
2017	9	14	12	1
2017	9	16	13	3
2017	9	17	14	2
2017	9	18	13	2.584962501
2017	9	19	11	1

Year	Month	Date	Sunspot	log2(moths No.)+1	Shawan	Year	Month
1991	5	9	193	1		1995	6
1991	5	17	156	1		1995	6
1991	6	15	204	1		1995	7
1991	6	18	188	1		1995	7
1991	6	20	150	5.321928095		1995	7
1991	7	6	282	1		1995	7
1991	7	10	250	4.321928095		1995	7
1991	7	12	191	1		1995	7
1991	7	17	126	1		1995	7
1991	8	6	162	1		1995	7
1991	8	15	224	1		1996	5
1991	8	16	289	1		1996	5
1991	8	18	381	1		1996	5
1991	8	20	397	2.584962501		1996	6
1991	8	25	294	1		1996	6
1991	8	26	242	2.584962501		1996	6
1991	8	27	201	1		1996	6
1991	9	8	182	2.584962501		1996	6
1991	9	9	165	1		1996	6
1992	6	20	85	1		1996	6
1992	6	21	83	1		1996	6
1992	6	29	106	1		1996	6
1992	7	3	84	1		1996	6
1992	7	7	105	1		1996	7
1992	7	15	214	1		1996	7
1992	7	16	212	1		1996	7
1992	7	17	186	2		1996	7
1992	7	20	88	1		1996	7
1992	7	23	72	2.584962501		1996	7
1992	7	24	57	1		1996	7
1992	7	26	72	2		1996	7
1992	8	14	117	1		1996	7
1992	8	17	128	1		1996	8
1992	8	21	68	1		1996	8
1992	8	22	63	1		1996	8
1992	8	23	51	3		1996	8
1993	4	18	74	1		1996	8
1993	4	27	94	1		1996	8
1993	5	10	154	1		1996	8
1993	6	12	25	1		1996	8
1993	6	18	22	1		1996	8
1993	6	19	21	2		1996	8
1993	6	20	34	3		1996	8
1993	6	21	36	1		1996	8
1993	6	22	38	2		1996	8
1993	6	26	82	2		1996	8
1993	6	27	94	2		1996	8

1993	6	28	96	3	1996	8
1993	6	29	110	4	1996	8
1993	6	30	98	1	1997	5
1993	7	3	92	2	1997	5
1993	7	6	81	1	1997	5
1993	7	15	92	1	1997	5
1993	7	19	114	1	1997	5
1993	7	20	99	2	1997	5
1993	7	25	85	2	1997	5
1993	7	31	64	2	1997	5
1993	8	9	87	1	1997	5
1993	8	10	96	1	1997	5
1993	8	14	58	1	1997	5
1993	8	15	43	1	1997	5
1993	8	16	33	2	1997	5
1993	8	17	43	1	1997	5
1993	8	19	41	2	1997	5
1993	8	23	62	1	1997	6
1993	8	24	83	2	1997	6
1993	8	25	79	1	1997	6
1993	8	26	65	1	1997	6
1993	9	6	19	1	1997	6
1993	9	8	17	2.584962501	1997	6
1993	9	10	13	3	1997	6
1993	9	24	32	1	1997	6
1994	5	3	44	1	1997	6
1994	5	4	20	2	1997	6
1994	5	5	15	2.584962501	1997	6
1994	5	6	17	2.584962501	1997	6
1994	5	7	23	3.321928095	1997	6
1994	5	14	52	1	1997	6
1994	5	15	50	2	1997	7
1994	5	16	55	2	1997	7
1994	5	20	42	1	1997	7
1994	6	17	60	1	1997	7
1994	6	19	43	2.584962501	1997	7
1994	6	20	27	2.584962501	1997	7
1994	6	21	17	1	1997	7
1994	6	22	21	2	1997	7
1994	6	23	34	2.584962501	1997	7
1994	6	24	52	2	1997	7
1994	6	25	40	2	1997	7
1994	6	27	14	1	1997	7
1994	6	28	21	2.584962501	1997	7
1994	6	29	37	2	1997	7
1994	6	30	38	1	1997	7
1994	7	3	56	1	1997	7
1994	7	19	39	2	1997	7

1994	7	24	20	1	1997	7
1994	7	27	20	2	1997	7
1994	7	30	17	2	1997	7
1994	8	1	17	1	1997	7
1994	8	2	15	2	1997	7
1994	8	3	20	1	1997	7
1994	9	8	74	1	1997	8
1994	9	10	49	2	1997	8
1995	5	12	35	1	1997	8
1995	5	13	35	1	1997	8
1995	5	15	48	1	1997	8
1995	5	17	58	2	1997	8
1995	5	18	54	2.584962501	1997	8
1995	5	19	47	3.321928095	1997	8
1995	5	20	36	1	1997	8
1995	5	21	17	3	1997	8
1995	5	22	13	4	1997	8
1995	5	23	0	2	1997	8
1995	5	24	0	3	1997	8
1995	5	25	0	1	1997	8
1995	5	26	0	2	1997	8
1995	5	27	0	1	1997	8
1995	5	28	12	2.584962501	1997	8
1995	5	29	16	2	1997	8
1995	5	30	13	3.807354922	1997	8
1995	5	31	12	3.321928095	1997	8
1995	6	1	12	3.321928095	1997	8
1995	6	2	13	2.584962501	1997	8
1995	6	3	17	2	1997	8
1995	6	4	20	4.169925001	1997	8
1995	6	5	43	3.321928095	1997	8
1995	6	6	35	2	1997	8
1995	6	7	40	2.584962501	1997	8
1995	6	8	40	2.584962501	1997	8
1995	6	9	35	3	1997	8
1995	6	10	43	1	1997	8
1995	6	12	29	2	1997	9
1995	6	13	24	1	1997	9
1995	6	14	17	2	1997	9
1995	6	16	0	3	1997	9
1995	6	17	10	2.584962501	1997	9
1995	6	18	13	3.321928095	1997	9
1995	6	19	26	2	1998	5
1995	6	21	22	3	1998	5
1995	6	22	24	2.584962501	1998	5
1995	6	23	23	4.700439718	1998	5
1995	6	24	19	3.321928095	1998	5
1995	6	25	19	3	1998	5

1995	6	26	17	2	1998	6
1995	6	27	14	3.321928095	1998	6
1995	6	28	13	3.321928095	1998	6
1995	6	29	20	2.584962501	1998	6
1995	6	30	33	1	1998	6
1995	7	1	42	3	1998	6
1995	7	2	35	3.807354922	1998	6
1995	7	3	20	2.584962501	1998	6
1995	7	6	38	3	1998	6
1995	7	7	41	2	1998	6
1995	7	8	35	2.584962501	1998	6
1995	7	9	39	3.321928095	1998	6
1995	7	10	30	2.584962501	1998	7
1995	7	11	25	3.584962501	1998	7
1995	7	12	18	2.584962501	1998	7
1995	7	13	15	1	1998	7
1995	7	14	18	2	1998	7
1995	7	15	27	2	1998	7
1995	7	16	24	2.584962501	1998	7
1995	7	17	22	3.321928095	1998	7
1995	7	18	20	3.321928095	1998	7
1995	7	19	30	1	1998	7
1995	7	20	32	3	1998	7
1995	7	21	20	1	1998	7
1995	7	22	15	2	1998	7
1995	7	23	0	2	1998	7
1995	7	26	10	1	1998	7
1995	7	28	0	2	1998	7
1995	7	29	0	2.584962501	1998	7
1995	7	31	14	1	1998	7
1995	8	1	18	1	1998	7
1995	8	3	22	2	1998	7
1995	8	6	28	1	1998	7
1995	8	15	15	1	1998	8
1995	8	17	17	1	1998	8
1995	9	8	0	2	1998	8
1995	9	15	13	1	1998	8
1995	9	23	38	1	1998	8
1995	9	26	33	1	1998	8
1995	9	29	12	2	1998	8
1996	5	3	0	2	1998	8
1996	5	6	15	1	1998	8
1996	5	16	13	2	1998	8
1996	5	17	11	1	1998	8
1996	5	18	11	1	1998	8
1996	5	19	0	3	1998	8
1996	5	20	11	2	1998	8
1996	5	25	0	1	1998	8

1996	5	26	0	2		1998	8
1996	5	27	0	1		1998	8
1996	5	30	0	1		1998	8
1996	6	11	13	2		1998	8
1996	6	13	0	1		1998	8
1996	6	26	25	1		1998	8
1996	6	27	25	3		1998	8
1996	6	28	25	1		1998	8
1996	7	2	14	1		1998	8
1996	7	4	0	2		1998	8
1996	7	5	12	2		1998	8
1996	7	10	37	2		1998	8
1996	7	11	29	3.321928095		1998	8
1996	7	13	13	1		1998	8
1996	7	15	0	1		1998	9
1996	7	16	0	3.321928095		1998	9
1996	7	17	0	2		1998	9
1996	7	19	0	1		1998	9
1996	7	21	0	3		1998	9
1996	7	22	0	3		1998	9
1996	7	25	0	1		1998	9
1996	7	30	27	1		1998	9
1996	8	5	22	1		1998	9
1996	8	7	23	2		1998	9
1996	8	8	15	1		1998	9
1996	8	10	20	2		1998	9
1996	8	13	16	2		1998	9
1996	8	14	29	1		1998	9
1996	8	17	15	1		1998	9
1996	8	20	14	1		1999	5
1996	8	25	14	2		1999	5
1996	8	27	18	1		1999	5
1996	8	29	15	1		1999	5
1996	8	31	26	2.584962501		1999	5
1997	4	18	13	1		1999	5
1997	4	19	16	1		1999	5
1997	4	20	0	2		1999	5
1997	4	21	0	1		1999	5
1997	4	24	13	1		1999	5
1997	4	27	16	2		1999	5
1997	4	28	18	2.584962501		1999	5
1997	4	29	12	2		1999	6
1997	5	1	0	1		1999	6
1997	5	2	0	1		1999	6
1997	5	5	12	2		1999	7
1997	5	6	11	2		1999	7
1997	5	7	11	1		1999	7
1997	5	8	14	4.459431619		1999	7

1997	5	13	17	1	1999	7
1997	5	16	23	2	1999	7
1997	5	17	23	1	1999	7
1997	5	27	34	1	1999	7
1997	5	28	23	2	1999	7
1997	5	29	19	2	1999	7
1997	5	30	17	1	1999	7
1997	6	7	20	1	1999	7
1997	6	8	31	2.584962501	1999	7
1997	6	9	26	2	1999	7
1997	6	10	13	1	1999	7
1997	6	11	13	3	1999	7
1997	6	12	26	2	1999	7
1997	6	13	29	2	1999	8
1997	6	14	31	2	1999	8
1997	6	15	31	2.584962501	1999	8
1997	6	16	33	2	1999	8
1997	6	17	21	1	1999	8
1997	6	18	29	2.584962501	1999	8
1997	6	19	16	4.169925001	1999	8
1997	6	20	13	3.584962501	1999	8
1997	6	21	11	3	1999	8
1997	6	22	0	2.584962501	1999	8
1997	6	23	15	2	1999	8
1997	6	24	15	2.584962501	1999	8
1997	6	25	31	2	1999	8
1997	6	26	24	1	1999	8
1997	6	27	20	1	1999	8
1997	6	28	13	1	1999	8
1997	6	29	13	1	1999	8
1997	7	5	10	2	1999	8
1997	7	6	12	2.584962501	1999	8
1997	7	8	20	2.584962501	1999	8
1997	7	9	21	3.321928095	1999	8
1997	7	10	16	3.321928095	1999	8
1997	7	11	0	1	1999	8
1997	7	12	0	1	1999	8
1997	7	13	0	1	1999	9
1997	7	18	9	1	1999	9
1997	7	23	25	1	1999	9
1997	7	24	36	2	1999	9
1997	7	25	48	3	1999	9
1997	7	26	42	1	1999	9
1997	7	27	31	2	1999	9
1997	7	29	14	2.584962501	1999	9
1997	7	30	0	2	1999	9
1997	7	31	0	2	2000	5
1997	8	3	19	2	2000	5

1997	8	4	13	1	2000	5
1997	8	5	25	2	2000	5
1997	8	6	53	1	2000	5
1997	8	7	60	2.584962501	2000	5
1997	8	8	66	1	2000	5
1997	8	9	56	1	2000	5
1997	8	10	31	2	2000	5
1997	8	11	53	1	2000	5
1997	8	12	70	2	2000	6
1997	8	13	67	1	2000	6
1997	8	14	57	2.584962501	2000	6
1997	8	15	54	1	2000	6
1997	8	16	31	1	2000	6
1997	8	18	23	2	2000	6
1997	8	19	13	1	2000	6
1997	8	20	12	1	2000	6
1997	8	21	13	2	2000	6
1997	8	22	12	1	2000	6
1997	8	23	13	1	2000	6
1997	9	5	58	1	2000	7
1997	9	6	65	2	2000	7
1997	9	8	101	1	2000	7
1997	9	9	106	1	2000	7
1997	9	10	108	2.584962501	2000	7
1997	9	12	103	1	2000	7
1997	9	13	99	1	2000	7
1997	9	14	82	1	2000	7
1997	9	15	76	2	2000	7
1997	9	16	71	1	2000	7
1997	9	17	57	2	2000	7
1997	9	18	38	1	2000	7
1997	9	19	19	1	2000	7
1997	9	23	49	2	2000	7
1997	9	25	57	1	2000	7
1997	9	28	21	1	2000	7
1997	9	30	22	1	2000	7
1998	4	21	45	1	2000	7
1998	4	26	17	1	2000	7
1998	4	27	16	1	2000	7
1998	4	29	48	2	2000	7
1998	5	2	96	2	2000	7
1998	5	5	104	1	2000	7
1998	5	7	93	2	2000	7
1998	5	14	108	1	2000	7
1998	5	17	93	1	2000	7
1998	5	18	88	2	2000	8
1998	5	19	74	2.584962501	2000	8
1998	5	20	57	2	2000	8

1998	5	21	34	1	2000	8
1998	5	24	42	1	2000	8
1998	6	11	97	1	2000	8
1998	6	15	68	1	2000	8
1998	6	19	67	1	2000	8
1998	6	22	65	1	2000	8
1998	6	23	68	1	2000	8
1998	6	24	58	3	2000	8
1998	6	25	96	2	2000	8
1998	6	26	106	1	2000	8
1998	6	27	128	1	2000	8
1998	6	28	138	1	2000	8
1998	7	5	137	2	2000	8
1998	7	6	107	1	2000	8
1998	7	7	74	2.584962501	2000	8
1998	7	8	55	1	2000	8
1998	7	9	46	1	2000	8
1998	7	10	71	1	2000	8
1998	7	19	51	1	2000	8
1998	7	31	99	1	2000	8
1998	8	3	89	3.584962501	2000	8
1998	8	14	123	1	2000	8
1998	8	15	96	3.807354922	2000	9
1998	8	16	123	3.321928095	2001	5
1998	8	17	114	3.584962501	2001	6
1998	8	18	109	3	2001	6
1998	8	19	133	2	2001	6
1998	8	20	120	2	2001	6
1998	8	21	117	3	2001	6
1998	8	22	106	1	2001	6
1998	8	24	104	1	2001	6
1998	8	29	134	1	2001	6
1998	8	31	143	1	2001	6
1998	9	11	136	1	2001	6
1998	9	12	131	2.584962501	2001	6
1998	9	13	135	2	2001	6
1998	9	14	111	1	2001	6
1998	9	15	85	2	2001	6
1998	9	16	80	1	2001	6
1999	4	21	62	2.584962501	2001	6
1999	4	22	59	2.584962501	2001	6
1999	4	24	75	1	2001	6
1999	4	25	66	1	2001	6
1999	4	29	90	2	2001	6
1999	4	30	97	1	2001	6
1999	5	2	129	1	2001	7
1999	5	3	112	1	2001	7
1999	5	5	124	1	2001	7

1999	5	6	146	1	2001	7
1999	5	7	200	1	2001	7
1999	5	8	214	1	2001	7
1999	5	10	191	2	2001	7
1999	5	12	172	1	2001	7
1999	5	13	142	1	2001	7
1999	5	15	148	1	2001	7
1999	5	16	148	2	2001	7
1999	5	17	139	1	2001	7
1999	5	18	131	1	2001	7
1999	5	19	132	2	2001	7
1999	5	20	135	1	2001	7
1999	5	21	153	1	2001	8
1999	5	22	138	2	2001	8
1999	5	23	121	1	2001	8
1999	6	14	221	1	2001	8
1999	6	15	209	1	2001	8
1999	6	16	181	2	2001	8
1999	6	17	146	2.584962501	2001	8
1999	6	18	120	2.584962501	2001	8
1999	6	19	119	3.321928095	2001	8
1999	6	20	93	4.169925001	2001	8
1999	6	21	119	3	2001	8
1999	6	22	160	3	2001	8
1999	6	23	217	3.584962501	2001	8
1999	6	24	293	3.321928095	2001	8
1999	6	25	292	3	2001	8
1999	6	26	274	2	2001	8
1999	6	27	259	2.584962501	2001	8
1999	6	28	254	2.584962501	2001	8
1999	6	29	241	2.584962501	2001	8
1999	6	30	223	2	2001	8
1999	7	1	215	2	2001	8
1999	7	2	223	2	2001	9
1999	7	3	217	2.584962501	2001	9
1999	7	4	205	2	2002	6
1999	7	5	199	2	2002	6
1999	7	6	186	3	2002	6
1999	7	7	179	2	2002	6
1999	7	8	173	2.584962501	2002	6
1999	7	9	176	2	2002	6
1999	7	10	171	2	2002	6
1999	7	11	176	1	2002	6
1999	7	12	199	2	2002	6
1999	7	13	157	1	2002	6
1999	7	14	128	1	2002	6
1999	7	15	122	1	2002	6
1999	7	16	118	1	2002	6

1999	7	17	121	2	2002	6
1999	7	18	137	1	2002	6
1999	7	19	121	1	2002	6
1999	7	30	252	2.584962501	2002	6
1999	7	31	223	3.321928095	2002	6
1999	8	1	252	3	2002	6
1999	8	2	250	1	2002	7
1999	8	3	229	3	2002	7
1999	8	4	193	2.584962501	2002	7
1999	8	5	193	4.584962501	2002	7
1999	8	6	167	3	2002	7
1999	8	7	152	3	2002	7
1999	8	8	149	2.584962501	2002	7
1999	8	9	115	5.392317423	2002	7
1999	8	10	82	6.044394119	2002	7
1999	8	11	79	6.129283017	2002	7
1999	8	12	91	2	2002	7
1999	8	13	102	2	2002	7
1999	8	14	87	2	2002	7
1999	8	15	74	1	2002	8
1999	8	16	67	3.321928095	2002	8
1999	8	17	55	2	2002	8
1999	8	18	58	2	2002	8
1999	8	19	64	1	2002	8
1999	8	20	73	2	2002	8
1999	8	21	88	1	2002	8
1999	8	22	103	2	2002	8
1999	8	23	115	2	2002	8
1999	8	24	131	1	2002	8
1999	8	26	206	1	2002	8
1999	8	27	194	1	2002	8
1999	8	28	223	2	2002	8
1999	8	29	231	2	2002	8
1999	8	30	228	1	2002	8
1999	8	31	165	2	2002	8
1999	9	1	140	1	2002	9
1999	9	2	122	1	2002	9
1999	9	3	115	1	2002	9
2000	4	27	180	2	2002	9
2000	4	28	189	1	2002	9
2000	4	30	153	1	2002	9
2000	5	1	124	2.584962501	2002	9
2000	5	2	109	1	2002	9
2000	5	3	104	1	2002	9
2000	5	4	97	1	2002	9
2000	5	5	97	1	2002	9
2000	5	9	135	2.584962501	2002	9
2000	5	19	228	1	2002	9

2000	5	21	222	2.584962501	2002	9
2000	6	8	181	1	2002	9
2000	6	9	184	1	2002	9
2000	6	10	179	2	2002	9
2000	6	11	227	1	2003	5
2000	6	12	221	3	2003	5
2000	6	13	235	1	2003	5
2000	6	14	257	2	2003	5
2000	6	15	238	4.321928095	2003	5
2000	6	16	214	4.321928095	2003	6
2000	6	17	209	5.087462841	2003	6
2000	6	18	221	5.087462841	2003	6
2000	6	19	218	6	2003	6
2000	6	20	239	5.754887502	2003	6
2000	6	21	221	7.129283017	2003	6
2000	6	22	191	2.584962501	2003	6
2000	6	23	187	3.584962501	2003	6
2000	6	24	179	4.169925001	2003	6
2000	6	25	167	4.321928095	2003	6
2000	6	26	194	3.584962501	2003	6
2000	6	27	208	2.584962501	2003	6
2000	6	28	173	3.584962501	2003	6
2000	6	29	164	2.584962501	2003	6
2000	6	30	172	2.584962501	2003	6
2000	7	1	208	2.584962501	2003	6
2000	7	2	202	3	2003	6
2000	7	3	178	3	2003	6
2000	7	4	164	1	2003	7
2000	7	7	254	1	2003	7
2000	7	9	257	1	2003	7
2000	7	22	310	3.321928095	2003	8
2000	7	23	286	3.584962501	2003	8
2000	7	25	254	2.584962501	2003	8
2000	7	28	172	1	2003	8
2000	7	29	162	2	2003	8
2000	7	30	161	3.321928095	2003	8
2000	7	31	134	2.584962501	2003	8
2000	8	2	152	3	2003	8
2000	8	5	199	2	2003	8
2000	8	6	198	2	2003	8
2000	8	7	227	2	2003	8
2000	8	8	194	1	2003	9
2000	8	10	213	2.584962501	2003	9
2000	8	11	228	2	2003	9
2000	8	12	235	1	2003	9
2000	8	13	243	1	2003	9
2000	8	16	246	1	2003	9
2000	8	17	210	2	2003	9

2000	8	18	194	1	2003	9
2000	8	19	184	2	2004	5
2000	8	20	147	3	2004	5
2000	8	21	106	1	2004	6
2000	8	24	106	1	2004	6
2000	8	26	109	1	2004	6
2000	8	27	156	4.321928095	2004	6
2000	8	28	183	2	2004	6
2000	9	5	162	2	2004	6
2001	4	17	42	1	2004	6
2001	4	24	164	1	2004	6
2001	4	27	192	1	2004	6
2001	4	30	168	1	2004	6
2001	5	18	137	2.584962501	2004	6
2001	5	20	121	1	2004	7
2001	5	23	197	1	2004	7
2001	5	30	110	1	2004	7
2001	6	4	145	1	2004	7
2001	6	5	161	1	2004	7
2001	6	9	254	1	2004	8
2001	6	10	241	2	2004	8
2001	6	12	259	2.584962501	2004	8
2001	6	13	242	3	2004	8
2001	6	14	273	4	2004	8
2001	6	15	282	3.584962501	2004	8
2001	6	16	289	2.584962501	2004	8
2001	6	17	270	1	2004	8
2001	6	18	232	1	2004	8
2001	6	19	214	3.321928095	2004	8
2001	6	20	206	3.807354922	2004	8
2001	6	21	218	3.807354922	2004	8
2001	6	22	229	2.584962501	2004	8
2001	6	23	235	2.584962501	2004	8
2001	6	24	220	2	2004	8
2001	6	25	198	1	2004	8
2001	6	27	162	2	2004	8
2001	6	29	112	1	2004	8
2001	6	30	98	1	2004	8
2001	7	1	111	1	2004	8
2001	7	2	125	1	2004	8
2001	7	5	93	1	2004	8
2001	7	12	135	1	2004	8
2001	7	13	167	1	2004	8
2001	7	19	183	1	2004	8
2001	7	20	177	1	2004	8
2001	7	21	144	1	2004	8
2001	7	22	150	1	2004	8
2001	7	23	152	2	2004	8

2001	7	25	119	2	2004	9
2001	7	26	92	1	2004	9
2001	7	27	90	1	2004	9
2001	7	31	78	1	2004	9
2001	8	8	178	1	2004	9
2001	8	10	150	2	2004	9
2001	8	12	170	1	2004	9
2001	8	16	193	2	2004	9
2001	8	18	161	3.584962501	2004	9
2001	8	24	176	1	2004	9
2001	8	26	153	2	2004	9
2002	4	25	228	1	2004	9
2002	5	4	258	2	2004	9
2002	5	8	220	1	2004	9
2002	5	12	202	2	2004	9
2002	5	13	161	2	2004	9
2002	5	20	166	2	2004	9
2002	6	2	188	2	2004	9
2002	6	4	219	1	2004	9
2002	6	8	185	2.584962501	2004	9
2002	6	9	165	2.584962501	2004	9
2002	6	10	128	2	2004	9
2002	6	11	99	3.321928095	2004	9
2002	6	12	109	1	2004	9
2002	6	13	80	4	2004	9
2002	6	14	107	3.807354922	2004	9
2002	6	15	102	4.459431619	2004	9
2002	6	16	117	4.807354922	2005	5
2002	6	17	88	3.321928095	2005	5
2002	6	18	127	4.906890596	2005	5
2002	6	19	115	1	2005	6
2002	6	20	108	2.584962501	2005	6
2002	7	17	147	1	2005	6
2002	7	26	265	1	2005	6
2002	7	30	281	2.584962501	2005	6
2002	7	31	239	2	2005	6
2002	8	1	207	2	2005	6
2002	8	2	199	2.584962501	2005	6
2002	8	3	183	3.321928095	2005	6
2002	8	4	143	3.321928095	2005	6
2002	8	5	127	3	2005	6
2002	8	6	131	4.169925001	2005	6
2002	8	7	133	7.475733431	2005	6
2002	8	8	115	5.247927513	2005	6
2002	8	9	110	8.599912842	2005	6
2002	8	10	110	7.87036472	2005	6
2002	8	11	149	8.169925001	2005	6
2002	8	12	177	3	2005	7

2002	8	13	202	7.614709844	2005	7
2002	8	14	267	7.108524457	2005	7
2002	8	15	279	3.807354922	2005	7
2002	8	16	263	3.584962501	2005	7
2002	8	17	281	3	2005	7
2002	8	18	270	3.321928095	2005	7
2002	8	19	248	3.584962501	2005	7
2002	8	20	211	1	2005	7
2002	8	22	172	1	2005	7
2002	8	26	119	1	2005	7
2002	8	28	122	3.584962501	2005	7
2002	8	31	160	1	2005	7
2002	9	1	206	4.807354922	2005	7
2002	9	2	233	5.087462841	2005	7
2002	9	3	252	4.169925001	2005	7
2002	9	4	247	2	2005	7
2002	9	5	226	1	2005	7
2002	9	6	202	1	2005	7
2002	9	7	206	2.584962501	2005	7
2002	9	8	213	3.584962501	2005	7
2002	9	9	199	3.807354922	2005	7
2002	9	10	202	2.584962501	2005	7
2002	9	11	187	2	2005	7
2002	9	12	187	2	2005	8
2002	9	16	170	2	2005	8
2002	9	22	185	1	2005	8
2003	4	24	119	2	2005	8
2003	4	29	178	2.584962501	2005	8
2003	4	30	160	1	2005	8
2003	5	2	137	1	2005	8
2003	5	11	57	1	2005	8
2003	5	13	65	1	2005	8
2003	5	26	83	1	2005	8
2003	6	11	178	1	2005	8
2003	6	17	86	1	2005	8
2003	6	18	104	2.584962501	2005	8
2003	6	19	117	1	2005	8
2003	6	22	94	1	2005	8
2003	6	23	101	3	2005	8
2003	6	24	104	4.169925001	2005	8
2003	6	26	126	2.584962501	2005	8
2003	6	28	143	2.584962501	2005	8
2003	6	29	144	2	2005	8
2003	6	30	141	2	2005	8
2003	7	1	154	3.321928095	2005	8
2003	7	2	149	2	2005	8
2003	7	4	103	4.584962501	2005	8
2003	7	5	86	4	2005	8

2003	7	6	97	3	2005	8
2003	7	7	131	1	2005	8
2003	7	8	137	1	2005	8
2003	7	9	139	1	2005	9
2003	7	10	114	1	2005	9
2003	7	11	94	1	2005	9
2003	7	12	105	1	2005	9
2003	7	13	148	1	2005	9
2003	7	14	148	1	2005	9
2003	7	22	190	2.584962501	2005	9
2003	7	23	154	2	2005	9
2003	7	25	72	1	2006	5
2003	7	28	77	1	2006	5
2003	7	30	59	2	2006	5
2003	7	31	65	4.321928095	2006	5
2003	8	1	78	3	2006	5
2003	8	2	89	3.584962501	2006	5
2003	8	3	121	1	2006	5
2003	8	4	116	2.584962501	2006	5
2003	8	5	138	1	2006	5
2003	8	6	132	4.700439718	2006	5
2003	8	8	109	1	2006	6
2003	8	17	117	2.584962501	2006	6
2003	8	18	106	2	2006	6
2003	8	19	92	1	2006	6
2003	8	20	98	2.584962501	2006	6
2003	8	21	92	5.247927513	2006	6
2003	8	22	109	3	2006	6
2003	8	23	121	4.906890596	2006	6
2003	8	24	130	4.321928095	2006	6
2003	8	25	130	3.807354922	2006	6
2003	8	27	143	3.807354922	2006	6
2003	8	29	135	4	2006	6
2003	8	30	117	4.321928095	2006	6
2003	8	31	103	3.584962501	2006	6
2003	9	1	74	3	2006	6
2003	9	2	74	1	2006	6
2003	9	3	76	2	2006	7
2003	9	4	81	2.584962501	2006	7
2003	9	5	63	3	2006	7
2003	9	6	60	2	2006	7
2003	9	7	48	1	2006	7
2003	9	12	47	2	2006	7
2003	9	16	74	2.584962501	2006	7
2003	9	18	94	1	2006	7
2003	9	19	84	2	2006	7
2003	9	20	74	3	2006	7
2003	9	21	81	2.584962501	2006	7

2003	9	22	92	2	2006	7
2003	9	24	103	1	2006	7
2003	9	25	108	1	2006	7
2003	9	26	124	2	2006	7
2004	4	17	75	1	2006	7
2004	4	21	89	1	2006	7
2004	4	22	86	1	2006	7
2004	5	7	28	2	2006	7
2004	5	13	65	1	2006	8
2004	6	16	98	1	2006	8
2004	6	19	106	1	2006	8
2004	6	20	132	2.584962501	2006	8
2004	6	21	134	1	2006	8
2004	7	17	130	1	2006	8
2004	7	20	149	2	2006	8
2004	7	21	144	1	2006	8
2004	7	25	94	1	2006	8
2004	8	4	56	1	2006	8
2004	8	5	36	1	2006	8
2004	8	7	75	2	2006	8
2004	8	8	67	2	2006	8
2004	8	9	85	1	2006	8
2004	8	10	99	2	2006	8
2004	8	11	107	1	2006	8
2004	8	12	116	1	2006	8
2004	8	13	130	3	2006	8
2004	8	14	116	1	2006	8
2004	8	15	104	2	2006	8
2004	8	16	92	2.584962501	2006	8
2004	8	17	75	2	2006	8
2004	8	18	70	3.584962501	2006	8
2004	8	19	61	1	2006	8
2004	8	21	97	1	2006	8
2004	8	25	53	1	2006	8
2004	8	26	41	1	2006	8
2004	8	27	36	1	2006	8
2004	8	28	34	1	2006	8
2004	8	29	27	1	2006	8
2004	9	3	32	1	2006	9
2004	9	14	56	1	2006	9
2004	9	16	67	2	2006	9
2004	9	19	60	1	2006	9
2004	9	21	42	1	2006	9
2004	9	25	18	1	2007	5
2004	9	26	26	1	2007	5
2004	10	6	26	1	2007	5
2005	4	21	26	1	2007	5
2005	4	26	18	1	2007	5

2005	4	29	56	1	2007	5
2005	5	1	61	2	2007	5
2005	5	2	61	1	2007	5
2005	5	3	68	1	2007	5
2005	5	4	62	3.321928095	2007	5
2005	5	5	58	2	2007	5
2005	5	6	65	1	2007	5
2005	5	8	67	1	2007	5
2005	5	10	111	3	2007	5
2005	5	11	119	2	2007	5
2005	7	28	28	1	2007	5
2005	7	29	53	2	2007	5
2005	7	30	67	2.584962501	2007	5
2005	7	31	107	3.807354922	2007	6
2005	8	3	80	1	2007	6
2005	8	4	66	2	2007	6
2005	8	8	61	1	2007	6
2005	8	9	57	1	2007	6
2005	8	11	37	3	2007	6
2005	8	15	45	2	2007	6
2005	8	16	40	1	2007	6
2005	8	20	80	1	2007	6
2005	8	30	61	2.584962501	2007	6
2005	8	31	48	2	2007	6
2005	9	1	29	1	2007	6
2005	9	7	24	3.807354922	2007	6
2005	9	8	34	3	2007	6
2005	9	9	48	1	2007	6
2005	9	10	59	2	2007	6
2005	9	11	58	1	2007	7
2005	9	12	63	1	2007	7
2005	9	13	85	2.584962501	2007	7
2005	9	21	22	1	2007	7
2005	9	23	32	1	2007	7
2005	9	25	27	1	2007	7
2005	9	28	25	1	2007	7
2005	9	29	24	1	2007	7
2005	9	30	12	1	2007	7
2006	4	25	47	1	2007	7
2006	4	26	58	1	2007	7
2006	5	1	55	1	2007	7
2006	5	19	13	1	2007	7
2006	5	27	53	1	2007	7
2006	5	31	45	1	2007	7
2006	6	22	0	1	2007	7
2006	6	30	44	1	2007	7
2006	7	1	38	1	2007	7
2006	7	15	16	1	2007	7

2006	7	29	16	1	2007	7
2006	7	30	31	2	2007	7
2006	7	31	27	2	2007	8
2006	8	1	11	2	2007	8
2006	8	2	11	1	2007	8
2006	8	4	0	1	2007	8
2006	8	5	0	1	2007	8
2006	8	13	26	2.584962501	2007	8
2006	8	14	31	4.584962501	2007	8
2006	8	25	24	1	2007	8
2006	8	26	23	1	2007	8
2006	8	27	34	1	2007	8
2006	8	28	35	4	2007	8
2006	8	29	27	2	2007	8
2006	8	30	19	2.584962501	2007	8
2006	9	1	35	4.807354922	2007	8
2006	9	2	16	5	2007	8
2006	9	3	0	4	2007	8
2006	9	4	0	4	2007	8
2006	9	7	48	2	2007	8
2006	9	10	49	4	2007	8
2006	9	11	44	2	2007	8
2006	9	15	15	2	2007	8
2006	9	17	25	2	2007	8
2006	9	18	12	1	2007	8
2006	9	20	13	3.807354922	2007	8
2006	9	22	18	2	2007	8
2006	10	3	23	1	2007	8
2007	4	19	0	1	2007	8
2007	4	23	0	2	2007	8
2007	4	25	13	1	2007	8
2007	5	1	33	1	2007	9
2007	5	3	20	1	2007	9
2007	5	5	17	2.584962501	2007	9
2007	5	6	15	2	2007	9
2007	5	15	30	1	2007	9
2007	5	16	32	1	2007	9
2007	5	18	42	2	2007	9
2007	5	19	37	2.584962501	2007	9
2007	6	13	14	1	2007	9
2007	6	14	0	3.584962501	2007	9
2007	6	15	0	1	2007	9
2007	6	18	0	2.584962501	2007	9
2007	6	24	0	1	2007	9
2007	6	25	12	2	2007	9
2007	6	28	16	2	2007	9
2007	6	29	32	2.584962501	2007	9
2007	7	1	30	1	2007	10

2007	7	5	14	1	2007	10
2007	7	7	12	1	2007	10
2007	7	11	27	1	2007	10
2007	7	12	23	1	2008	5
2007	7	17	17	2	2008	5
2007	7	30	14	1	2008	5
2007	7	31	0	2	2008	5
2007	8	4	11	3	2008	5
2007	8	5	11	2.584962501	2008	5
2007	8	6	13	2.584962501	2008	5
2007	8	7	13	2	2008	5
2007	8	8	15	2.584962501	2008	5
2007	8	9	15	2.584962501	2008	5
2007	8	10	15	3	2008	5
2007	8	11	13	3.807354922	2008	5
2007	8	12	13	3.321928095	2008	5
2007	8	13	13	2	2008	5
2007	8	14	0	2.584962501	2008	5
2007	8	23	13	1	2008	5
2007	8	24	13	1	2008	5
2007	8	25	13	1	2008	5
2008	4	28	0	1	2008	5
2008	4	29	0	1	2008	6
2008	4	30	0	1	2008	6
2008	5	1	10	2	2008	6
2008	5	2	0	2	2008	6
2008	5	3	0	1	2008	6
2008	5	4	10	1	2008	6
2008	5	6	0	2	2008	6
2008	5	7	0	1	2008	6
2008	5	10	0	1	2008	6
2008	5	11	0	1	2008	6
2008	5	16	20	1	2008	6
2008	5	18	22	1	2008	6
2008	5	21	0	2	2008	6
2008	5	26	0	3	2008	6
2008	5	27	0	1	2008	6
2008	5	30	0	1	2008	6
2008	5	31	0	1	2008	6
2008	6	2	0	2	2008	6
2008	6	6	0	2	2008	6
2008	6	12	11	1	2008	6
2008	6	13	12	2	2008	6
2008	6	14	0	2	2008	6
2008	6	17	11	1	2008	6
2008	6	18	12	2	2008	6
2008	6	19	11	2.584962501	2008	6
2008	6	20	12	1	2008	6

2008	6	23	0	3	2008	6
2008	6	25	0	1	2008	6
2008	6	26	0	3.807354922	2008	6
2008	6	27	0	5.247927513	2008	6
2008	6	28	0	2	2008	7
2008	6	29	0	2	2008	7
2008	6	30	0	2	2008	7
2008	7	3	0	4.459431619	2008	7
2008	7	6	0	2	2008	7
2008	7	7	0	3.584962501	2008	7
2008	7	8	0	2	2008	7
2008	7	9	0	4	2008	7
2008	7	10	0	3.321928095	2008	7
2008	7	11	0	1	2008	7
2008	7	12	0	1	2008	7
2008	7	14	0	2	2008	7
2008	7	16	0	1	2008	7
2008	7	18	6	2.584962501	2008	7
2008	7	24	0	2	2008	7
2008	8	1	0	4.459431619	2008	7
2008	8	2	0	4.321928095	2008	7
2008	8	3	0	1	2008	7
2008	8	6	0	5.321928095	2008	7
2008	8	7	0	3.807354922	2008	7
2008	8	8	0	2.584962501	2008	7
2008	8	9	0	2.584962501	2008	7
2008	8	10	0	4	2008	7
2008	8	11	0	4	2008	7
2008	8	12	0	4.584962501	2008	7
2008	8	13	0	4	2008	7
2008	8	14	0	2.584962501	2008	7
2008	8	15	0	2	2008	7
2008	8	16	0	4.700439718	2008	7
2008	8	17	0	1	2008	8
2008	8	20	0	1	2008	8
2008	8	21	5	3.321928095	2008	8
2008	8	22	5	1	2008	8
2008	8	23	0	1	2008	8
2008	8	25	0	2	2008	8
2008	8	26	0	1	2008	8
2008	8	28	0	1	2008	8
2008	8	29	0	2	2008	8
2008	8	30	0	2.584962501	2008	8
2008	9	1	0	3.321928095	2008	8
2008	9	2	0	4.169925001	2008	8
2008	9	3	0	1	2008	8
2008	9	4	0	2.584962501	2008	8
2008	9	5	0	5.700439718	2008	8

2008	9	8	0	1	2008	8
2009	4	16	0	1	2008	8
2009	4	22	0	1	2008	8
2009	4	23	0	2	2008	8
2009	4	24	0	1	2008	8
2009	4	27	0	1	2008	8
2009	4	28	0	1	2008	8
2009	4	30	13	1	2008	8
2009	5	22	8	1	2008	8
2009	6	12	0	1	2008	8
2009	6	14	0	2	2008	8
2009	6	15	0	2.584962501	2008	8
2009	6	16	0	2.584962501	2008	8
2009	6	17	15	3.584962501	2008	8
2009	6	18	0	3.321928095	2008	8
2009	6	19	0	3.807354922	2008	8
2009	6	20	0	3.584962501	2008	9
2009	6	21	15	3.321928095	2008	9
2009	6	22	17	3	2008	9
2009	6	23	15	2.584962501	2008	9
2009	6	24	17	2.584962501	2008	9
2009	6	25	0	3	2008	9
2009	6	26	0	1	2008	9
2009	6	28	0	1	2008	9
2009	7	1	0	1	2008	9
2009	7	2	0	2	2008	9
2009	7	4	24	1	2008	9
2009	7	12	0	1	2008	9
2009	7	20	0	2	2008	9
2009	7	21	0	2	2008	9
2009	7	25	0	1	2008	9
2009	7	28	0	3.584962501	2008	9
2009	7	31	0	1	2008	9
2009	8	2	0	1	2008	9
2009	8	3	0	1	2009	5
2009	8	5	0	1	2009	5
2009	8	6	0	2	2009	5
2009	8	15	0	1	2009	5
2009	8	16	0	1	2009	5
2009	8	24	0	1	2009	5
2009	8	25	0	2	2009	5
2009	8	30	0	1	2009	5
2009	9	1	13	1	2009	5
2009	9	3	0	1	2009	5
2009	9	4	0	1	2009	5
2009	9	5	0	2	2009	5
2009	9	9	0	1	2009	5
2009	9	12	0	2.584962501	2009	5

2009	9	13	0	1	2009	5
2009	9	18	0	1	2009	5
2010	4	24	0	1	2009	5
2010	4	26	0	1	2009	5
2010	4	29	0	2	2009	6
2010	5	2	21	1	2009	6
2010	5	4	51	1	2009	6
2010	5	7	16	1	2009	6
2010	5	14	0	1	2009	6
2010	5	15	0	1	2009	6
2010	5	18	0	1	2009	6
2010	5	24	19	1	2009	6
2010	5	25	24	1	2009	6
2010	6	4	28	1	2009	6
2010	6	6	12	1	2009	6
2010	6	9	29	1	2009	6
2010	6	14	14	1	2009	6
2010	6	19	26	1	2009	6
2010	6	20	18	1	2009	6
2010	6	23	14	1	2009	6
2010	6	24	12	1	2009	6
2010	6	25	17	2	2009	6
2010	6	26	18	1	2009	6
2010	6	28	11	1	2009	6
2010	6	30	11	1	2009	6
2010	7	1	13	1	2009	6
2010	7	2	13	2	2009	7
2010	7	4	14	1	2009	7
2010	7	7	13	1	2009	7
2010	7	11	36	1	2009	7
2010	7	12	34	1	2009	7
2010	7	13	30	1	2009	7
2010	7	16	16	1	2009	7
2010	7	18	14	2.584962501	2009	7
2010	7	20	30	1	2009	7
2010	7	21	31	1	2009	7
2010	7	23	44	1	2009	7
2010	7	26	33	1	2009	7
2010	7	28	34	1	2009	7
2010	7	29	38	2.584962501	2009	7
2010	7	30	31	1	2009	7
2010	8	1	24	3	2009	7
2010	8	2	24	2.584962501	2009	7
2010	8	3	14	1	2009	7
2010	8	4	32	1	2009	7
2010	8	6	51	2	2009	7
2010	8	10	58	1	2009	7
2010	8	12	50	1	2009	7

2010	8	15	38	1	2009	7
2011	4	28	62	1	2009	7
2011	4	30	80	1	2009	7
2011	5	1	70	1	2009	7
2011	5	2	55	2.584962501	2009	7
2011	5	3	71	2	2009	7
2011	5	4	76	2	2009	7
2011	5	5	76	2.584962501	2009	7
2011	5	6	45	3.807354922	2009	7
2011	5	7	43	2	2009	8
2011	5	8	63	3.584962501	2009	8
2011	5	9	84	2	2009	8
2011	5	13	36	2	2009	8
2011	5	14	52	3.584962501	2009	8
2011	5	16	57	3.584962501	2009	8
2011	5	17	50	3.321928095	2009	8
2011	5	18	41	1	2009	8
2011	5	21	36	1	2009	8
2011	5	24	11	1	2009	8
2011	5	26	42	1	2009	8
2011	5	28	73	2.584962501	2009	8
2011	5	29	98	1	2009	8
2011	6	1	126	1	2009	8
2011	6	6	70	2	2009	8
2011	6	9	39	2	2009	8
2011	6	10	33	3	2009	8
2011	6	11	23	2.584962501	2009	8
2011	6	12	17	3	2009	8
2011	6	13	15	3.321928095	2009	8
2011	6	14	36	2.584962501	2009	8
2011	6	15	52	2	2009	8
2011	6	18	61	1	2009	8
2011	6	20	39	2.584962501	2009	8
2011	7	2	44	2	2009	8
2011	7	4	46	1	2009	8
2011	7	5	32	1	2009	8
2011	7	7	38	3	2009	8
2011	7	9	49	3.584962501	2009	8
2011	7	10	59	3	2009	8
2011	7	11	75	2.584962501	2009	8
2011	7	13	68	2.584962501	2009	9
2011	7	14	68	3	2009	9
2011	7	15	82	3.807354922	2009	9
2011	7	16	74	3.321928095	2009	9
2011	7	17	93	3.321928095	2009	9
2011	7	18	115	2.584962501	2009	9
2011	7	19	109	2.584962501	2009	9
2011	7	20	71	4	2009	9

2011	7	21	60	3.584962501	2009	9
2011	7	25	29	2.584962501	2009	9
2011	7	29	90	4	2009	9
2011	7	30	91	2	2009	9
2011	8	6	79	2	2009	9
2011	8	8	70	2	2009	9
2011	8	9	61	2	2009	9
2011	8	10	36	3	2009	9
2011	8	11	38	2	2009	9
2011	8	12	29	1	2009	9
2011	8	14	0	2.584962501	2009	9
2011	8	15	12	2	2009	9
2011	8	16	31	1	2009	9
2011	8	18	59	2	2009	9
2011	8	19	60	2	2009	9
2011	8	21	73	1	2009	9
2011	8	22	92	2.584962501	2009	9
2011	8	23	98	1	2009	9
2011	8	24	70	1	2009	9
2011	8	26	86	1	2010	5
2011	8	28	64	1	2010	5
2011	8	30	90	3.584962501	2010	5
2011	8	31	125	4	2010	5
2011	9	2	136	2.584962501	2010	5
2011	9	4	105	3.584962501	2010	5
2011	9	6	89	1	2010	5
2011	9	8	54	1	2010	5
2011	9	9	72	1	2010	5
2011	9	10	80	2	2010	5
2011	9	29	128	1	2010	5
2012	4	21	162	2	2010	5
2012	4	26	117	1	2010	5
2012	4	28	112	1	2010	5
2012	5	9	91	3	2010	5
2012	5	12	101	2.584962501	2010	5
2012	5	13	105	3.584962501	2010	5
2012	5	14	119	2	2010	5
2012	5	16	137	3	2010	5
2012	5	17	110	2.584962501	2010	5
2012	5	18	102	3.321928095	2010	5
2012	5	19	109	3.321928095	2010	6
2012	5	20	116	2.584962501	2010	6
2012	5	21	110	2.584962501	2010	6
2012	5	22	80	1	2010	6
2012	5	23	77	2.584962501	2010	6
2012	5	24	87	1	2010	6
2012	5	26	80	2	2010	6
2012	5	27	82	2.584962501	2010	6

2012	5	30	73	1	2010	6
2012	5	31	89	2.584962501	2010	6
2012	6	2	151	1	2010	6
2012	6	6	153	1	2010	6
2012	6	9	106	2	2010	6
2012	6	10	121	2	2010	6
2012	6	11	128	1	2010	6
2012	6	12	111	2.584962501	2010	6
2012	6	13	113	2	2010	6
2012	6	14	123	2.584962501	2010	6
2012	6	15	117	1	2010	6
2012	6	16	106	2.584962501	2010	6
2012	6	17	88	2.584962501	2010	6
2012	6	19	51	1	2010	6
2012	6	20	30	2	2010	7
2012	6	21	16	3.584962501	2010	7
2012	6	22	16	2.584962501	2010	7
2012	6	23	16	1	2010	7
2012	6	24	20	3	2010	7
2012	6	25	17	2	2010	7
2012	6	26	36	1	2010	7
2012	6	27	70	2.584962501	2010	7
2012	7	1	126	1	2010	7
2012	7	3	125	1	2010	7
2012	7	4	131	1	2010	7
2012	7	5	129	1	2010	7
2012	7	6	132	1	2010	7
2012	7	7	146	2	2010	7
2012	7	8	117	1	2010	7
2012	7	9	122	2.584962501	2010	7
2012	7	10	107	3.321928095	2010	7
2012	7	11	110	2	2010	7
2012	7	12	125	2	2010	7
2012	7	13	129	1	2010	7
2012	7	14	125	3	2010	7
2012	7	15	117	2.584962501	2010	7
2012	7	16	102	3.584962501	2010	7
2012	7	17	86	1	2010	7
2012	7	18	57	2	2010	8
2012	7	19	38	2	2010	8
2012	7	21	29	2.584962501	2010	8
2012	7	22	29	1	2010	8
2012	7	23	63	1	2010	8
2012	7	26	86	1	2010	8
2012	7	28	107	1	2010	8
2012	7	29	111	1	2010	8
2012	8	5	123	3	2010	8
2012	8	6	98	2	2010	8

2012	8	7	122	2.584962501	2010	8
2012	8	8	123	1	2010	8
2012	8	9	158	1	2010	8
2012	8	10	131	1	2010	8
2012	8	14	33	1	2010	8
2012	8	16	32	1	2010	8
2012	8	17	50	3	2010	8
2012	8	18	54	4	2010	8
2012	8	19	72	4.321928095	2010	8
2012	8	20	80	4.321928095	2010	8
2012	8	21	71	3.807354922	2010	8
2012	8	22	69	3.321928095	2010	8
2012	8	23	71	3	2010	8
2012	8	24	71	4.700439718	2010	8
2012	8	25	77	2	2010	8
2012	8	26	78	2	2010	8
2012	8	27	75	3	2010	8
2012	8	28	80	4.459431619	2010	9
2012	8	29	83	2.584962501	2010	9
2012	8	30	126	3	2010	9
2012	8	31	150	3	2010	9
2012	9	1	145	3.807354922	2010	9
2012	9	2	142	2	2010	9
2012	9	3	171	2	2010	9
2012	9	4	159	2	2010	9
2012	9	5	122	2.584962501	2010	9
2012	9	6	116	1	2010	9
2012	9	7	95	3.584962501	2010	9
2012	9	8	82	1	2010	9
2012	9	9	89	3.807354922	2010	9
2012	9	10	70	3.321928095	2010	9
2012	9	11	76	3.584962501	2010	9
2012	9	12	73	3.807354922	2010	9
2012	9	13	58	3.321928095	2010	9
2012	9	16	64	2	2011	5
2012	9	17	55	2	2011	5
2012	9	18	64	1	2011	5
2012	9	20	81	2	2011	5
2012	9	21	84	1	2011	5
2012	9	22	58	1	2011	5
2012	9	23	75	1	2011	5
2012	9	24	95	1	2011	5
2012	9	25	136	1	2011	5
2013	4	8	122	1	2011	5
2013	4	14	110	1	2011	5
2013	4	17	95	1	2011	5
2013	5	2	104	1	2011	5
2013	5	3	98	2	2011	5

2013	5	4	122	2.584962501	2011	6
2013	5	5	110	1	2011	6
2013	5	9	116	1	2011	6
2013	5	13	153	2	2011	6
2013	5	14	160	2.584962501	2011	6
2013	5	15	173	3	2011	6
2013	5	16	206	1	2011	6
2013	5	17	183	2.584962501	2011	6
2013	5	18	133	2.584962501	2011	6
2013	5	19	127	2	2011	6
2013	5	23	108	2.584962501	2011	7
2013	6	7	48	1	2011	7
2013	6	9	35	3.321928095	2011	7
2013	6	10	26	1	2011	7
2013	6	15	95	1	2011	7
2013	6	16	104	2	2011	7
2013	6	18	113	2	2011	7
2013	6	21	139	2.584962501	2011	7
2013	6	22	137	2	2011	7
2013	6	23	118	1	2011	7
2013	6	24	108	2.584962501	2011	7
2013	6	25	77	1	2011	7
2013	6	26	60	3	2011	7
2013	6	27	61	2.584962501	2011	7
2013	7	2	112	3	2011	7
2013	7	3	109	2	2011	7
2013	7	4	121	1	2011	7
2013	7	5	109	2.584962501	2011	7
2013	7	6	124	1	2011	7
2013	7	7	141	2	2011	7
2013	7	8	122	1	2011	7
2013	7	10	80	1	2011	8
2013	7	11	79	1	2011	8
2013	7	12	73	2.584962501	2011	8
2013	7	13	36	2	2011	8
2013	7	14	60	3.807354922	2011	8
2013	7	15	77	5.247927513	2011	8
2013	7	16	77	1	2011	8
2013	7	22	57	3	2011	8
2013	7	24	70	1	2011	8
2013	7	25	85	2	2011	8
2013	7	27	68	2.584962501	2011	8
2013	7	28	73	1	2011	8
2013	7	29	103	3	2011	8
2013	7	30	89	2.584962501	2011	8
2013	7	31	103	1	2011	8
2013	8	1	96	4.321928095	2011	8
2013	8	2	114	2.584962501	2011	8

2013	8	3	107	3	2011	8
2013	8	4	86	4	2011	8
2013	8	5	82	4.169925001	2011	8
2013	8	6	71	4.169925001	2011	8
2013	8	7	86	3.584962501	2011	8
2013	8	8	89	3.807354922	2011	8
2013	8	9	64	3.584962501	2011	8
2013	8	10	72	4.321928095	2011	8
2013	8	11	81	4.906890596	2011	8
2013	8	12	90	4.807354922	2011	8
2013	8	13	108	5.247927513	2011	8
2013	8	14	108	4.700439718	2011	8
2013	8	15	124	3.584962501	2011	8
2013	8	16	96	4.169925001	2011	9
2013	8	17	100	4.321928095	2011	9
2013	8	18	110	2	2011	9
2013	8	19	134	2	2011	9
2013	8	20	129	2	2011	9
2013	8	21	146	2.584962501	2011	9
2013	8	22	139	2.584962501	2011	9
2013	8	23	138	3	2011	9
2013	8	24	78	3.321928095	2011	9
2013	8	25	50	3.321928095	2011	9
2013	8	26	43	1	2011	9
2013	8	27	56	2.584962501	2011	9
2013	8	28	50	3.807354922	2011	9
2013	8	29	53	2	2011	9
2013	8	30	71	2	2011	9
2013	8	31	74	1	2012	5
2013	9	1	74	2.584962501	2012	5
2013	9	2	90	3	2012	5
2013	9	3	72	2	2012	5
2013	9	4	78	1	2012	5
2013	9	5	65	2.584962501	2012	5
2013	9	6	49	2	2012	5
2013	9	7	37	1	2012	5
2013	9	8	25	2.584962501	2012	5
2013	9	9	13	2	2012	5
2013	9	10	18	3	2012	5
2013	9	11	60	1	2012	5
2013	9	14	25	2.584962501	2012	5
2013	9	15	13	2	2012	5
2013	9	16	25	2	2012	5
2013	9	17	52	1	2012	5
2013	9	18	63	2	2012	5
2013	9	20	90	2	2012	5
2013	9	21	77	1	2012	5
2013	9	22	79	1	2012	5

2013	10	1	48	2.584962501	2012	5
2013	10	2	60	1	2012	5
2014	4	19	178	1	2012	5
2014	4	21	150	1	2012	5
2014	4	22	123	1	2012	5
2014	5	1	88	1	2012	5
2014	5	13	133	2	2012	5
2014	5	15	156	1	2012	6
2014	5	19	102	1	2012	6
2014	5	21	85	3.807354922	2012	6
2014	5	23	91	1	2012	6
2014	5	25	109	1	2012	6
2014	5	28	70	3.321928095	2012	6
2014	5	29	48	1	2012	6
2014	5	31	57	1	2012	6
2014	6	1	64	4	2012	6
2014	6	2	62	1	2012	6
2014	6	3	74	2.584962501	2012	6
2014	6	4	64	2	2012	6
2014	6	5	87	2.584962501	2012	6
2014	6	14	137	1	2012	6
2014	6	16	84	1	2012	6
2014	6	19	95	1	2012	6
2014	6	20	80	1	2012	6
2014	6	26	67	2	2012	6
2014	6	27	67	1	2012	6
2014	6	28	71	1	2012	6
2014	6	30	132	1	2012	6
2014	7	2	145	3.321928095	2012	6
2014	7	4	172	2	2012	6
2014	7	5	194	1	2012	6
2014	7	6	185	1	2012	6
2014	7	12	119	1	2012	6
2014	7	14	62	1	2012	6
2014	7	17	0	2	2012	6
2014	7	18	15	1	2012	7
2014	7	20	35	2.584962501	2012	7
2014	7	21	17	1	2012	7
2014	7	22	39	1	2012	7
2014	7	23	65	2	2012	7
2014	7	24	62	1	2012	7
2014	7	26	58	3	2012	7
2014	7	27	78	1	2012	7
2014	7	28	109	3	2012	7
2014	7	29	137	2.584962501	2012	7
2014	7	30	122	1	2012	7
2014	8	1	165	2	2012	7
2014	8	2	165	2.584962501	2012	7

2014	8	3	153	2	2012	7
2014	8	4	146	1	2012	7
2014	8	5	128	1	2012	7
2014	8	6	113	1	2012	7
2014	8	8	89	1	2012	7
2014	8	11	69	1	2012	7
2014	8	12	75	1	2012	7
2014	8	13	79	1	2012	7
2014	8	16	109	2.584962501	2012	7
2014	8	17	116	2.584962501	2012	7
2014	8	19	93	1	2012	7
2014	8	20	102	2	2012	7
2014	8	21	120	2.584962501	2012	7
2014	8	22	125	2	2012	7
2014	8	23	150	2	2012	8
2014	8	24	149	2.584962501	2012	8
2014	8	25	116	3	2012	8
2014	8	26	86	3	2012	8
2014	8	27	95	4.906890596	2012	8
2014	8	28	82	4.584962501	2012	8
2014	8	29	66	4	2012	8
2014	8	30	76	3	2012	8
2014	8	31	82	3.321928095	2012	8
2014	9	1	96	3.807354922	2012	8
2014	9	3	102	2.584962501	2012	8
2014	9	4	120	3.584962501	2012	8
2014	9	5	116	3	2012	8
2014	9	6	142	1	2012	8
2014	9	7	165	3.321928095	2012	8
2014	9	8	154	1	2012	8
2014	9	11	160	2.584962501	2012	8
2014	9	12	126	1	2012	8
2014	9	13	125	2	2012	8
2014	9	14	104	1	2012	8
2014	9	18	82	1	2012	8
2014	9	19	79	1	2012	8
2014	9	20	80	2	2012	8
2014	9	21	95	2	2012	8
2014	9	24	108	1	2012	8
2014	9	25	144	2	2012	8
2014	9	27	181	1	2012	8
2014	9	28	193	1	2012	8
2014	9	29	180	1	2012	8
2014	10	1	135	2.584962501	2012	8
2014	10	2	126	1	2012	8
2015	5	1	19	1	2012	9
2015	5	4	103	1	2012	9
2015	5	12	165	1	2012	9

2015	5	13	172	1	2012	9
2015	5	15	124	1	2012	9
2015	5	19	77	1	2012	9
2015	5	20	59	2	2012	9
2015	5	22	73	2	2012	9
2015	5	23	72	1	2012	9
2015	5	24	78	1	2012	9
2015	5	27	14	1	2012	9
2015	5	28	12	2	2012	9
2015	5	29	33	1	2012	9
2015	5	30	31	1	2012	9
2015	5	31	29	1	2012	9
2015	6	3	49	2	2012	9
2015	6	4	80	2	2012	9
2015	6	5	90	3	2012	9
2015	6	7	124	2.584962501	2012	9
2015	6	10	93	1	2012	9
2015	6	12	98	3	2012	9
2015	6	13	97	2.584962501	2012	9
2015	6	14	90	1	2012	9
2015	6	15	54	2	2012	9
2015	6	16	70	1	2012	9
2015	6	19	68	1	2012	9
2015	6	20	64	1	2012	9
2015	6	22	56	1	2012	9
2015	6	23	45	3	2012	9
2015	6	24	36	2.584962501	2013	5
2015	6	26	23	2	2013	5
2015	6	27	21	1	2013	5
2015	6	28	32	1	2013	5
2015	6	29	39	2	2013	5
2015	6	30	41	1	2013	5
2015	7	5	109	1	2013	5
2015	7	6	97	1	2013	5
2015	7	12	71	1	2013	5
2015	7	14	43	1	2013	5
2015	7	17	50	2	2013	5
2015	7	19	44	2	2013	5
2015	7	20	36	2.584962501	2013	5
2015	7	21	36	1	2013	5
2015	7	22	36	1	2013	5
2015	7	24	31	1	2013	5
2015	7	26	40	2.584962501	2013	5
2015	7	27	51	1	2013	5
2015	7	28	62	1	2013	6
2015	7	29	67	2	2013	6
2015	7	30	76	1	2013	6
2015	8	3	61	2	2013	6

2015	8	4	90	1
2015	8	6	108	2
2015	8	8	93	2
2015	8	11	78	1
2015	8	12	66	2
2015	8	13	55	2.584962501
2015	8	16	38	2
2015	8	17	33	2
2015	8	19	50	2.584962501
2015	8	20	65	2.584962501
2015	8	21	73	2
2015	8	22	71	2.584962501
2015	8	23	81	1
2015	8	24	81	2
2015	8	25	69	3
2015	8	26	47	3
2015	8	27	55	4.584962501
2015	8	28	44	4.169925001
2015	8	29	56	3.584962501
2015	8	30	56	2.584962501
2015	8	31	35	2
2015	9	8	42	3.584962501
2015	9	9	57	2.584962501
2015	9	12	89	3
2015	9	14	56	4
2015	9	15	68	3
2015	9	16	86	1
2015	9	24	101	4
2015	9	25	152	3.584962501

2013	6
2013	6
2013	6
2013	6
2013	6
2013	6
2013	6
2013	6
2013	6
2013	6
2013	6
2013	6
2013	6
2013	6
2013	6
2013	6
2013	6
2013	6
2013	7
2013	7
2013	7
2013	7
2013	7
2013	7
2013	7
2013	7
2013	7
2013	7
2013	7
2013	7
2013	7
2013	7
2013	7
2013	7
2013	7
2013	7
2013	7
2013	7
2013	7
2013	7
2013	8
2013	8
2013	8
2013	8
2013	8
2013	8
2013	8
2013	8

2013	8
2013	8
2013	8
2013	8
2013	8
2013	8
2013	8
2013	8
2013	8
2013	8
2013	8
2013	8
2013	8
2013	8
2013	8
2013	8
2013	8
2013	8
2013	8
2013	9
2013	9
2013	9
2013	9
2013	9
2013	9
2013	9
2013	9
2013	9
2013	9
2013	9
2013	9
2013	9
2013	9
2013	9
2013	9
2014	5
2014	5
2014	5
2014	5
2014	5
2014	5
2014	5
2014	5
2014	5
2014	5
2014	5
2014	5
2014	5

2014	5
2014	5
2014	6
2014	6
2014	6
2014	6
2014	6
2014	6
2014	6
2014	6
2014	6
2014	6
2014	6
2014	6
2014	6
2014	6
2014	6
2014	6
2014	6
2014	6
2014	6
2014	6
2014	6
2014	6
2014	6
2014	6
2014	6
2014	6
2014	6
2014	6
2014	7
2014	7
2014	7
2014	7
2014	7
2014	7
2014	7
2014	7
2014	7
2014	7
2014	7
2014	7
2014	7
2014	7
2014	7
2014	7
2014	7
2014	7

2014	7
2014	7
2014	7
2014	7
2014	7
2014	7
2014	7
2014	7
2014	7
2014	7
2014	7
2014	7
2014	8
2014	8
2014	8
2014	8
2014	8
2014	8
2014	8
2014	8
2014	8
2014	8
2014	8
2014	8
2014	8
2014	8
2014	8
2014	8
2014	8
2014	8
2014	8
2014	8
2014	8
2014	8
2014	8
2014	8
2014	8
2014	8
2014	8
2014	8
2014	8
2014	8
2014	8
2014	9
2014	9
2014	9
2014	9
2014	9

2014	9
2014	9
2014	9
2014	9
2014	9
2014	9
2014	9
2015	5
2015	5
2015	5
2015	5
2015	5
2015	5
2015	5
2015	5
2015	5
2015	5
2015	5
2015	5
2015	5
2015	5
2015	5
2015	5
2015	5
2015	5
2015	5
2015	5
2015	5
2015	6
2015	6
2015	6
2015	6
2015	6
2015	6
2015	6
2015	6
2015	6
2015	6
2015	6
2015	6
2015	6
2015	6
2015	6
2015	6
2015	6
2015	6
2015	6
2015	7

2015	7
2015	7
2015	7
2015	7
2015	7
2015	7
2015	7
2015	7
2015	7
2015	7
2015	7
2015	7
2015	7
2015	7
2015	7
2015	7
2015	7
2015	7
2015	7
2015	7
2015	7
2015	7
2015	7
2015	7
2015	7
2015	7
2015	7
2015	7
2015	7
2015	8
2015	8
2015	8
2015	8
2015	8
2015	8
2015	8
2015	8
2015	8
2015	8
2015	8
2015	8
2015	8
2015	8
2015	8
2015	8
2015	8
2015	8
2015	8

2015	8
2015	8
2015	8
2015	8
2015	8
2015	8
2015	8
2015	8
2015	8
2015	8
2015	8
2015	8
2015	9
2015	9
2015	9
2015	9
2015	9
2015	9
2015	9
2015	9
2015	9
2015	9
2015	9
2015	9
2015	9
2015	9
2015	9
2015	9
2015	9
2015	9
2015	9
2015	9
2015	9
2015	9
2015	9
2015	9
2015	9
2016	5
2016	5
2016	5
2016	5
2016	5
2016	5
2016	5
2016	5
2016	5
2016	5
2016	5

2016	5
2016	5
2016	5
2016	5
2016	5
2016	5
2016	5
2016	5
2016	5
2016	5
2016	5
2016	6
2016	6
2016	6
2016	6
2016	6
2016	6
2016	6
2016	6
2016	6
2016	6
2016	6
2016	6
2016	6
2016	6
2016	6
2016	6
2016	6
2016	6
2016	6
2016	6
2016	6
2016	6
2016	6
2016	6
2016	6
2016	6
2016	6
2016	6
2016	6
2016	6
2016	7
2016	7
2016	7
2016	7
2016	7
2016	7
2016	7

2016	7
2016	7
2016	7
2016	7
2016	7
2016	7
2016	7
2016	7
2016	7
2016	7
2016	7
2016	7
2016	7
2016	7
2016	7
2016	7
2016	7
2016	7
2016	7
2016	7
2016	7
2016	7
2016	7
2016	7
2016	8
2016	8
2016	8
2016	8
2016	8
2016	8
2016	8
2016	8
2016	8
2016	8
2016	8
2016	8
2016	8
2016	8
2016	8
2016	8
2016	8
2016	8
2016	8
2016	8
2016	8
2016	8
2016	8
2016	8

2016	8
2016	8
2016	8
2016	8
2016	8
2016	8
2016	8
2016	9
2016	9
2016	9
2016	9
2016	9
2016	9
2016	9
2016	9
2016	9
2016	9
2016	9
2016	9
2016	9
2016	9
2016	9
2016	9
2016	9
2016	9
2016	9
2016	9
2016	9
2017	5
2017	5
2017	5
2017	5
2017	5
2017	5
2017	5
2017	5
2017	5
2017	5
2017	5
2017	5
2017	5
2017	5
2017	5
2017	5
2017	6
2017	6
2017	6
2017	6

2017	6
2017	6
2017	6
2017	6
2017	6
2017	6
2017	6
2017	6
2017	6
2017	6
2017	6
2017	6
2017	6
2017	6
2017	6
2017	6
2017	6
2017	6
2017	6
2017	6
2017	6
2017	6
2017	6
2017	6
2017	6
2017	6
2017	7
2017	7
2017	7
2017	7
2017	7
2017	7
2017	7
2017	7
2017	7
2017	7
2017	7
2017	7
2017	7
2017	7
2017	7
2017	7
2017	7
2017	7
2017	7
2017	7
2017	7
2017	7

2017	7
2017	7
2017	7
2017	7
2017	7
2017	7
2017	7
2017	8
2017	8
2017	8
2017	8
2017	8
2017	8
2017	8
2017	8
2017	8
2017	8
2017	8
2017	8
2017	8
2017	8
2017	8
2017	8
2017	8
2017	8
2017	8
2017	8
2017	8
2017	8
2017	8
2017	8
2017	8
2017	8
2017	8
2017	8
2017	9
2017	9
2017	9
2017	9
2017	9
2017	9
2017	9
2017	9
2017	9
2018	5
2018	5
2018	5
2018	5

2018	5
2018	5
2018	5
2018	5
2018	6
2018	6
2018	6
2018	6
2018	6
2018	6
2018	6
2018	7
2018	7
2018	7
2018	7
2018	7
2018	7
2018	7
2018	7
2018	7
2018	7
2018	7
2018	7
2018	7
2018	7
2018	8
2018	8
2018	8
2018	8
2018	8
2018	8
2018	8
2018	8
2018	8
2018	8
2018	8
2018	8
2018	8
2018	8
2018	8
2018	8
2018	8
2018	8
2018	8
2018	8
2018	8
2018	8
2018	8

2018	8
2018	8
2018	8
2018	8
2018	8
2018	9
2018	9
2018	9
2018	9

Date	Sunspot	log2(moths No.)+1
2	13	1
4	20	2
19	30	2
20	32	2
21	20	2
24	0	3
25	0	3.321928095
26	10	2.584962501
27	11	2
30	0	1
25	0	1
26	0	2.584962501
31	0	3
1	11	3
3	11	3.321928095
5	16	1
7	28	4.700439718
11	13	4
12	13	4.459431619
13	0	4.169925001
16	0	3
20	11	4.459431619
23	24	3
1	12	2
3	13	1
5	12	3.584962501
6	0	1
8	37	3.584962501
11	29	2
15	0	1
17	0	3.321928095
19	0	2.584962501
1	26	3.321928095
2	31	4
3	31	5.321928095
4	29	4.584962501
6	14	4
8	15	3.321928095
9	19	4.321928095
10	20	4.700439718
11	29	4.169925001
12	23	4.906890596
13	16	5.087462841
14	29	5.087462841
15	26	3.807354922
16	14	4
17	15	3.807354922

18	12	3
19	15	3.321928095
4	11	1
5	12	3.321928095
7	11	1
8	14	3
9	12	3.321928095
10	23	2
17	23	1
18	29	2
19	37	2
20	54	1
24	41	1
26	48	2
29	19	1
30	17	1
31	26	1
3	24	1
4	18	1
12	26	1
13	29	1
14	31	1
16	33	1
18	29	1
23	15	2.584962501
24	15	2.584962501
25	31	1
27	20	1
28	13	3.321928095
29	13	4
30	0	4.321928095
1	0	2.584962501
2	0	2
3	10	3
4	11	2
5	10	3.321928095
6	12	1
8	20	4.169925001
9	21	4.459431619
11	0	2
12	0	2
13	0	2
14	11	4.169925001
15	0	1
16	0	1
17	9	2
18	9	1
19	0	2

22	14	1
23	25	2
25	48	2.584962501
28	14	1
29	14	2
30	0	1
1	0	4.807354922
2	12	4.321928095
3	19	5.459431619
4	13	3.321928095
5	25	4
6	53	4
7	60	4.459431619
8	66	3.584962501
9	56	4.321928095
10	31	3.807354922
11	53	3.584962501
12	70	4.321928095
13	67	3.321928095
14	57	3.584962501
15	54	3.584962501
16	31	4
17	26	4
18	23	3.321928095
19	13	4
20	12	3.807354922
21	13	2.584962501
22	12	3
23	13	3
24	0	2
25	22	3.584962501
26	31	2
27	35	3.807354922
28	48	3
29	51	1
31	78	3
1	76	1
3	56	2.584962501
4	49	1
5	58	1
7	79	2
15	76	1
25	54	2.584962501
26	57	1
27	67	1
28	43	1
29	37	1
30	53	1

1	63	1
4	95	2
6	92	1
7	85	1
8	85	2
12	106	1
13	117	2
16	63	4.906890596
17	86	3.321928095
18	70	4.584962501
19	67	4
20	77	2.584962501
3	138	1
5	137	1
7	74	2
9	46	1
10	71	3
12	80	2
13	64	2.584962501
14	60	3.807354922
15	80	3
16	97	3.321928095
17	86	2
18	61	2
19	51	2.584962501
21	113	3
22	132	4
26	91	1
27	94	3
28	123	2
29	107	2.584962501
30	83	2.584962501
31	99	2
1	84	1
4	87	3.321928095
5	92	1
6	129	2.584962501
7	137	3.321928095
8	127	3.584962501
9	164	3.321928095
10	156	4.906890596
11	155	5
12	159	3.584962501
13	121	3.807354922
14	123	2
15	96	2.584962501
16	123	4.169925001
17	114	2.584962501

18	109	3.807354922
19	133	4.584962501
20	120	5.700439718
21	117	2.584962501
22	106	2.584962501
23	110	2
24	104	5.95419631
25	96	4.584962501
26	114	5.087462841
27	131	4.459431619
28	126	5.247927513
29	134	4.906890596
30	154	6.169925001
31	143	5.087462841
1	142	4.807354922
2	121	5.321928095
3	112	5.392317423
4	97	5.807354922
5	114	4.700439718
6	159	5.64385619
7	165	5.247927513
8	178	5.64385619
9	169	2
10	159	5.247927513
13	135	3.807354922
14	111	2
15	85	2
19	132	1
23	192	2
10	191	2
15	148	1
16	148	2
20	135	2.584962501
22	138	2
23	121	1
25	129	2
26	160	2
27	167	2
28	167	2
29	162	3
30	150	3
6	220	1
7	185	1
28	254	4.700439718
6	186	2.584962501
7	179	1
11	176	1
12	199	2.584962501

13	157	1
14	128	1
15	122	1
16	118	1
17	121	1
19	121	2.584962501
20	118	1
21	121	1
22	144	3.584962501
23	148	3.584962501
24	173	3
27	176	1
28	220	2
2	250	1
3	229	1
5	193	3
9	115	1
10	82	1
11	79	3.807354922
12	91	2
13	102	1
14	87	2
15	74	1
16	67	2.584962501
17	55	3.321928095
18	58	1
21	88	1
22	103	3
23	115	3
24	131	3.807354922
25	196	3
26	206	3
27	194	3
28	223	3.584962501
29	231	3.584962501
30	228	4
31	165	1
1	140	4
2	122	4
3	115	1
4	103	2
5	106	4
6	86	1
7	103	2
8	113	1
12	126	2.584962501
19	228	1
21	222	1

22	195	1
25	157	1
26	160	2.584962501
27	145	3.584962501
28	169	2
29	160	1
30	127	3.807354922
31	91	5.584962501
1	128	1
3	113	2
4	152	1
19	218	1
22	191	3
24	179	1
25	167	1
26	194	1
27	208	1
28	173	2.584962501
29	164	1
1	208	2
2	202	2
3	178	2
4	164	1
6	221	1
7	254	1
8	254	2
9	257	1
10	309	4
12	267	4.584962501
13	279	3
14	236	3.584962501
15	213	3.584962501
19	353	3
20	346	3.584962501
21	332	4.459431619
22	310	2
23	286	3.584962501
24	246	4
25	254	2
26	191	3.807354922
27	181	3
28	172	3
29	162	1
30	161	4.321928095
31	134	5.169925001
1	147	3.584962501
2	152	3.321928095
3	148	3

4	152	2.584962501
5	199	3.584962501
6	198	3.584962501
7	227	4.321928095
8	194	4.459431619
10	213	5.087462841
11	228	2
12	235	1
13	243	2
14	282	1
15	253	1
16	246	3.584962501
18	194	1
19	184	1
20	147	1
21	106	2
22	93	2
23	93	2.584962501
24	106	3
25	112	1
26	109	1
27	156	1
4	191	1
31	101	1
1	88	1
3	150	2.584962501
5	161	2.584962501
6	180	2
7	195	3
8	215	3.584962501
9	254	3.321928095
12	259	2.584962501
14	273	4.906890596
15	282	2
16	289	1
17	270	2.584962501
19	214	3
22	229	3.584962501
23	235	2
24	220	1
25	198	2.584962501
26	173	2
27	162	1
28	135	2
30	98	2.584962501
2	125	2.584962501
3	120	1
5	93	3.584962501

7	71	2.584962501
9	107	2
12	135	2
15	153	2.584962501
17	185	3.807354922
18	191	2
19	183	2.584962501
22	150	3.584962501
23	152	1
27	90	3
29	69	3.321928095
31	78	2
2	123	1
3	141	2.584962501
6	182	2.584962501
7	179	2.584962501
8	178	2.584962501
10	150	3.807354922
11	170	4
12	170	4.321928095
13	138	4.584962501
14	141	4.459431619
15	161	3
16	193	4.584962501
17	178	3.321928095
18	161	2.584962501
19	152	2
20	153	2.584962501
21	167	3
24	176	3
26	153	1
28	184	3.321928095
31	175	2
1	163	2.584962501
20	289	1
5	184	1
6	197	1
7	197	1
9	165	1
10	128	3
11	99	2.584962501
12	109	2
13	80	1
14	107	1
16	117	1
17	88	3.321928095
18	127	3.321928095
19	115	2

20	108	2
21	83	1
22	83	1
26	108	1
27	96	1
28	88	1
1	94	1
3	129	2.584962501
4	133	2.584962501
5	142	2
21	124	1
22	147	2
23	196	2
25	215	2.584962501
26	265	1
27	294	2
28	310	2
30	281	1
31	239	3
1	207	3
2	199	3
3	183	2
4	143	2
5	127	2
7	133	2
8	115	4
9	110	3.321928095
10	110	2
11	149	2
13	202	2.584962501
14	267	2
15	279	2
29	124	2
30	146	1
31	160	2
1	206	3
2	233	3.321928095
3	252	1
5	226	1
6	202	3.321928095
7	206	1
8	213	2
9	199	1
10	202	1
11	187	2
12	187	2
14	149	1
17	199	2

18	207	2.584962501
19	192	2
20	195	1
24	177	1
11	57	1
12	60	1
20	97	1
23	91	1
28	99	1
2	58	1
3	61	1
5	91	1
6	132	2
7	150	1
9	170	2
10	170	1
12	176	1
13	147	2
14	124	1
15	97	1
16	87	2
18	104	1
19	117	1
21	95	1
23	101	1
28	143	1
29	144	2
4	103	1
9	139	1
11	94	1
14	100	1
17	117	1
18	106	1
20	98	2
23	121	1
26	141	2
27	143	3.584962501
28	151	3.321928095
29	135	2
30	117	3.321928095
31	103	3
1	74	2
3	76	1
9	27	3.321928095
11	55	2.584962501
12	47	3.807354922
13	48	4.169925001
16	74	2.584962501

17	94	1
21	91	1
30	61	2
10	48	2
14	54	1
18	103	1
19	106	1
21	134	1
23	94	1
25	57	1
26	52	1
27	48	1
29	40	1
30	34	1
4	31	1
15	135	1
25	94	1
30	39	2
31	38	5.392317423
3	51	2.584962501
4	56	2.584962501
5	36	3.584962501
6	56	3.807354922
7	75	3
8	67	3.584962501
9	85	3.807354922
10	99	4.321928095
11	107	5.523561956
12	116	5.392317423
13	130	3.584962501
14	116	3.807354922
15	104	3.584962501
16	92	4.169925001
17	75	5
18	70	5.321928095
19	61	6.044394119
20	85	6.882643049
21	97	3.321928095
22	113	3.321928095
23	96	4.169925001
24	65	4.321928095
25	53	4.169925001
26	41	6.392317423
27	36	3
28	34	1
29	27	1
30	17	2.584962501
31	15	5.169925001

1	14	6.285402219
2	14	4.584962501
3	32	4.807354922
4	35	4.584962501
5	65	4.321928095
6	56	4.584962501
7	67	5
8	83	7.044394119
9	90	3.321928095
10	77	2.584962501
11	74	3
12	76	2.584962501
13	69	3
14	56	4.584962501
15	69	2
16	67	3.807354922
17	63	3
18	58	4.459431619
19	60	4.321928095
20	47	3.584962501
21	42	3
22	30	4
23	18	2.584962501
24	18	2
25	18	2
26	26	3
27	26	2.584962501
26	64	1
30	67	2
31	84	3
1	76	3.321928095
2	58	3.584962501
3	48	4.584962501
4	71	3.807354922
5	89	4.459431619
6	92	4
7	105	2.584962501
8	98	3
9	97	2.584962501
10	101	1
11	79	2
12	75	2.584962501
13	45	1
14	48	3
15	58	1
18	46	1
19	42	1
2	148	2.584962501

3	157	2.584962501
4	167	1
5	160	2
6	131	3
7	117	3.321928095
8	100	3.584962501
9	89	3.807354922
10	65	3.584962501
11	64	3.321928095
12	59	4.459431619
13	64	4.459431619
14	47	4.459431619
15	33	4
16	17	4.169925001
17	12	3
18	0	3.321928095
19	14	2.584962501
20	0	2
21	0	2
22	12	1
23	25	1
24	17	2
27	17	1
2	115	2.584962501
3	80	2.584962501
4	66	2.584962501
5	66	3.807354922
6	55	3.321928095
7	65	4.169925001
8	61	4.169925001
9	57	4.321928095
10	27	3.321928095
11	37	5.64385619
12	45	5.906890596
13	37	6.285402219
14	43	6.357552005
15	45	5.754887502
16	40	6.491853096
17	33	6.087462841
18	32	6.247927513
19	73	5.64385619
20	80	5.321928095
21	65	5.321928095
22	63	4.807354922
23	60	3.321928095
24	70	4
25	68	3
26	61	1

27	70	2.584962501
28	76	1
29	71	1
1	29	1
2	24	2
6	14	1
7	24	1
9	48	2.584962501
11	58	1
15	66	2.584962501
16	56	2
19	13	1
20	32	2
24	43	1
25	42	2.584962501
26	47	3.321928095
27	53	3
28	62	3.321928095
29	58	2.584962501
30	53	3.321928095
31	45	2
1	14	3.807354922
2	0	4
3	0	3.807354922
4	19	4
5	26	4
6	32	4.459431619
7	58	4.321928095
8	58	3.321928095
9	47	3
10	49	1
11	37	2
12	30	2.584962501
13	28	1
14	14	2
15	14	1
30	44	1
2	31	2
4	36	1
6	36	4.321928095
7	36	2.584962501
8	35	3.584962501
9	31	3.807354922
10	15	3
11	15	2.584962501
12	13	3.321928095
13	0	3.807354922
14	15	3.321928095

15	16	2.584962501
16	20	2
17	22	1
24	20	1
25	18	2
26	18	2
30	31	3
31	27	3.321928095
1	11	4.459431619
2	11	2
3	15	4.459431619
4	0	4
5	0	5.169925001
6	0	3.584962501
7	0	3.584962501
8	11	4.321928095
9	26	2.584962501
10	27	3.321928095
11	26	5.087462841
12	24	2.584962501
13	26	4
14	31	3.321928095
15	31	4
16	31	3
17	27	5.087462841
18	31	3.321928095
19	24	3
20	16	3.321928095
21	26	3.584962501
22	15	4.459431619
23	19	2.584962501
24	19	3
25	24	3.584962501
26	23	3.807354922
27	34	4.807354922
29	27	3.321928095
30	19	1
31	35	3
1	35	2.584962501
2	16	2
3	0	1
16	13	2.584962501
17	25	1
3	20	1
4	18	3.321928095
7	13	1
8	15	3.584962501
9	18	3

10	20	2
11	23	1
12	22	1
15	30	3
16	32	2
17	40	2
18	42	1
20	23	2
24	15	1
26	0	1
27	0	1
28	0	1
29	12	1
3	55	2
8	60	1
11	16	1
13	14	1
16	0	1
17	0	1
18	0	1
19	0	2.584962501
20	0	3.321928095
22	0	3
23	0	3
24	0	2.584962501
25	12	3.321928095
27	14	1
28	16	2.584962501
30	32	1
1	30	1
2	17	1
5	14	1
8	16	1
10	27	2.584962501
11	27	3
12	23	1
13	41	1
14	42	2
16	31	1
18	14	2
20	0	1
22	0	3
23	0	2
24	0	2.584962501
25	0	2.584962501
27	0	3.584962501
28	14	3.321928095
29	14	4

30	14	4.169925001
31	0	4.459431619
1	0	4
2	0	4.807354922
3	11	4.700439718
4	11	4.906890596
5	11	5.087462841
6	13	6.044394119
7	13	6.491853096
8	15	6.64385619
9	15	7.977279923
10	15	6.781359714
11	13	7.906890596
12	13	7.727920455
13	13	4.906890596
14	0	5.906890596
15	0	5.64385619
16	0	7.554588852
17	0	5.754887502
18	0	5.169925001
19	0	5.087462841
20	0	4
21	15	5.087462841
22	13	3
23	13	3
24	13	2
25	13	1
26	13	3
27	13	2.584962501
30	13	1
31	28	1
1	24	1
2	13	2
3	13	3.584962501
4	13	3.584962501
5	13	2
6	0	2
7	0	3
8	0	2
9	0	3.321928095
10	0	3.321928095
11	0	3
12	0	2.584962501
13	0	2
14	0	1
15	0	2
16	0	1
2	0	1

8	12	2
12	0	1
15	0	1
9	0	3
10	0	4.321928095
11	0	4.459431619
12	0	4
13	12	5.247927513
14	0	4.169925001
15	13	5.95419631
16	20	5.392317423
17	17	5.392317423
18	22	2.584962501
19	16	4.169925001
20	10	3.807354922
21	0	5.459431619
22	0	5.087462841
23	0	5.087462841
24	0	5.392317423
25	0	4
26	0	5
31	0	6.321928095
1	0	6.357552005
2	0	6.857980995
3	0	7.266786541
4	0	5.906890596
5	14	3.321928095
6	0	3
7	0	5.247927513
8	0	6.285402219
9	0	3
10	14	3
11	12	3
12	11	3.807354922
13	12	3.584962501
14	0	1
15	11	3
16	11	3
17	11	2.584962501
18	12	4.807354922
19	11	4
20	12	3.584962501
21	12	3.807354922
22	12	4.459431619
23	0	5
24	0	4.700439718
25	0	4.584962501
26	0	4.584962501

27	0	4.807354922
28	0	5.169925001
29	0	6.209453366
30	0	5.584962501
1	0	5.247927513
2	0	5.169925001
3	0	6.209453366
4	0	5.169925001
5	0	7.087462841
6	0	6.832890014
7	0	6.459431619
8	0	5.087462841
9	0	5.392317423
10	0	5.459431619
11	0	6
12	0	5
13	0	5.807354922
14	0	5.807354922
15	0	4.169925001
16	0	5.459431619
17	0	1
18	6	3.807354922
20	6	2
21	0	5.247927513
22	0	6.614709844
23	0	7.64385619
24	0	4.584962501
26	0	5
27	0	5.247927513
28	0	9.442943496
29	0	8.787902559
30	0	9.442943496
31	0	7.882643049
1	0	8.383704292
2	0	8.339850003
3	0	8.199672345
4	0	7.906890596
5	0	8.303780748
6	0	7.768184325
7	0	8.426264755
8	0	8.614709844
9	0	8.375039431
10	0	8.62935662
11	0	8.294620749
12	0	7.741466986
13	0	7.375039431
14	0	5.754887502
15	0	3.807354922

16	0	5
17	0	4.584962501
18	0	4.169925001
19	0	2.584962501
20	0	3
21	5	3.807354922
22	5	5.584962501
23	0	4
24	0	4.584962501
25	0	4.807354922
26	0	3.321928095
27	0	4
28	0	2.584962501
29	0	4.321928095
30	0	3.584962501
31	0	3
1	0	2.584962501
2	0	3.321928095
3	0	3
4	0	3
5	0	3.321928095
6	0	4.169925001
7	0	3.584962501
8	0	3.321928095
9	0	3.584962501
10	0	2
16	0	2
17	0	1
18	0	1
19	0	1
20	0	2
21	0	1
22	9	2
23	10	2
12	0	3.807354922
13	7	4.459431619
14	10	4.584962501
15	9	4
16	8	4.584962501
17	9	5
18	8	4.906890596
19	7	5.459431619
20	0	5.321928095
21	0	5
22	8	4.321928095
23	8	5
24	0	5.321928095
27	0	4.321928095

28	0	2
29	0	4.584962501
30	0	3.807354922
31	10	2.584962501
1	24	3.321928095
2	26	2.584962501
3	24	3
4	19	5.087462841
6	0	2
10	0	2
12	0	1
13	0	1
15	0	2.584962501
16	0	2
17	15	2
18	0	2
19	0	2.584962501
20	0	3
21	15	3.321928095
22	17	2
23	15	2.584962501
24	17	3.584962501
25	0	4.906890596
26	0	2
29	0	3.807354922
30	0	3.807354922
1	0	4
2	0	2
3	0	3.321928095
4	24	3.807354922
5	27	1
6	24	3
7	19	1
8	21	2
9	17	2.584962501
10	15	3.584962501
11	0	3.321928095
12	0	3.807354922
13	0	5.169925001
14	0	4.906890596
15	0	2.584962501
16	0	5.321928095
17	0	4
18	0	5.523561956
19	0	4.906890596
20	0	4.169925001
21	0	4.807354922
22	0	3.321928095

23	12	3.321928095
24	0	2.584962501
25	0	3.321928095
26	0	5.64385619
27	0	4.169925001
28	0	3
29	0	4.459431619
30	12	4.459431619
31	0	3
1	0	4.584962501
2	0	1
3	0	2.584962501
4	0	1
5	0	2.584962501
6	0	3
7	0	3
8	0	3
9	0	4
10	0	3
11	0	5.087462841
12	0	4
13	0	4.169925001
14	0	4.321928095
15	0	4.584962501
16	0	4.169925001
17	0	4.700439718
18	0	3.584962501
19	0	5.087462841
20	0	5.584962501
21	0	5.087462841
22	0	2.584962501
23	0	4.321928095
24	0	4
25	0	4.584962501
26	0	6.491853096
27	0	5.700439718
28	0	6
29	0	5.906890596
30	0	6.129283017
31	0	5.523561956
1	13	4.906890596
2	0	4.700439718
3	0	4.906890596
4	0	5.087462841
5	0	4.584962501
6	0	4.906890596
7	0	4.807354922
8	0	5.169925001

9	0	4.584962501
10	0	4.459431619
11	0	2.584962501
12	0	3
13	0	3.584962501
14	0	3.807354922
15	0	5.087462841
16	0	4
17	0	3.321928095
19	0	4
20	0	2.584962501
21	13	1
23	33	1
24	33	1
25	28	2.584962501
26	15	2
28	17	1
29	15	1
30	12	1
9	0	3
10	0	4.321928095
11	0	4.459431619
12	0	4
13	0	5.247927513
14	0	4.169925001
15	0	5.95419631
16	0	5.392317423
17	0	5.392317423
18	0	2.584962501
19	0	4.169925001
20	0	3.807354922
21	14	5.459431619
22	21	5.087462841
23	22	5.087462841
24	19	5.392317423
25	24	4
26	16	1
29	19	4.584962501
30	29	3.807354922
31	18	2.584962501
1	15	3.321928095
2	18	2.584962501
3	21	3
4	28	5.087462841
6	12	2
10	39	2
12	46	1
13	25	1

15	0	2.584962501
16	0	2
17	12	2
18	12	1
21	14	1
22	15	2
23	14	3
24	12	3.321928095
25	17	3
26	18	3.321928095
27	11	3.584962501
28	11	2.584962501
29	22	3
30	11	3.807354922
1	13	3.807354922
2	13	3.584962501
3	13	3.321928095
4	14	2
5	27	3.584962501
6	25	2.584962501
7	13	1
8	13	2
11	36	1
14	17	3.321928095
15	25	4.906890596
16	16	3
17	14	3
18	14	4.807354922
19	14	5.754887502
20	30	4.321928095
21	31	3.321928095
22	36	4
23	44	3.321928095
24	47	4
25	39	4.169925001
27	19	3
28	34	1
29	38	1
5	51	2.584962501
6	51	2
7	48	3
8	38	4.169925001
9	55	4.459431619
10	58	4.321928095
11	67	4.169925001
12	50	4.584962501
13	41	4.906890596
14	33	4.700439718

15	38	3.321928095
16	51	3.807354922
17	26	6.672425342
18	23	5.700439718
19	15	5.247927513
20	12	4.807354922
21	0	4.807354922
22	0	4.459431619
23	0	6.357552005
24	12	5.321928095
25	23	6.357552005
26	23	5.392317423
27	12	5.321928095
28	12	4.321928095
29	27	5.64385619
30	29	5.700439718
31	30	5.247927513
1	30	5.087462841
2	58	6.781359714
3	49	7.189824559
4	58	2.584962501
5	43	2
6	29	4
7	14	4
8	0	4.584962501
9	0	4.807354922
10	16	4
11	12	5.906890596
12	13	3.321928095
15	27	2
16	43	1
17	53	2
18	51	2
19	49	2
9	84	1
11	66	2
12	46	2
13	36	1
14	52	2.584962501
15	57	1
16	57	2
17	50	2
18	41	2
21	36	3
22	56	3.321928095
23	31	3.807354922
24	11	3
27	63	1

21	50	1
22	50	2
23	58	3
24	65	3.321928095
25	50	3
26	27	3.321928095
27	15	3.584962501
28	38	2.584962501
29	41	3
30	49	3.807354922
1	56	3.807354922
2	44	3.584962501
3	41	3.321928095
4	46	2
5	32	3.584962501
6	34	2.584962501
7	38	1
8	63	2
10	59	1
14	68	1
16	74	2.584962501
17	93	1
18	115	1
24	43	2
25	29	2.584962501
26	43	3
27	63	3
28	96	2.584962501
29	90	2.584962501
30	91	2
31	99	4.169925001
1	107	6.523561956
2	95	6.129283017
3	81	5.700439718
4	92	5.700439718
5	88	5.64385619
6	79	4.321928095
7	79	4.321928095
8	70	4.169925001
9	61	4
10	36	3.807354922
11	38	3.321928095
12	29	3.584962501
13	18	1
15	12	1
16	31	2
17	51	3.321928095
18	59	3.584962501

19	60	3.584962501
20	59	3.807354922
21	73	4.700439718
22	92	5
23	98	4.906890596
24	70	4.321928095
25	65	3.321928095
26	86	4
27	77	2.584962501
28	64	2
29	56	3.584962501
30	90	3.321928095
31	125	2
1	131	2.584962501
2	136	2.584962501
3	140	2
5	114	2.584962501
6	89	2
7	72	1
8	54	2
11	94	1
13	145	2
14	169	3
15	191	3.321928095
16	191	2.584962501
17	160	2.584962501
18	143	1
19	142	1
5	88	2
6	87	2.584962501
7	80	3
8	85	2.584962501
9	91	2.584962501
10	96	3.584962501
11	110	4.321928095
12	101	4.807354922
13	105	4.169925001
14	119	4.906890596
15	122	5
16	137	3.584962501
17	110	2
18	102	3.584962501
19	109	4.584962501
20	116	4.700439718
21	110	4.321928095
22	80	4
23	77	4.169925001
24	87	4.169925001

25	94	4.169925001
26	80	3.807354922
27	82	3
28	109	2
29	78	2.584962501
30	73	2.584962501
31	89	2
1	121	1
2	151	3.584962501
3	165	3
4	148	2
5	151	1
6	153	1
7	127	2
8	107	1
9	106	1
10	121	1
11	128	2
12	111	2.584962501
13	113	3.584962501
14	123	3.321928095
15	117	3.321928095
16	106	3.321928095
17	88	2
18	57	4.321928095
19	51	4.906890596
20	30	5
21	16	4.321928095
22	16	4.459431619
24	20	4.584962501
25	17	4.459431619
26	36	3.321928095
28	87	3
29	103	3
30	104	2.584962501
1	126	1
2	128	1
3	125	1
6	132	2
8	117	1
9	122	1
11	110	3
12	125	1
13	129	2
14	125	3
15	117	3
16	102	2.584962501
17	86	3.807354922

18	57	3.807354922
19	38	4.169925001
20	36	4.700439718
21	29	5.459431619
22	29	5.807354922
23	63	4.321928095
24	71	5.807354922
25	68	7.169925001
26	86	6.95419631
27	110	6.781359714
28	107	5.64385619
29	111	5.392317423
30	120	4.169925001
31	122	4.700439718
1	132	4.700439718
2	143	4.321928095
3	174	2.584962501
4	152	4.807354922
5	123	4.700439718
6	98	4.321928095
7	122	4.321928095
8	123	4.169925001
9	158	3.807354922
10	131	4.321928095
11	108	3
12	98	2.584962501
13	74	3.584962501
14	33	3.807354922
15	30	5.169925001
16	32	3.321928095
17	50	4.169925001
18	54	3.321928095
19	72	3
20	80	2.584962501
21	71	4.700439718
22	69	4.321928095
23	71	4.700439718
24	71	4.321928095
25	77	4.169925001
26	78	5.169925001
27	75	4.906890596
28	80	4.169925001
29	83	4
30	126	4.321928095
31	150	3.807354922
1	145	3.584962501
2	142	3.807354922
3	171	3.807354922

4	159	4
5	122	3.807354922
6	116	3.807354922
7	95	3.807354922
8	82	4
9	89	3
10	70	3.584962501
11	76	3.321928095
12	73	3
13	58	3
14	52	3.807354922
15	61	2.584962501
16	64	3.321928095
17	55	2.584962501
18	64	2.584962501
19	67	2
20	81	2
21	84	2
22	58	1
23	75	3.807354922
24	95	3.807354922
25	136	4.459431619
26	145	2
27	113	3.807354922
28	85	4.169925001
29	79	3.584962501
11	134	2
12	142	4
13	153	2.584962501
14	160	4.584962501
15	173	2.584962501
16	206	3
17	183	1
18	133	1
19	127	2
20	112	2
21	113	2.584962501
22	131	1
23	108	2.584962501
26	96	2
27	92	1
28	78	2
29	72	2.584962501
30	73	2
1	60	2
3	64	2
4	51	3.321928095
5	79	3.807354922

6	76	4.169925001
7	48	2
8	26	2
9	35	3.807354922
10	26	3
11	16	4.584962501
12	29	3.321928095
13	51	1
15	95	4
16	104	2.584962501
19	123	2.584962501
22	137	1
23	118	1
25	77	2.584962501
27	61	4.169925001
28	75	2.584962501
29	69	2
30	80	3
1	77	3.321928095
2	112	2.584962501
3	109	2
4	121	4.169925001
5	109	2.584962501
6	124	3.321928095
7	141	4.321928095
8	122	3.584962501
9	110	3
10	80	2
11	79	3.584962501
12	73	3.584962501
13	36	3
15	77	2
16	77	3
17	73	2
18	95	2
19	85	1
24	70	1
28	73	2.584962501
29	103	2
30	89	2
5	82	4.459431619
6	71	4.169925001
7	86	3
8	89	4.584962501
9	64	3.321928095
10	72	4
11	81	2.584962501
12	90	4.584962501

13	108	4.700439718
14	108	5.169925001
15	124	4.906890596
16	96	3.807354922
17	100	2
18	110	4.700439718
19	134	2.584962501
20	129	2
21	146	3.584962501
22	139	3
23	138	2
24	78	2.584962501
25	50	2.584962501
26	43	2
27	56	4
28	50	2.584962501
29	53	1
30	71	2.584962501
31	74	2.584962501
1	74	1
2	90	2
3	72	3
4	78	2
5	65	2
6	49	3
7	37	2.584962501
8	25	3.807354922
9	13	2
10	18	3
11	60	2
12	52	2
13	37	1
14	25	1
15	13	2
16	25	2.584962501
15	156	1
18	138	2
19	102	3
20	87	3
21	85	2
22	66	3
23	91	3.807354922
24	109	3
25	109	3.584962501
26	91	3.584962501
27	78	3.321928095
28	70	4.459431619
29	48	3.584962501

30	55	3.584962501
31	57	3
1	64	2
2	62	3
3	74	4.321928095
4	64	3.321928095
5	87	3
6	119	4.169925001
7	133	4.321928095
8	142	4.584962501
9	156	2
10	162	3.584962501
11	174	4.584962501
12	197	4.321928095
13	182	4.169925001
14	137	3
15	74	3.584962501
18	120	2
19	95	3
20	80	3
21	93	3.584962501
22	90	3.807354922
23	77	4.169925001
24	40	4.459431619
25	55	3.807354922
26	67	4.169925001
27	67	4.459431619
28	71	4.584962501
29	93	4.459431619
30	132	4
1	134	2.584962501
2	145	3.321928095
3	167	3
4	172	3
5	194	3.584962501
6	185	3.321928095
7	197	3
8	183	2.584962501
9	162	4.321928095
10	161	3.584962501
11	144	1
13	86	2.584962501
14	62	1
15	18	2
16	10	1
17	0	2.584962501
18	15	2.584962501
19	35	3

20	35	2.584962501
21	17	3
22	39	3
23	65	2
24	62	3
25	64	2
26	58	1
27	78	2
28	109	1
29	137	3
30	122	1
31	131	2
1	165	2
2	165	2
3	153	3
4	146	4.584962501
5	128	5.459431619
6	113	2
7	123	4
8	89	3.584962501
9	83	3
10	62	2.584962501
11	69	1
12	75	3.321928095
13	79	3.807354922
14	86	4
15	107	4.169925001
16	109	3.807354922
17	116	3.321928095
18	105	3.321928095
19	93	5.321928095
20	102	4
21	120	3.584962501
22	125	2
23	150	3
24	149	4.459431619
25	116	4
26	86	5.459431619
27	95	4
28	82	1
29	66	2.584962501
30	76	1
31	82	2
1	96	3
2	110	1
3	102	2.584962501
4	120	2
9	175	3

10	169	2.584962501
11	160	2
12	126	3
16	114	1
17	123	2.584962501
18	82	1
22	101	1
9	139	2.584962501
10	163	2.584962501
11	163	3
13	172	3.321928095
14	164	3
15	124	3.321928095
16	103	4.807354922
18	101	3.584962501
19	77	2.584962501
20	59	3
21	50	4
22	73	3.807354922
23	72	2.584962501
24	78	2
25	67	3.321928095
26	53	4
27	14	3.584962501
28	12	3.321928095
29	33	3.321928095
30	31	2.584962501
31	29	2.584962501
6	109	2.584962501
7	124	3.584962501
8	112	3.321928095
10	93	2.584962501
11	95	3.321928095
13	97	2.584962501
14	90	3.321928095
16	70	3.807354922
17	80	3.321928095
18	77	3.321928095
19	68	1
20	64	2
21	61	1
22	56	2.584962501
23	45	2
26	23	3
27	21	3.584962501
29	39	3.807354922
30	41	4
1	54	3.807354922

2	75	3.584962501
3	88	3.584962501
4	99	4.700439718
5	109	4.169925001
6	97	3.807354922
7	112	3.807354922
8	123	4
9	110	3.321928095
10	103	3
11	92	3.807354922
12	71	3
13	53	3.584962501
14	43	2
16	50	2
17	50	2.584962501
18	58	3
19	44	3
20	36	2.584962501
21	36	2.584962501
22	36	3
23	29	3.807354922
24	31	4.459431619
25	34	5.087462841
26	40	5.459431619
27	51	5.754887502
28	62	5.459431619
29	67	7.321928095
30	76	5.754887502
31	69	5.523561956
1	64	4.906890596
2	53	4.700439718
3	61	5.087462841
4	90	6.906890596
5	94	6.584962501
6	108	6.392317423
7	112	6.247927513
8	93	5.906890596
9	76	5.754887502
10	69	7.247927513
11	78	7.285402219
12	66	6.491853096
13	55	6.906890596
14	46	6.392317423
15	39	7.22881869
16	38	7.108524457
17	33	7.409390936
18	39	7.554588852
19	50	7.266786541

20	65	7.087462841
21	73	7.108524457
22	71	6.087462841
23	81	6.807354922
24	81	6.754887502
25	69	6.459431619
26	47	6.044394119
27	55	5.95419631
28	44	5.459431619
29	56	4.459431619
30	56	5.584962501
31	35	5.169925001
1	43	4.906890596
2	36	4.700439718
3	29	4.584962501
4	37	4
5	27	3
6	43	3
7	44	2
8	42	3.321928095
9	57	2.584962501
10	46	3
11	81	2.584962501
12	89	2
13	83	2
15	68	2
16	86	2
17	75	2.584962501
18	71	2
19	64	3
20	65	2
21	78	3
22	86	2.584962501
23	96	2
24	101	2.584962501
25	152	3
26	162	2.584962501
4	71	1
5	60	1
6	57	1
7	34	3.321928095
8	44	3.321928095
9	52	2
11	79	2
12	83	1
13	78	1
14	88	1
15	87	1

16	55	2
17	38	1
18	30	3.807354922
19	43	2.584962501
21	18	1
22	28	2
24	25	1
28	33	3
29	33	2.584962501
30	41	2.584962501
31	36	2.584962501
1	41	2
2	14	2.584962501
3	0	4.321928095
4	10	2.584962501
5	0	3.584962501
6	0	3.807354922
7	12	3.807354922
8	14	3.807354922
9	19	4
10	33	4.459431619
11	44	4.584962501
12	42	4
13	38	4.169925001
14	38	3
15	40	3.807354922
16	28	6.426264755
17	39	4.459431619
18	47	4.700439718
19	50	2.584962501
20	36	2.584962501
21	24	3
22	23	2.584962501
23	12	2
24	12	3
25	0	4
26	0	4.459431619
27	0	3.807354922
28	0	6.285402219
29	0	4.906890596
30	0	4.700439718
1	0	4.700439718
2	11	5.64385619
3	19	6.392317423
4	0	4.321928095
5	13	7.584962501
6	13	7.22881869
7	25	6.087462841

8	41	6.044394119
9	56	6.459431619
10	50	6.459431619
11	62	6.169925001
12	59	5.087462841
13	53	4.321928095
14	58	3
15	69	5.584962501
16	60	3.584962501
17	39	2.584962501
18	64	4.169925001
19	59	3.584962501
20	56	3.321928095
21	52	3
22	39	2
23	27	3.321928095
24	13	4
25	0	3.584962501
26	0	3.584962501
27	0	3
28	13	3.321928095
29	15	2
30	21	5.169925001
31	16	6.807354922
1	12	6.357552005
2	12	7.06608919
3	0	7.584962501
4	16	8.554588852
5	38	6.285402219
6	39	6.357552005
7	51	6.459431619
8	81	5.754887502
9	72	5.087462841
10	77	3.584962501
11	79	4
12	71	3.321928095
13	58	3.321928095
14	59	3.321928095
15	63	6.392317423
16	80	5.754887502
17	58	7.14974712
18	49	4.906890596
19	47	4.807354922
20	16	5.169925001
21	14	4.169925001
22	28	5.523561956
23	48	5.64385619
24	49	4.321928095

25	48	4.321928095
26	49	3.807354922
27	58	4.169925001
28	64	4.459431619
29	68	3
30	74	2.584962501
31	77	2.584962501
1	78	2
2	67	2.584962501
3	65	3.321928095
4	49	3.321928095
5	31	2.584962501
6	43	3.321928095
7	57	2
8	53	2.584962501
9	79	2
10	78	3.321928095
11	67	2.584962501
12	59	2.584962501
13	39	2
15	15	2
16	13	1
18	38	1
20	53	2
21	55	1
23	60	2
24	55	1
27	26	1
14	0	1
15	0	2.584962501
16	25	3.584962501
17	12	3.321928095
20	30	2.584962501
21	37	3.584962501
22	50	3.807354922
23	55	3.321928095
24	20	3.807354922
25	31	4
26	25	3
27	24	2.584962501
28	21	4
29	14	3
30	0	2.584962501
31	0	2
1	15	4.459431619
2	23	3.584962501
3	24	5
4	25	4.321928095

5	34	5.584962501
6	24	4.906890596
7	14	4.459431619
8	14	4.584962501
9	0	4.169925001
10	0	5.807354922
11	0	6.392317423
12	0	6.247927513
13	11	4
14	11	4
15	31	5.247927513
16	30	3.584962501
17	30	4.584962501
18	29	3.584962501
19	27	2
20	31	1
21	35	1
22	24	1
23	23	3.321928095
24	25	2
25	18	2.584962501
26	21	2.584962501
27	19	3
28	16	2
29	12	4.459431619
30	11	2
1	23	2
2	13	3
3	0	2
4	0	2
5	7	2.584962501
6	16	3.321928095
8	31	2
9	43	3.321928095
10	45	4.169925001
11	38	4.700439718
12	61	3.807354922
13	59	3
14	52	4.459431619
15	33	4.459431619
16	21	4.906890596
17	22	4.321928095
18	0	4.700439718
20	0	2.584962501
21	0	3.807354922
22	0	2.584962501
23	0	3.807354922
24	0	3.321928095

25	12	3
26	12	3
27	0	3
28	11	3.321928095
29	12	3.584962501
30	15	2.584962501
31	0	3
1	13	3.584962501
2	13	5
3	13	3.807354922
4	14	5.247927513
5	13	3.584962501
6	13	5.087462841
7	12	1
8	13	4.169925001
11	12	3.807354922
12	13	5.392317423
13	12	6.247927513
14	13	3.584962501
15	22	4
16	33	3
17	45	3.321928095
18	45	4
19	50	2.584962501
20	60	3.807354922
21	63	3.807354922
22	63	3
23	69	2.584962501
24	54	2.584962501
25	53	2.584962501
26	48	2
27	38	3
28	23	1
29	40	3
31	73	2
1	59	2
2	56	1
4	112	1
5	119	2
7	97	2.584962501
8	88	2.584962501
9	62	1
11	29	1
12	11	1
13	12	1
17	0	1
18	0	1
20	0	2.584962501

22	13	2.584962501
24	33	2.584962501
26	27	1
31	22	2
3	13	2.584962501
8	0	1
9	0	2
13	15	1
14	15	2
15	14	2
17	14	1
10	0	1
12	0	1
13	13	2
14	13	1
15	12	1
17	0	1
18	0	1
19	0	2.584962501
20	0	1
21	12	1
22	0	1
24	0	3
29	0	1
30	0	1
1	12	2
2	11	1
3	0	2.584962501
4	0	3
5	0	1
7	0	1
8	0	2
9	0	2.584962501
10	0	2.584962501
11	0	1
12	0	2
13	0	1
14	12	1
16	13	2
17	12	1
18	11	3.584962501
19	14	2
20	16	1
21	16	2
22	12	1
23	15	2.584962501
24	29	2
25	33	1

26	28	1
27	13	2
28	12	3
29	0	3.321928095
30	0	2
2	0	1
6	0	1
9	14	2.584962501
11	16	1
