# Supplementary material for: Can Sunspot Activity Affect the Population Dynamics of Cotton Bollworm, Helicoverpa armigera (Hübner) (Lepidoptera: Noctuidae)?
Source: Insects. 2025 Aug 15;16(8):846. doi: 10.3390/insects16080846 (PMC12386618; doi:10.3390/insects16080846)
Supplement: Supplementary file 1 [file insects-16-00846-s001.zip › Table S2. log2(moths No.)+1 for moth in Maigaiti, Bachu and Shawan.pdf]

Maigaiti	year-month	sunspot	log2(moths No.)+1
	199005	175.2	4.459431619
	199006	153.3	5.247927513
	199007	191.1	4.169925001
	199008	252.1	5.321928095
	199105	166.9	3.807354922
	199106	224.7	4.584962501
	199107	240.2	4.807354922
	199108	240.8	4.321928095
	199109	168.9	4.321928095
	199205	94.3	5.459431619
	199206	98.5	1
	199207	114.2	2.584962501
	199208	91.9	6.357552005
	199209	94	9.640244936
	199305	78.8	2.584962501
	199306	69.6	8.77478706
	199307	80.4	9.189824559
	199308	62.5	11.59898297
	199309	31.2	9
	199404	27.4	6
	199405	29.8	6.459431619
	199406	39.7	4.169925001
	199407	50.6	4.459431619
	199408	34.3	6.392317423
	199409	40.5	7.442943496
	199505	19.4	4.169925001
	199506	22.5	4.459431619
	199507	20.4	4.459431619
	199508	18.2	7.087462841
	199509	15.7	5.754887502
	199605	7.6	4.906890596
	199606	16.5	3
	199607	11.8	7.87036472
	199608	19.7	10.95128471
	199609	3	9.113742166
	199704	23	3.321928095
	199705	25.4	4
	199706	20.8	5.64385619
	199707	12.9	7.50779464
	199708	35.7	8.055282436
	199709	59.7	6.321928095
	199804	70.6	3.807354922
	199805	74	5.247927513
	199806	90.5	7.392317423
	199807	96.7	5.857980995
	199808	121.1	9.011227255
	199809	132	8.651051691

Bachu	year-montl	sunspot
	199105	1
	199106	4.459432
	199107	3.70044
	199108	3.584963
	199109	2
	199206	1.584963
	199207	3.70044
	199208	3
	199304	1
	199305	0
	199306	4.807355
	199307	3.459432
	199308	3.807355
	199309	3.169925
	199405	4.321928
	199406	4.584963
	199407	3
	199408	2
	199409	1.584963
	199505	5.72792
	199506	6.658211
	199507	6.228819
	199508	2.584963
	199509	2.807355
	199605	4.169925
	199606	3.169925
	199607	5
	199608	4.169925
	199704	3.70044
	199705	4.807355
	199706	5.882643
	199707	5.285402
	199708	4.906891
	199709	4.523562
	199804	2.321928
	199805	4
	199806	3.807355
	199807	3.459432
	199808	5.357552
	199809	3.321928
	199904	3.459432
	199905	4.459432
	199906	5.906891
	199907	5.426265
	199908	7.303781
	199909	1.584963
	200004	2

199904	93.6	3.321928095
199905	149.6	7.247927513
199906	207.2	9.098032083
199907	173.5	9.266786541
199908	142.3	11.34207467
199909	106.3	8.348728154
199910	168.7	2
200004	191.5	4.169925001
200005	165.9	7.209453366
200006	188	11.02652344
200007	244.3	12.10525378
200008	180.5	11.76735685
200009	156	9.044394119
200104	161.7	4.321928095
200105	142.1	6.285402219
200106	202.9	8.266786541
200107	123	9.707359132
200108	161.5	10.58308277
200109	238.2	7.930737338
200110	194.1	5.169925001
200204	186.9	4
200205	187.5	6.459431619
200206	128.8	10.20212382
200207	161	8.721099189
200208	175.6	10.93957921
200209	187.9	8.839203788
200210	151.2	5.700439718
200304	97.9	1
200305	86.8	6.285402219
200306	118.7	7.820178962
200307	128.3	9.335390355
200308	115.4	10.39016896
220309	78.5	8.994353437
200310	97.8	6.392317423
200404	59.2	6.169925001
200405	72.8	8.832890014
200406	66.5	11.13057056
200407	83.8	9.994353437
200408	69.7	10.93663794
200409	48.8	9.124121312
200410	74.2	2
200504	38.7	4.459431619
200505	61.9	7.845490051
200506	56.8	9.417852515
200507	62.4	8.965784285
200508	60.5	10.49585503
200509	37.2	9.459431619
200510	13.2	2

200005	3.807355
200006	7.930737
200007	5.285402
200008	5.426265
200009	1
200104	2
200105	2.584963
200106	5.97728
200107	4
200108	3.906891
200204	0
200205	3.169925
200206	6.33985
200207	2.807355
200208	9.643856
200209	6.129283
200304	2.584963
200305	2
200306	4.906891
200307	5.857981
200308	6.954196
200309	5.247928
200404	1.584963
200405	1.584963
200406	2.584963
200407	2.321928
200408	5.169925
200409	3
200410	0
200504	1.584963
200505	4.247928
200506	3.70044
200507	4.169925
200508	4.70044
200604	1
200605	2
200606	1
200607	2.807355
200608	5.169925
200609	6.169925
200610	0
200704	2
200705	3.807355
200706	4.247928
200707	3.321928
200708	5.392317
200804	1.584963
200805	4.459432

200604	50.3	4.321928095
200605	37.2	9.022367813
200606	24.5	11.11113567
200607	22.2	10.09011242
200608	20.8	13.22219114
200609	23.7	9.710806434
200610	14.9	5.169925001
200704	5.4	5.64385619
200705	19.5	8.95419631
200706	21.3	11.33873638
200707	15.1	10.34207467
200708	9.8	13.40779885
200709	4	10.21674586
200710	1.5	5.64385619
200804	3.6	4.169925001
200805	4.6	6.754887502
200806	5.2	10.06339508
200807	0.6	9.011227255
200808	0.3	11.04302728
200809	1.2	9.409390936
200904	1.2	6.523561956
200905	2.9	8.383704292
200906	6.3	11.19475685
200907	5.5	11.4252159
200908	0	11.31061278
200909	7.1	8.383704292
201004	10.4	4.169925001
201005	13.9	8.491853096
201006	18.8	9.124121312
201007	25.2	11.05934446
201008	29.6	11.56890615
201009	36.4	10.50183718
201104	76.1	2.584962501
201105	58.2	8.303780748
201106	56.1	10.40514146
201107	64.5	9.54303182
201108	65.8	10.82336724
201109	120.1	8.832890014
201204	85.9	3.584962501
201205	96.5	7.108524457
201206	92	7.820178962
201207	100.1	8.285402219
201208	94.8	9.675957033
201209	93.7	7.357552005
201304	107.3	6.285402219
201305	120.2	8.108524457
201306	76.7	8.665335917
201307	86.2	7.523561956

200806	5.72792
200807	5.459432
200808	6.988685
200809	5.491853
200904	3
200905	0
200906	5.754888
200907	4.087463
200908	3.459432
200909	3.459432
201004	2
201005	3
201006	3.70044
201007	4.392317
201008	3.807355
201104	1
201105	5.78136
201106	4.906891
201107	6.285402
201108	5.491853
201109	3.906891
201204	2
201205	5.70044
201206	5.491853
201207	5.554589
201208	6.714246
201209	6.044394
201304	1.584963
201305	4.857981
201306	4.857981
201307	6.066089
201308	7.643856
201309	5.321928
201310	2
201404	1.584963
201405	4.392317
201406	4.70044
201407	5.129283
201408	6.409391
201409	5.523562
201410	2
201505	4.169925
201506	5.321928
201507	4.459432
201508	6.149747
201509	5.321928

201308	91.8	9.607330314
201309	54.5	4.321928095
201404	112.5	4
201405	112.5	7.189824559
201406	102.9	5.169925001
201407	100.2	7.108524457
201408	106.9	8.936637939
201409	130	6.672425342
201504	75.3	1
201505	88.8	7.189824559
201506	66.5	8.294620749
201507	65.8	9.184875343
201508	64.4	9.813781191
201509	78.6	8.426264755
201604	37.9	3.321928095
201605	51.5	5.584962501
201606	20.5	9.475733431
201607	32.4	7.426264755
201608	50.2	8.614709844
201609	44.6	5.247927513
201704	32.3	4.169925001
201705	18.9	9.169925001
201706	19.2	9.710806434
201707	17.8	10.31061278
201708	32.6	10.12928302
201709	43.7	7.50779464

log2(moths No.)+1	Shawan	year-montl	sunspot	log2(moths No.)+1
2		199506	22.5	2.584962501
5.459431619		199507	20.4	5.392317423
4.700439718		199605	7.6	4
4.584962501		199606	16.5	7.129283017
3		199607	11.8	5.754887502
2.584962501		199608	19.7	8.409390936
4.700439718		199705	25.4	5.906890596
4		199706	20.8	6.247927513
2		199707	12.9	7.108524457
1		199708	35.7	8.693486957
5.807354922		199709	59.7	4.169925001
4.459431619		199805	74	4
4.807354922		199806	90.5	6.727920455
4.169925001		199807	96.7	7
5.321928095		199808	121.1	9.21916852
5.584962501		199809	132	8.64385619
4		200005	165.9	6.554588852
3		200006	188	5.087462841
2.584962501		200007	244.3	8.033423002
6.727920455		200008	180.5	7.614709844
7.658211483		200009	156	1
7.22881869		200105	142.1	1
3.584962501		200106	202.9	7.169925001
3.807354922		200107	123	6.64385619
5.169925001		200108	161.5	7.727920455
4.169925001		200109	238.2	3
6		200206	128.8	6.129283017
5.169925001		200207	161	5.754887502
4.700439718		200208	175.6	6.491853096
5.807354922		200209	187.9	6.129283017
6.882643049		200305	86.8	3.321928095
6.285402219		200306	118.7	5.523561956
5.906890596		200307	128.3	2.584962501
5.523561956		200308	115.4	5.906890596
3.321928095		200309	78.5	5.95419631
5		200405	72.8	2.584962501
4.807354922		200406	66.5	4.584962501
4.459431619		200407	83.8	5.700439718
6.357552005		200408	69.7	9.463524373
4.321928095		200409	48.8	9.118941073
4.459431619		200505	61.9	3.807354922
5.459431619		200506	56.8	7.189824559
6.906890596		200507	62.4	7.820178962
6.426264755		200508	60.5	9.764871591
8.303780748		200509	37.2	4.807354922
2.584962501		200605	37.2	5.95419631
3		200606	24.5	7.303780748

4.807354922	200607	22.2	7.189824559
8.930737338	200608	20.8	8.761551232
6.285402219	200609	23.7	4.321928095
6.426264755	200705	19.5	6.209453366
2	200706	21.3	6.209453366
3	200707	15.1	7.129283017
3.584962501	200708	9.8	10.76983784
6.977279923	200709	4	6.584962501
5	200805	4.6	9.199672345
4.906890596	200806	5.2	9.971543554
1	200807	0.6	11.74483384
4.169925001	200808	0.3	12.05052891
7.339850003	200809	1.2	6.930737338
3.807354922	200905	2.9	8.77478706
10.64385619	200906	6.3	7.614709844
7.129283017	200907	5.5	9.011227255
3.584962501	200908	0	9.6794801
3	200909	7.1	8.741466986
5.906890596	201005	13.9	9.027905997
6.857980995	201006	18.8	7.392317423
7.95419631	201007	25.2	8.257387843
6.247927513	201008	29.6	9.839203788
2.584962501	201009	36.4	8.930737338
2.584962501	201105	58.2	6.209453366
3.584962501	201106	56.1	6.357552005
3.321928095	201107	64.5	7.108524457
6.169925001	201108	65.8	9.348728154
4	201109	120.1	6.169925001
1	201205	96.5	8.569855608
2.584962501	201206	92	8.21916852
5.247927513	201207	100.1	9.665335917
4.700439718	201208	94.8	9.154818109
5.169925001	201209	93.7	8.294620749
5.700439718	201305	120.2	6.781359714
2	201306	76.7	7.569855608
3	201307	86.2	7.392317423
2	201308	91.8	8.451211112
3.807354922	201309	54.5	6.392317423
6.169925001	201405	112.5	7.169925001
7.169925001	201406	102.9	8.686500527
1	201407	100.2	7.614709844
3	201408	106.9	8.700439718
4.807354922	201409	130	5.807354922
5.247927513	201505	88.8	7.727920455
4.321928095	201506	66.5	7.339850003
6.392317423	201507	65.8	9.451211112
2.584962501	201508	64.4	11.50680344
5.459431619	201509	78.6	7.794415866

6.727920455	201605	51.5	6.672425342
6.459431619	201606	20.5	9.144658243
7.988684687	201607	32.4	10.34872815
6.491853096	201608	50.2	10.79766153
4	201609	44.6	6.754887502
1	201705	18.9	7.22881869
6.754887502	201706	19.2	9.312882955
5.087462841	201707	17.8	8.434628228
4.459431619	201708	32.6	8.787902559
4.459431619	201709	43.7	4.906890596
3	201805	13.1	4.906890596
4	201806	15.6	4.584962501
4.700439718	201807	1.6	5.321928095
5.392317423	201808	8.8	6.906890596
4.807354922	201809	3.3	3.584962501
2	201905	9.9	5.754887502
6.781359714	201906	1.2	5.247927513
5.906890596	201907	0.9	4.906890596
7.285402219	201908	0.5	5.523561956
6.491853096			
4.906890596			
3			
6.700439718			
6.491853096			
6.554588852			
7.714245518			
7.044394119			
2.584962501			
5.857980995			
5.857980995			
7.06608919			
8.64385619			
6.321928095			
3			
2.584962501			
5.392317423			
5.700439718			
6.129283017			
7.409390936			
6.523561956			
3			
5.169925001			
6.321928095			
5.459431619			
7.14974712			
6.321928095			
