# Supplementary material for: Can Sunspot Activity Affect the Population Dynamics of Cotton Bollworm, Helicoverpa armigera (Hübner) (Lepidoptera: Noctuidae)?
Source: Insects. 2025 Aug 15;16(8):846. doi: 10.3390/insects16080846 (PMC12386618; doi:10.3390/insects16080846)
Supplement: Supplementary file 1 [file insects-16-00846-s001.zip › Table S3. Relative catching of moths in Maigaiti, Bachu and Shawan.pdf]

| sites | Rc1 | Rc2 | Rc3 | Rc4 | Rc5 | Rc6 |
|---|---|---|---|---|---|---|
| Maigaiti | 1.0658±0.568[illegible] | 1.0820±0.609[illegible] | 1.0063±0.5888 | 1.0667±0.5434 | 1.0185±0.4868 | 0.9618±0.513[illegible] |
| Bachu | 0.9875±0.043[illegible] | 0.9365±0.0347 | 0.8690±0.0453 | 0.9234±0.0465 | 0.9628±0.0616 | 0.9539±0.053[illegible] |
| Shawan | 1.2072±0.030[illegible] | 1.0005±0.0292 | 1.0011±0.0390 | 1.0382±0.0446 | 1.0077±0.0488 | 0.9624±0.039[illegible] |

| Rc7 | Rc8 | Rc9 | Rc10 | Rc11 | Rc12 | Rc13 |
| --- | --- | --- | --- | --- | --- | --- |
| 1.0117±0.5232 | 0.9368±0.5254 | 0.8875±0.4913 | 0.9059±0.5095 | 0.3105±0.1699 | 0.9645±0.5095 | 0.8906±0.0451 |
| 0.9328±0.0539 | 1.0262±0.0621 | 1.1186±0.0741 | 0.9889±0.0740 | 1.0696±0.0496 | 1.0007±0.0624 | 0.9818±0.0599 |
| 1.0799±0.0468 | 1.0097±0.0380 | 0.9514±0.0569 | 0.9283±0.0542 | 0.8964±0.0347 | 0.8745±0.0366 | 0.8496±0.0427 |

| Rc14 | Rc15 | Rc16 | Rc17 | Rc18 |
|---|---|---|---|---|
| 1.0197±0.5594 | 0.9901±0.5676 | 1.0005±0.0759 | 0.9811±0.0650 | 0.9365±0.0946 |
| 0.9479±0.0652 | 1.3090±0.1247 | 1.1167±0.1286 | 1.2950±0.1719 | 1.2660±0.1435 |
| 0.7576±0.0466 | 0.2239±0.0253 | 0.8240±0.0972 | 0.7662±0.1239 | 0.7562±0.0969 |
