# Supplementary material for: Can Sunspot Activity Affect the Population Dynamics of Cotton Bollworm, Helicoverpa armigera (Hübner) (Lepidoptera: Noctuidae)?
Source: Insects. 2025 Aug 15;16(8):846. doi: 10.3390/insects16080846 (PMC12386618; doi:10.3390/insects16080846)
Supplement: Supplementary file 1 [file insects-16-00846-s001.zip › Table S4. Sunspot, annual Tmean, log2(moths number) in three regions.pdf]

year	sunspot	annual Tmean in Maiga	annual Tmean in Bach	annual Tmean in Shawar
1989	211.1	11.45	11.89	8.44
1990	191.8	12.27	13.05	8.61
1991	203.3	11.12	11.82	8.7
1992	133	11.3	12.03	4.56
1993	76.1	11.56	12.66	6.95
1994	44.9	12.06	11.82	7.86
1995	25.1	11.12	11.36	8.47
1996	11.6	10.87	12.92	7.39
1997	28.9	12.25	12.65	9.25
1998	88.3	12.27	12.95	8.63
1999	136.3	12.37	12.73	8.8
2000	173.9	12.22	12.94	8.41
2001	170.4	12.27	12.86	8.81
2002	163.6	12.07	12.43	8.94
2003	99.3	11.93	13.35	7.92
2004	65.3	12.42	12.46	8.81
2005	45.8	11.96	12.74	8.34
2006	24.7	12.16	13.37	9.44
2007	12.6	12.97	12.46	9.11
2008	4.2	12.05	13.15	9.33
2009	4.8	12.93	12.53	8.88
2010	24.9	12.38	12.45	7.84
2011	80.8	12.28	12.45	8.23
2012	84.5	11.3	11.67	8.18
2013	94	12.65	12.97	9.49
2014	113.3	12.47	12.21	8.27
2015	69.8	13.34	13.31	9.55
2016	39.8	13.45	13.29	8.71
2017	21.7	12.96	13.02	9.08
2018	7	12.9	12.43	7.9
2019	3.6			
2020	8.8			
2021	29.6			
2022	83.2			
2023	125.5			
2024	154.7			

ı	annual log2(moth No.)+1 in Maig	annual log2(moth No.)+1 in Bac
	6.584962501	
	6.882643049	
	6.727920455	5.584962501
	9.864186145	5.700439718
	12.18053081	6.832890014
	8.569855608	6.906890596
	7.977279923	8.918863237
	11.45429929	7.266786541
	9.238404739	8.599912842
	10.22400167	7.599912842
	12.05392588	9.209453366
	13.38154295	9.434628228
	11.57742883	7.727920455
	12.02859678	10.79116289
	11.49585503	9.14974712
	12.62388149	6.95419631
	11.80896417	7.303780748
	13.8008999	7.95419631
	14.01384605	7.475733431
	12.02997735	9.204571144
	13.03514275	7.672425342
	12.8837888	7.108524457
	12.1959873	8.813781191
	10.70563239	9.317412614
	10.76818432	9.54303182
	9.857980995	8.781359714
	11.15987134	8.577428828
	10.42206477	
	11.96722626	

annual log2(moth No.)+1 in Shawan

9.103287808
9.463524373
10.28308835
8.584962501
9.199672345
8.864186145
8.14974712
7.523561956
10.39231742
10.32642949
9.6794801
11.05663772
13.19813805
11.22761594
11.22881869
9.991521846
11.21310422
9.816983623
10.25974326
12.03823313
11.91438513
10.64745843
7.820178962
